# Retinal ganglion cell-derived semaphorin 6A segregates starburst amacrine cell dendritic scaffolds to organize the mouse inner retina

**DOI:** 10.1242/dev.204293

**Published:** 2024-11-26

**Authors:** Rebecca E. James, Natalie R. Hamilton, Lola Nicole Huffman, Matthew P. Brown, Victoria N. Neckles, R. Jeroen Pasterkamp, Loyal A. Goff, Alex L. Kolodkin

**Affiliations:** ^1^ The Solomon H. Snyder Department of Neuroscience, Johns Hopkins Kavli Neuroscience Discovery Institute, The Johns Hopkins School of Medicine, 725 North Wolfe Street, Baltimore, MD 21205, USA; ^2^ Department of Translational Neuroscience, University Medical Center Utrecht Brain Center, Utrecht University, Universiteitsweg 100, 3584 CG, Utrecht, The Netherlands

**Keywords:** Retinal patterning, Starburst amacrine cells, Sema6A, Direction selectivity

## Abstract

To form functional circuits, neurons must settle in their appropriate cellular locations, and then project and elaborate neurites to contact their target synaptic neuropils. Laminar organization within the vertebrate retinal inner plexiform layer (IPL) facilitates pre- and postsynaptic neurite targeting, yet the precise mechanisms underlying establishment of functional IPL subdomains are not well understood. Here, we explore mechanisms defining the compartmentalization of OFF and ON neurites generally, and OFF and ON direction-selective neurites specifically, within the developing mouse IPL. We show that semaphorin 6A (Sema6A), a repulsive axon guidance cue, is required for delineation of OFF versus ON circuits within the IPL: in the *Sema6a* null IPL, the boundary between OFF and ON domains is blurred. Furthermore, Sema6A expressed by retinal ganglion cells (RGCs) directs laminar segregation of OFF and ON starburst amacrine cell dendritic scaffolds, which themselves serve as a substrate upon which other retinal neurites elaborate. These results demonstrate that RGCs, the first type of neuron born within the retina, play an active role in functional specialization of the IPL.

## Teaser

**Summary:** Semaphorin 6A expressed by retinal ganglion cells directs laminar segregation of OFF and ON starburst amacrine cell dendritic scaffolds, playing an active role in specialization of ON versus OFF domains of the inner plexiform layer.

## INTRODUCTION

Elaboration of neural circuitry is facilitated by restricted synapse formation between distinct neuronal cell types within specific layers of laminated regions in the nervous system, and early born neurons can provide a scaffold upon which later born neurons elaborate their neurites. The vertebrate retina includes bipolar cells (BCs), amacrine cells (ACs) and retinal ganglion cells (RGCs), which synaptically connect in ∼10 sublaminae within the inner plexiform layer (IPL). Understanding how retinal laminar organization develops has been guided by work on direction-selective (DS) circuits, which activate direction-selective ganglion cells (DSGCs) to respond to image motion.

The IPL is divided into OFF and ON subregions reflecting responses to the diminution or enhancement of illumination, respectively (Zhang et al., 2017). ON-OFF DSGCs (ooDSGCs) tuned to fast motion in the four cardinal directions have dendrites stratifying in both OFF (sublayer S2) and ON (S4) IPL sublayers (Dhande et al., 2015). DSGCs tuned to slower motion that stabilize images on the retina, ON DSGCs (oDSGCs), belong to the accessory optic system (AOS) and stratify in S4 (Hamilton et al., 2021). Projections from select classes of BCs and ACs connect with DSGCs in S2 and S4, including OFF-starburst amacrine cell (SAC) and ON-SAC subtypes, which stratify their dendrites in S2 or S4, respectively. SACs are among the first neurons to elaborate neurites (Stacy and Wong, 2003). As their dendritic arbors develop, they form the S2 and S4 IPL sublaminae, which serve as scaffolds that instruct subsequent DSGC and BC neurite stratification (Ray et al., 2018; Duan et al., 2018; Peng et al., 2017). In zebrafish, RGCs serve a transient role in organizing the earliest AC projections within the IPL, suggesting that signals from RGCs impact AC neurite guidance during early stages of IPL lamination (Kay et al., 2004).

Several molecular cues contribute to murine DS circuit development. SAC cell body settling in the inner nuclear layer (INL; OFF-SACs) or the ganglion cell layer (GCL; ON-SACs), and SAC mosaic cell body spacing within these layers, requires the cell-surface protein MEGF10 (Kay et al., 2012). In SACs, homotypic dendrite self-avoidance requires protocadherins (Lefebvre et al., 2012). Classical cadherins and contactin cell-adhesion molecules mediate BC axon and DSGC dendrite targeting to the SAC scaffolds (Duan et al., 2014, 2018; Peng et al., 2017). Interactions between the fibronectin leucine-rich repeat transmembrane 2 (FLRT2) receptor and the transmembrane UNC5 ligand abrogate FLRT2-latrophilin (LPHN) adhesive interactions, influencing DSGC dendrite targeting to correct IPL strata (Prigge et al., 2023). The transmembrane protein semaphorin 6A (Sema6A) and its plexin A2 (PlexA2) receptor also play key roles. Sema6A is enriched in ON regions of the IPL (Matsuoka et al., 2011a), and *Sema6a* loss of function leads to mis-stratification of SAC dendrites, disruption of ON-SAC dendritic arbor morphology, and loss of ON, but not OFF, ooDSGC DS tuning responses (Sun et al., 2013). However, the cell type(s) in which Sema6A is required for SAC development is unknown due to use, thus far, of a global *Sema6a* loss-of-function allele.

Here, we revisit Sema6A–PlexA2 signaling in retinal development using newly generated mouse models that allow cell type-specific assessments of Sema6A function. We refine our understanding of Sema6A activities during SAC development and uncover distinct functions for Sema6A forward and reverse signaling in aspects of DS circuit formation. We reveal previously unreported roles for Sema6A in RGCs to direct SAC dendrite lamination and to delimit the ON regions of the IPL. These results provide insight into the development of retinal laminar organization.

## RESULTS

### *Sema6a* is not required in SACs for dendritic arbor lamination

**Fig. 1.**
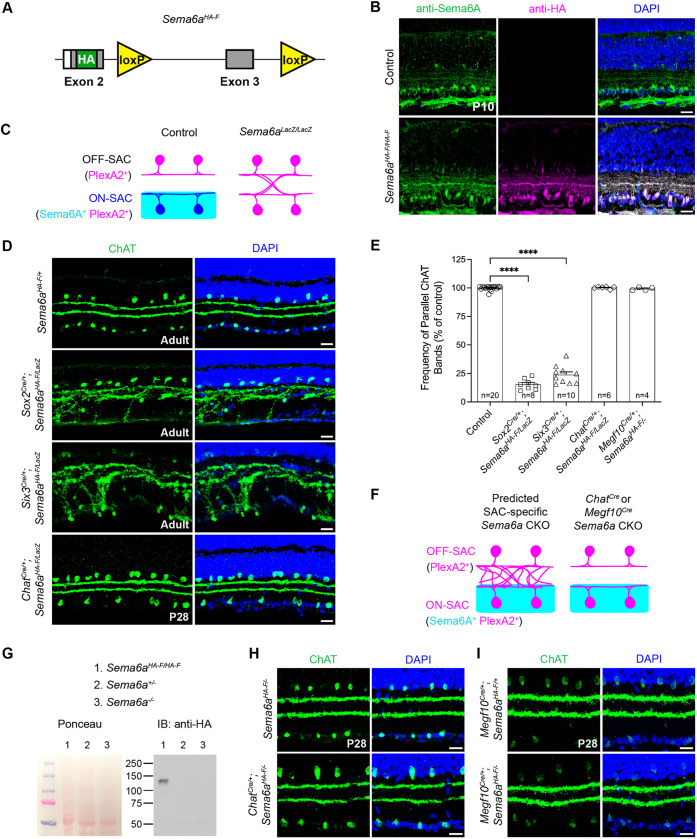
**SAC-derived Sema6A does not contribute to SAC layer-specific dendrite targeting.** (A) *Sema6a* conditional allele design. Cre-mediated loxP recombination generates a frameshift and premature stop codons. (B) Anti-Sema6A and anti-HA in control (C57BL/6) and *Sema6a^HA-F/HA-F^* retinas. (C) Model of Sema6A–PlexA2 expression and SAC lamination defects previously observed. (D) SAC (anti-ChAT) dendrite lamination for the indicated genotypes. (E) Quantification of the frequency of segregated ChAT^+^ SAC strata. Controls were pooled. (F) Predicted and actual SAC-cKO SAC lamination defects. (G) Cortical lysate western blot for HA-Sema6A (∼130 kDa). Ponceau S, loading control. (H,I) SAC lamination in neonatal (*Chat^Cre^*) (H) and embryonic (*Megf10C^re^*) (I) SAC-cKO retinas. *n*, number of retinas. ****P<0.0001; one-way ANOVA, Dunnett's multiple comparison test (MCT). Scale bars: 20 µm.

To investigate cell type-specific requirements for *Sema6a* in retinal development, we generated a new HA-epitope knock-in *Sema6a* conditional allele (*Sema6a^HA-F^*) that produces N-terminal HA-tagged Sema6A (HA-Sema6A) in the absence of Cre recombinase and does not affect overall retinal lamination (Fig. 1A; Fig. S1A-C). HA-Sema6A expression mirrors endogenous anti-Sema6A (Kerjan et al., *2*005) immunolabeling at postnatal day 10 (P10) (Fig. 1B). This Sema6A antibody can be unreliable, often yielding a speckled, non-specific signal that fails to recapitulate wild-type Sema6A both *in vivo* and *in vitro* (Fig. S1D,E). HA-Sema6A immunolabeling, however, specifically and reproducibly reports Sema6A expression (Fig. 1B).

Based on previous observations using the *Sema6a* gene trap loss-of-function allele (*Sema6a^LacZ^*) (Leighton et al., 2001; Matsuoka et al., 2011a; Sun et al., 2013), we hypothesized that Sema6A in ON-SACs repels OFF-SAC dendrites to segregate the SAC IPL layers (Fig. 1C). To assess cell type-specific roles for Sema6A, we first crossed the *Sema6a^HA-F^* allele to a germline Cre driver (*Sox2^Cre^*; Hayashi et al., 2002) to determine its recombination efficacy. Unexpectedly, germline *Sema6a* loss of function resulted in more severe SAC lamination defects than previously observed in *Sema6a^LacZ/LacZ^* retinas (Sun et al., 2013): SAC dendrite scaffolds were nearly fused (Fig. 1D,E). This fusion phenotype was also evident in the central retina of *Six3^Cre/+^* (Furuta et al., 2000) conditional knockout (cKO) retinas, where Cre is strongly expressed in central, but not peripheral, retinal progenitors (Ray et al., 2018). We next removed *Sema6a* from SACs using *Chat^Cre^* (Rossi et al., 2011), anticipating similar SAC lamination defects (Fig. 1F), yet SAC dendrites were appropriately segregated (Fig. 1D,E).

Might the *Sema6a^LacZ^* allele in *Sema6a^HA-F/LacZ^* mice mask expressivity of lamination defects in *Chat^Cre^* cKO retinas? We examined SAC dendrite segregation in a *Chat^Cre/+^; Sema6a^HA-F^*^/−^ background heterozygous for a *Sema6a* null allele that we generated by germline recombination of *Sema6a^HA-F^* using *Sox2^Cre^* [this new null allele does not produce Sema6A protein (Fig. 1G)], yet SAC IPL lamination was unaffected (Fig. 1H). Since *Chat^Cre^* expression coincides with SAC dendrite lamination (Ray et al., 2018), we used *Megf10C^re^* (Kozlowski et al., 2023 preprint), which is expressed exclusively in SACs embryonically (E16.5) (Peng et al., 2020). Embryonic *Sema6a* cKO in SACs also did not alter SAC lamination (Fig. 1I), demonstrating that SAC-derived Sema6A is dispensable for SAC dendrite segregation.

### *Sema6a* in SACs regulates SAC dendrite arbor elaboration

**Fig. 2.**
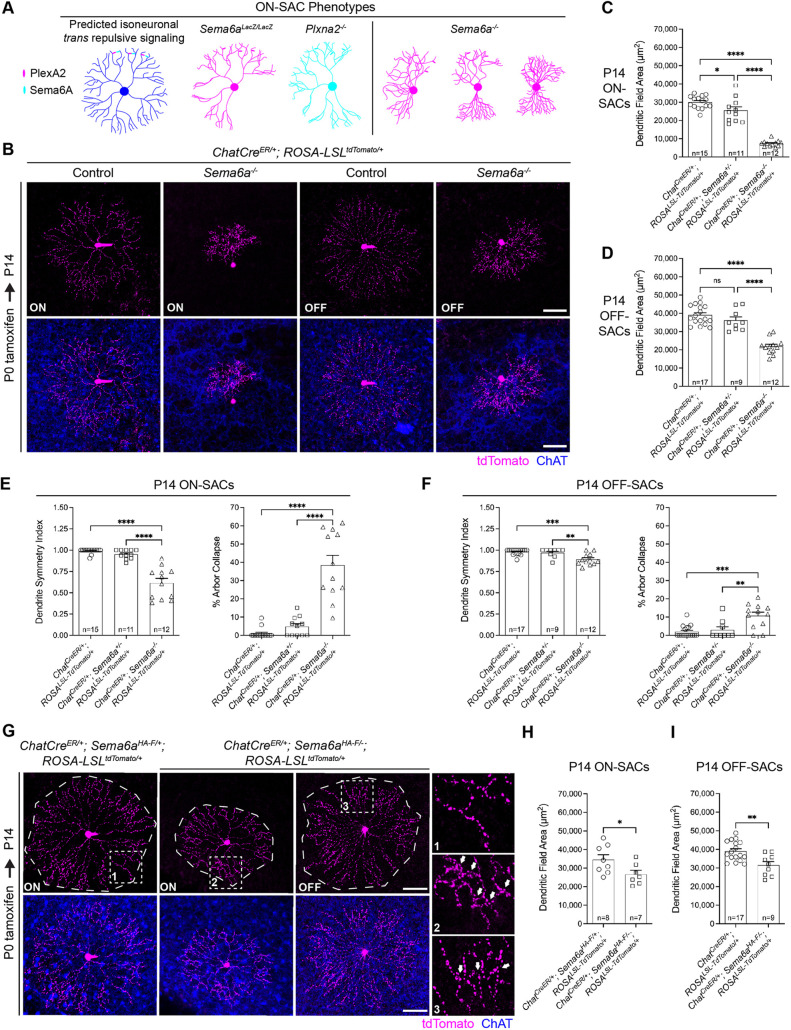
**Sema6A in SACs promotes neurite outgrowth and distal dendrite self-avoidance.** (A) Model of predicted isoneuronal ON-SAC Sema6A–PlexA2 signaling and observed SAC morphological phenotypes. (B) tdTomato-labeled SACs. Control, *Sema6a^+/+^*. (C,D) Quantification of ON-SAC (C) and OFF-SAC (D) dendrite area. (E,F) ON-SAC (E) and OFF-SAC (F) dendritic symmetry and % arbor collapse. (G) tdTomato-labeled, singly targeted *Sema6a* cKO ON- and OFF-SACs. Arrows indicate dendrite self-crossover events. (H,I) Quantification of cell-autonomous impacts of sparse *Sema6a* cKO on ON-SAC (H) and OFF-SAC (I) dendritic field area. The OFF-SAC control in D was used for the comparison in I. *n*, number of SACs. *P<0.05; **P<0.01; ***P<0.001; ****P<0.0001; one-way ANOVA, Tukey's MCT. Scale bars: 50 µm.

Sema6A and PlexA2 contribute to ON-SAC dendritic arbor radial symmetry (Sun et al., 2013), suggesting that isoneuronal *trans* repulsive signaling promotes sister dendrite self-avoidance (Fig. 2A). In *Sema6a^LacZ^* mutants, ON-SACs exhibited self-avoidance defects and reduced the dendrite arbor area (∼60%), while OFF-SACs developed normally, although in *Plxna2^−/−^* retinas the OFF-SAC dendrite arbor area was also reduced (∼40%; Sun et al., 2013). We examined morphology in P14 *Sema6a^−/−^* SACs by sparsely recombining *ROSA-LSL^tdTomato^* (Madisen et al., 2010) using *Chat^CreER^* (Rotolo et al., 2008). We observed a 75% reduction in the ON-SAC dendrite arbor area, and the OFF-SAC arbor area was reduced by 45% (Fig. 2B-D). A range of SAC symmetry deficits was observed in *Sema6a^−/−^* retinas (Fig. 2A,B; Fig. S2A). ON-SAC symmetry was more severely disrupted than OFF-SAC symmetry (Fig. 2E,F). Using Cre-dependent *AAV2-FLEX-GFP* viral labeling, we confirmed the persistence of ON-SAC arbor deficits into adulthood (Fig. S2A-C). Dendrite self-avoidance errors, such as those in *Sema6a^LacZ^* mutant ON-SACs (Sun et al., 2013), were evident in both P14 and P42 ON-SACs, and also in P14 OFF-SACs (Fig. 2B). Since the SAC strata are effectively fused in *Sema6a^−/−^* retinas (Fig. 1, see Fig. 5), we were unable to examine gross ON- versus OFF-SAC dendrite plexus organization. *Sema6a^+/−^* heterozygous ON-SAC dendrite self-avoidance (Fig. S2A,B; see inset) and arbor area (Fig. 2C) were also modestly, but significantly, impaired; therefore, *Sema6a* is haploinsufficient for SAC dendrite morphology.

We next performed sparse *Sema6a* cKO using *Chat^CreER^* and *ROSA-LSL^tdTomat^*^o^ to label SACs. Targeted P14 *Sema6a* cKO ON- and OFF-SACs exhibited distal dendrite crossovers and reduced dendritic arbor area (Fig. 2G-I), demonstrating that isoneuronal repulsive Sema6A signaling cell-autonomously regulates these processes. Surprisingly, cell-autonomous removal of *Sema6a* did not alter SAC symmetry (Fig. 2G). Preservation of SAC radial morphology in SAC-cKO retinas at P42 confirmed that ON-SAC radial symmetry and plexus organization do not require Sema6A in SACs (Fig. S2D-F); only distal dendrite self-avoidance and arbor area were affected by cell-type autonomous loss of *Sema6a* in *Chat^Cre/+^; Sema6a^HA-F/−^* SACs. Therefore, large gaps in ON-SAC plexus organization (arrowheads in Fig. S2A,G; Sun et al., 2013) do not result from the distal dendrite self-avoidance defects that arise when SACs no longer express Sema6A.

In *C. elegans*, isoneuronal semaphorin-plexin signaling regulates the position of presynaptic specializations within both DA8 and DA9 motorneurons, which requires interaxonal interactions between co-fasciculated DA8 and DA9 axons (Mizumoto and Shen, 2013). SAC dendrites are highly co-fasciculated and their synaptic specializations are clearly regionalized; presynaptic specializations concentrate in the distal one-third of the SAC arbor (Briggman et al., 2011). To determine whether Sema6A–PlexA2 signaling contributes to SAC presynaptic specialization, we used *ROSA-LSL-Synaptophysin^tdTomato^* to Cre-dependently label presynapses while conditionally removing *Sema6a* within SACs using *Chat^CreER^*. In P14 *Sema6a^HA-F/LacZ^* cKO retinas, there was an apparent increase in presynaptic specializations in the inner and middle thirds of ON- and OFF-SAC arbors (Fig. S3A,B). Yet synapse localization was preserved in P21 SAC-specific (*Chat^CreER/+^*; *Sema6a^HA-F/−^*) cKO retinas (Fig. S3C,D), suggesting that our initial observations were specific to the presence of the *Sema6a^LacZ^* allele. While we cannot rule out resolution of synapse mislocalization by P21 in *Sema6a^HA-F/−^* cKO mice, synapse distribution was also preserved in P14 *Plxna2* mutant retinas (Fig. S3E,F), suggesting that Sema6a–PlexA2 signaling in SACs is dispensable for their presynaptic specialization.

HA-Sema6A and PlexA2 were enriched at branch points in all cultured SACs examined (Fig. S4), supporting isoneuronal Sema6A–PlexA2 signaling for self-avoidance of branching sister dendrites. Together, these data demonstrate a requirement for Sema6A in all SACs for distal dendrite self-avoidance and arbor elaboration, but not for arbor symmetry or presynaptic specialization, and suggest that perturbations in SAC symmetry and plexus organization in *Sema6a* mutants arise non-cell-autonomously.

### Sema6A is expressed by all SACs

**Fig. 3.**
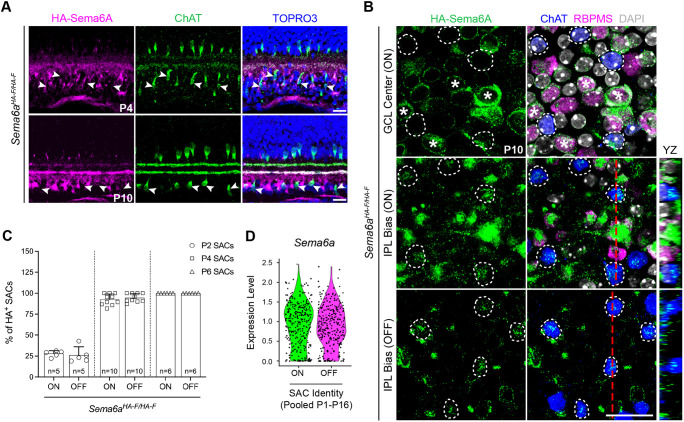
**Sema6A is expressed by both ON- and OFF-SACs.** (A) HA-Sema6A and ChAT immunolabeling in *Sema6a^HA-F/HA-F^* retinas (arrowheads, SAC somas). (B) HA-Sema6A is not evident in ChAT^+^ ON-SACs (dashed circles) in the GCL center (top) but is evident in the same SACs (dashed circles) imaged with a bias toward the IPL (middle). HA-Sema6A is detected in RBPMS^+^ RGCs (asterisks). HA-Sema6A is detected in OFF-SACs (bottom, white circles). Red dashed lines correspond to *yz* orthogonal views. (C) Quantification HA-Sema6A^+^ SACs across early postnatal development. (D) Violin plot of pooled SAC *Sema6a* expression from P1, P4, P8, P11 and P16. *n*, number of retinas. Scale bars: 20 µm.

HA-Sema6A expression was not obvious in ON-SACs of P4 and P10 retinal cross-sections (Fig. 3A, arrowheads), so we examined whole-mount retinas. ON-SACs imaged through the GCL center did not appear to express HA-Sema6A, while many RGCs labeled by the pan-RGC marker RBPMS (Rodriguez et al., 2014) were HA-Sema6A positive (Fig. 3B, top). Biasing our imaging plane toward the IPL revealed HA-Sema6A in ON-SACs (Fig. 3B, middle) that appeared to be devoid of HA-Sema6A when imaged through the GCL center (Fig. 3B, top). HA-Sema6A was also evident in OFF-SACs when imaged in a similar fashion (Fig. 3B, bottom). We characterized Sema6A expression across early postnatal development and found that only ∼25% of ON- and OFF-SACs expressed Sema6A at P2, close to when SAC dendrites laminate (Ray et al., 2018; Sun et al., 2013). Between P2 and P4, Sema6A in SACs increases; by P6, HA-Sema6A is detectable in all SACs (Fig. 3C). We previously observed an apparent lack of Sema6A expression in some SACs *in vitro* and in OFF-SACs *in vivo* (Sun et al., 2013), yet here we detect HA-Sema6A in all SACs. We attribute this to the unreliability of anti-Sema6A immunolabeling.

To investigate the timing and cell types that express *Sema6a*, we generated a *Sema6a^CreER^* knock-in allele (Fig. S5A; see Materials and Methods). *Sema6a^CreER^*-driven expression of *ROSA-LSL^tdTomato^* was evident in ∼20% of all SACs from P1-P3, while ∼70% of RGCs were tdTomato^+^ (Fig. S5B,C). *Sema6a* was also expressed in ∼36% of presumptive displaced non-SAC amacrine cells (arrows in Fig. S5B). *Sema6a^CreER^* reporter activity in SACs peaked at ∼40% by P8 and was nearly absent from P14 onward (Fig. S5D,E).

Since our expression analyses relied on transgenic animals, we confirmed these results by single-cell mRNA sequencing (scRNA-seq). P1, P4, P8, P11 and P16 *Chat^Cre/+^; ROSA-LSL^tdTomato/+^* SACs were profiled (see Materials and Methods). At each timepoint, except P16, SACs unbiasedly clustered into two groups (Fig. S6A). All clusters were enriched for SAC genes (Fig. S6B-E). *Sema6a* was expressed by all SACs, *Sema6a* and *Plxna2* levels were comparable across postnatal development, and both were significantly downregulated by P16 (Fig. 3D; Fig. S6F-I), consistent with minimal *Sema6a^CreER^* reporter activity at P14 (Fig. S5D).

Together, these data support the roles for Sema6A in both ON- and OFF-SAC dendrite elaboration revealed by our phenotypic characterizations. Furthermore, SAC dendritic stratification is complete by P1 (Ray et al., 2018), only ∼20% of SACs exhibit *Sema6a* reporter activity neonatally, and Sema6A is robustly expressed by other cell types in the neonatal retina (Fig. 3; Fig. S5). These three observations suggest that another cell type likely promotes Sema6A-mediated SAC dendrite scaffold segregation.

### Forward Sema6A–PlexA2 signaling directs SAC dendritic arbor elaboration

Sema6A can function as a ligand (forward signaling) via PlexA2 or as a receptor (reverse signaling; Battistini and Tamagnone, 2016). To assess reverse Sema6A signaling in SAC development, we used a *Sema6a^ΔCyt^* allele that allows Cre-dependent conditional removal of the Sema6A cytoplasmic domain (Verhagen et al., 2023 preprint). We examined organization of the ON-SAC dendrite plexus as a proxy for radial symmetry, as the ON-SAC plexus has gaps only when SAC radial symmetry is disrupted (note the uniformity in Fig. S2D versus Fig. S2A). Embryonic conditional removal of the Sema6A cytoplasmic domain in *Megf10C^re/+^; Sema6a^ΔCyt^* SACs did not alter SAC plexus uniformity (Fig. S7A,B) or dendrite lamination (Fig. S7C); therefore, Sema6A reverse signaling in SACs is dispensable for SAC dendritic arbor development.

PlexA2, a receptor for forward repulsive Sema6A signaling, is exclusively expressed in SACs in the neonatal retina and is required for SAC dendrite lamination and development (Matsuoka et al., 2011a; Sun et al., 2013). Forward signaling requires plexin cytoplasmic RasGAP activity (Pascoe et al., 2015). We used the *Plxna2^R1746A^* allele (Zhao et al., 2018; *Plxna2^ΔRasGAP^*), in which a point mutation abolishes PlexA2 RasGAP activity, to investigate whether PlexA2 forward signaling impacts SAC development. *Plxna2^+/−^* retinas rarely exhibit SAC strata crossovers, while *Plxna2^ΔRasGAP/−^* retinas exhibit significantly more SAC crossovers (Fig. S7D). This was only ∼27% of *Plxna2^−/−^* retina crossovers, suggesting that PlexA2 RasGAP forward signaling only partially contributes to SAC lamination (Fig. S7D).

**Fig. 4.**
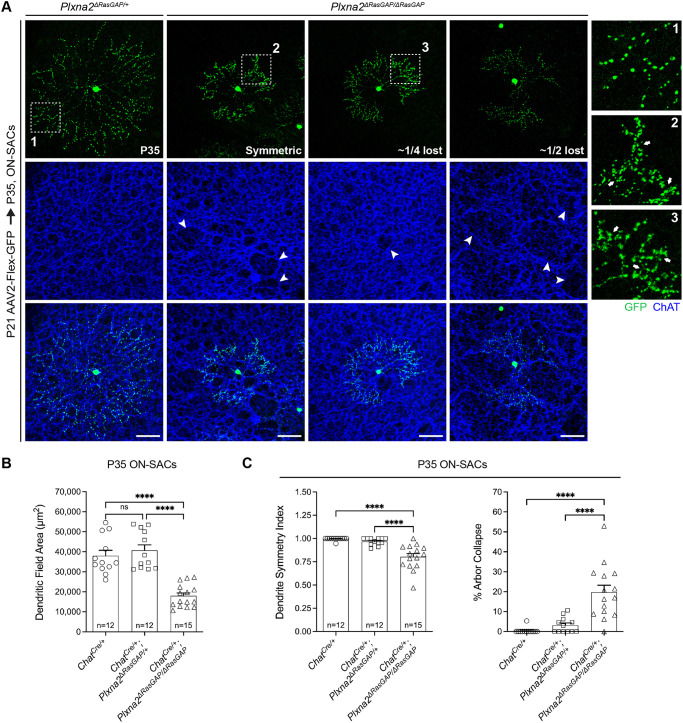
**Forward signaling through the PlexA2 RasGAP domain is required for SAC development.** (A) Representative ON-SACs. Varying degrees of radial symmetry are observed in *Chat^Cre/+^; Plxna2^ΔRasGAP/ΔRasGAP^* ON-SACs. Distal dendrites of every *Plxna2^ΔRasGAP/ΔRasGAP^* ON-SAC examined exhibited self-avoidance errors (arrows). The *Plxna2^ΔRasGAP/ΔRasGAP^* ON-SAC plexus contains large holes and gaps (arrowheads). (B,C) Quantification of P35 *AAV2-FLEX-GFP* labeled ON-SAC dendritic field area (B) and symmetry (C). *n*, number of SACs. ****P<0.0001; one-way ANOVA, Tukey's MCT. Scale bars: 50 µm.

We next examined SAC morphology in *Chat^Cre/+^; Plxna2^ΔRasGAP/ΔRasGAP^* retinas transduced with *AAV2-FLEX-GFP*. *Plxna2^ΔRasGAP/ΔRasGAP^* mutant ON-SAC dendrite arbor area and distal dendrite self-avoidance were severely compromised (Fig. 4A-C), and the ON-SAC plexus exhibited large gaps, further supporting the link between ON-SAC radial symmetry and plexus gaps (Fig. 4A, arrowheads). Therefore, forward PlexA2 signaling is required for ON-SAC dendrite arbor area, symmetry and self-avoidance. Few OFF-SACs were sufficiently labeled to permit quantification of OFF-SAC dendritic arbor morphology, but those that were imageable exhibited mostly radial dendritic arbors with distal dendrite self-crossovers (Fig. S7E), strongly suggesting that forward signaling through PlexA2 is required for self-avoidance in all SACs. Large gaps in the OFF-SAC dendritic plexus were not detected in *Plxna2^ΔRasGAP/ΔRasGAP^* mutants (Fig. S7F).

### Sema6A is broadly required for inner retina development

**Fig. 5.**
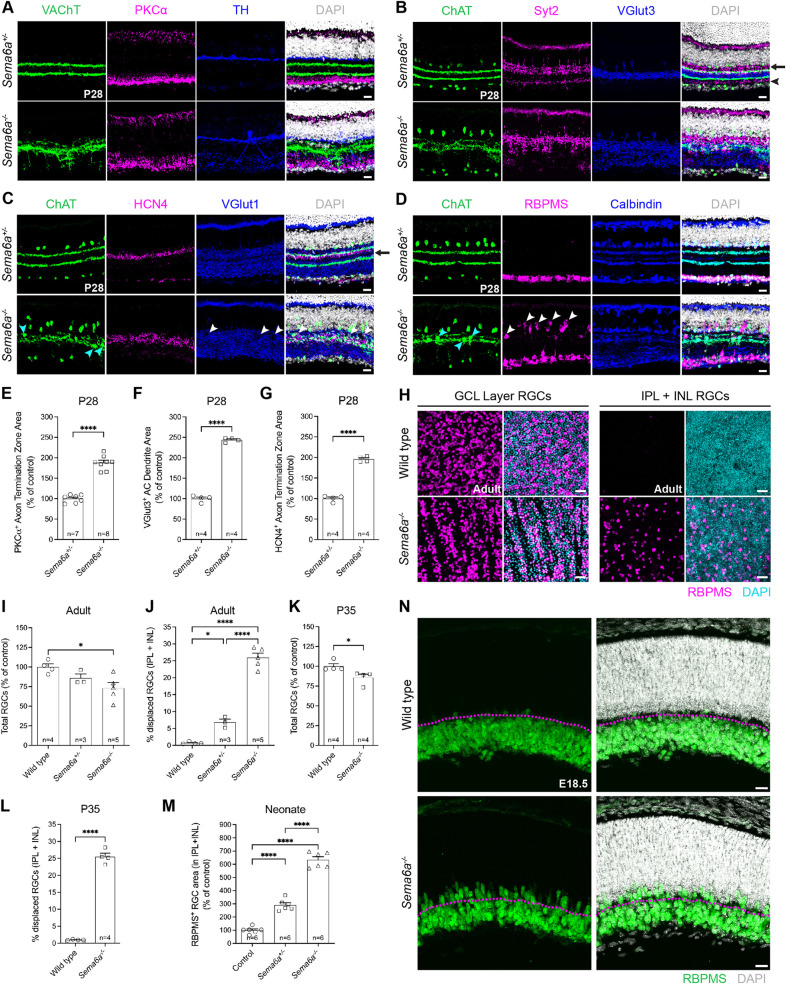
***Sema6a* is broadly required for inner retina development.** (A-D) P28 retinas labeled with: anti-VAChT (SACs), anti-PKCɑ (rod BCs) and anti-tyrosine hydroxylase (TH^+^ ACs) (A); anti-ChAT (SACs), anti-Syt2 (type 2 OFF CBCs, see arrow; type 6 ON CBCs, see arrowhead) and anti-VGlut3 (VGlut3^+^ ACs) (B); anti-ChAT, anti-HCN4 (type 3a BCs) and anti-VGlut1 (BC IPL axon terminals) (C); and anti-ChAT, anti-RBPMS (RGCs) and anti-calbindin (subtypes of RGCs and ACs) (D). (E) Quantification of PKCɑ axon terminal zone expansion. (F) Quantification of VGlut3^+^ AC dendrite mis-stratification. (G) Quantification of HCN4+ axon terminal zone expansion. (H) *Z*-projections of whole-mount retinas. (I,K) Quantification of total RGCs. (J,L) Quantification of IPL+INL displaced RGCs. (M) Quantification of displaced neonatal RGC RBPMS^+^ immunoreactive area. (N) Representative images of RBPMS^+^ RGC mislocalization quantified in M, apical to the magenta dashed line. Wild type, C57BL/6. *n*, number of retinas. *P<0.05; ****P<0.0001; Student's *t*-test or one-way ANOVA with Tukey's MCT. Scale bars: 20 µm in A-D,N; 40 µm in H.

SAC dendrite plexuses serve as scaffolds that direct lamination of incoming retinal neurites (Peng et al., 2017; Duan et al., 2018). Given the severity of *Sema6a^−/−^* SAC lamination defects, we examined inner retinal phenotypes. As expected, the vesicular acetylcholine transporter-positive (VAChT) SAC strata were fused in *Sema6a^−/−^* retinas, and, consistent with our previous findings (Matsuoka et al., 2011a), tyrosine hydroxylase (TH)-expressing ACs dendrites mis-projected into ON IPL layers (Fig. 5A). VGlut3, which labels a population of ACs (Fremeau et al., 2002) stratifying neatly between the S2 and S4 SAC layers, was no longer confined in *Sema6a^−/−^* retinas (Fig. 5B,F).

We also noted expansions of the PKCα^+^ rod bipolar cell (RBC; Haverkamp et al., 2003) (Fig. 5A,E), OFF type 2 cone bipolar cell (CBC) (Fig. 5B: arrow, strong synaptotagmin-2 (Syt2) labeling in S1 and S2) and ON type 6 CBC (Fig. 5B: arrowhead, weak Syt2^+^ signal in S5; Fox and Sanes, 2007; Wässle et al., 2009) axon termination zones in *Sema6a^−/−^* retinas. The HCN4^+^ OFF type 3a CBC (Müller et al., 2003) axon termination zone, which usually terminates in a tight band immediately basal to S2, was also expanded (Fig. 5C,G). The HCN4^+^ band is normally immediately apical to the middle of S3, a region with sparse BC axon terminations revealed by vesicular glutamate transporter 1 (VGlut1) (Johnson et al., 2003). This likely represents a boundary between OFF and ON IPL circuits (Fig. 5C, black arrow). In *Sema6a^−/−^* retinas, the BC axon terminal-free (VGlut1^−^) boundary is no longer evident, and frequent holes, corresponding to displaced cell bodies, disrupt VGlut1 immunolabeling (Fig. 5C, white arrowheads). Together, these results suggest a blurring of the boundary separating specialized OFF versus ON domains of the *Sema6a^−/−^* IPL.

Finally, we examined RGC and AC lamination patterns. As in *Sema6a^LacZ/LacZ^* retinas (Matsuoka et al., 2011a), calbindin^+^ neurites basally mis-project in *Sema6a^−/−^* retinas (Fig. 5D). RBPMS^+^ RGC GCL localization was disrupted in *Sema6a^−/−^* retinas (Fig. 5D, white arrowheads), and SACs were often displaced into the IPL (Fig. 5C,D, cyan arrowheads). ON-SACs in the GCL were reduced by ∼45%, and ∼10% of OFF-SACs were missing from the INL of *Sema6a^−/−^* retinas (Fig. S8A,B), which could reflect SAC displacement into the IPL, cell death or a combination of these possibilities. We next assessed apicobasal RGC positioning in whole-mount retinas; RBPMS^+^ RGCs in the IPL and INL were considered ‘displaced’. In adult (>P56) *Sema6a^−/−^* retinas, we observed a 26% loss of total RBPMS^+^ RGCs and a 25% increase in displaced RGCs (Fig. 5H-J). We examined P35 juvenile *Sema6a^−/−^* retinas and observed a loss of ∼15% of total RGCs, indicating that RGC loss increases over time (Fig. 5K). P35 mutant retinas also exhibited a 25% increase in displaced RGCs (Fig. 5L), suggesting that RGC mislocalization remains constant and precedes RGC loss. A marked increase in RGCs apical to the inner neuroblastic layer (INBL) was observed in neonatal (E18.5-P0.5) *Sema6a^−/−^* retinas (Fig. 5M,N). Thus, RGCs are mislocalized early in development, and postnatal RGC loss increases over time, likely reflecting axon targeting defects and, subsequently, a lack of trophic support.

These results show that, in the complete absence of Sema6A, BCs, ACs and RGCs exhibit a broad range of targeting defects. Deficiencies in cell migration and neurite lamination of various retinal cell types suggest distinctions between early and late roles for Sema6A in retinal development.

### Sema6A–Plexin-dependent and -independent mechanisms direct retinal development

PlexA1-A4 are expressed in the developing retina, yet only PlexA2 and PlexA4 are retinal Sema6A receptors (Matsuoka et al., 2011a,b; Sun et al., 2013). *Plxna4* is not absolutely required for SAC lamination (Matsuoka et al., 2011a), but plexin redundancy could mask PlexA4 contributions to this process. We analyzed retinal phenotypes in *Plxna2^−/−^; Plxna4^−/−^* retinas, and SAC lamination defects were no more severe than in *Plxna2^−/−^* retinas (Fig. S8C,F; Sun et al., 2013). The RBC axon termination zone in *Plxna2^−/−^; Plxna4^−/−^* retinas was maintained and, as expected (Matsuoka et al., 2011a), TH^+^ AC dendrites mis-projected into the inner IPL. Thus, the severity of SAC lamination defects in *Sema6a^−/−^* retinas is plexin independent.

Like *Sema6a^−/−^* retinas, in *Plxna2^−/−^; Plxna4^−/−^* double mutants, but not in *Plxna2* or *Plxna4* single mutants (data not shown), Syt2^+^ OFF type 2 CBC, HCN4^+^ OFF type 3a CBC and VGlut1^+^ BC axon terminations were disrupted (Fig. S8D,E), supporting plexin receptor redundancy in guiding BC axons. Targeting of RBC (PKCα^+^; Fig. S8C) and type 6 CBC (arrowheads in Fig. S8D) axons, and VGlut3^+^ AC dendrites (Fig. S8D) was preserved in *Plxna2^−/−^; Plxna4^−/−^* retinas, and mild RGC mislocalization was observed (Fig. S8F). RGC displacement was comparable between *Plxna2^−/−^; Plxna4^−/−^* and *Plxna2^−/−^; Plxna4^+/−^* mutants, but was not impacted in *Plxna2^+/−^; Plxna4^−/−^* mutants (Fig. S8F). Therefore, we quantified RGC displacement in *Plxna2^−/−^* whole-mount retinas and found that total RGC number was unchanged and only 6% of RGCs were displaced (Fig. S8G-I), indicating that RGC displacement and loss in *Sema6a^−/−^* retinas is largely plexin independent.

Together, our examination of retinal neurite lamination patterns reveals unique mechanisms underlying IPL targeting: some cell types, including VGlut3^+^ ACs, RBCs and ON type 6 CBCs, do not require PlexA receptors, and PlexA2 is only minimally required for RGC localization. Neurite mistargeting and RGC localization phenotypes may arise through PlexA-independent Sema6A signaling, but we favor the hypothesis that most deficits arise due to the apicobasal disruption of the SAC scaffolds in *Sema6a^−/−^* retinas. Other cell types, including SACs and OFF type 2 and OFF type 3a CBCs, require Sema6A–PlexA interactions for neurite lamination.

### Sema6A is expressed by ON and ON-OFF DSGCs

**Fig. 6.**
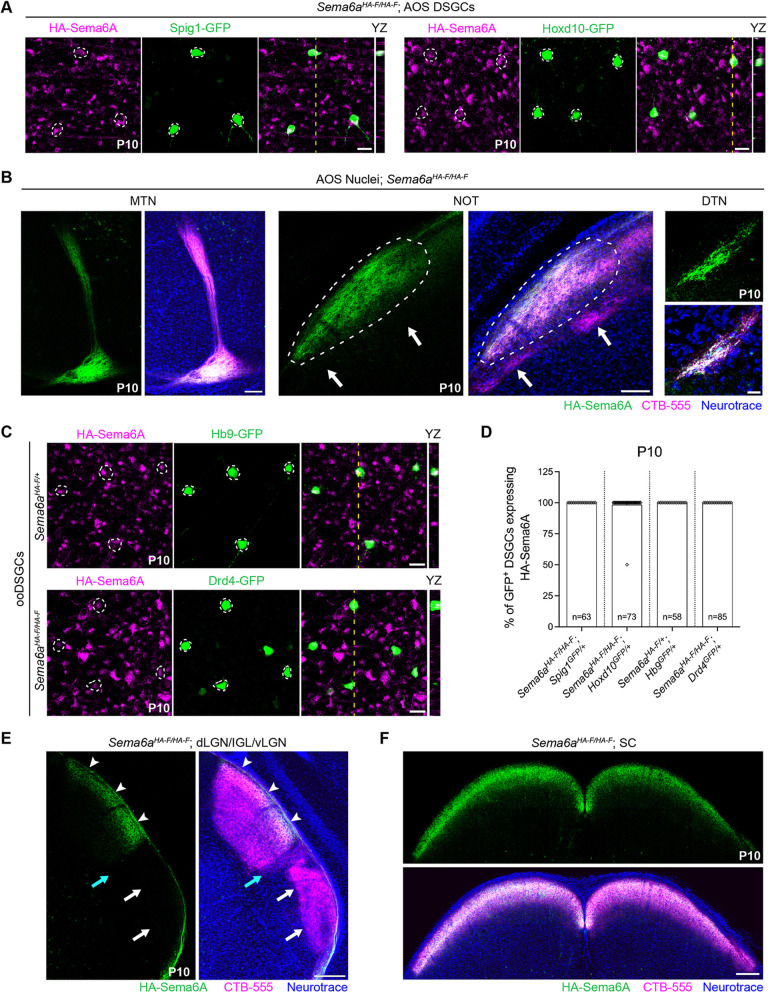
**Sema6A is expressed by ON and ON-OFF DSGCs.** (A) Spig1-GFP^+^ oDSGCs (left) and Hoxd10-GFP^+^ DSGCs (right) express HA-Sema6A. Scale bars: 20 µm. (B) HA-Sema6A in AOS midbrain nuclei, including the MTN, NOT (white dashed line) and DTN. HA-Sema6A is not detected in the olivary pretectal nucleus (white arrows). Scale bars: 100 µm in MTN and NOT; 20 µm in DTN. (C) HB9-GFP^+^ and Drd4-GFP^+^ ooDSGCs express HA-Sema6A. Scale bars: 20 µm. (D) Quantification of the percentage of DSGCs expressing HA-Sema6A per imaged region of interest. (E) HA-Sema6A in the ooDSGC-targeted shell of the dLGN (white arrowheads). The dLGN core, inner geniculate leaflet (cyan arrow) and ventral LGN (white arrows) lack HA-Sema6A. Scale bar: 200 µm. (F) HA-Sema6A expression in superficial ooDSGC target layers of the SC. Scale bar: 200 µm. CTB-555 was binocularly injected at P8 to label retinorecipient nuclei (B,E,F). Dashed circles in A and C outline GFP+ RGC somas; yellow dashed lines represent the *yz* orthogonal presentation (right). *n*, number of cells.

Sema6A in SACs is dispensable for SAC lamination (Fig. 1). What is the source of Sema6A that drives SAC scaffold segregation? Sema6A is robustly expressed by RGCs (Fig. 3 and Fig. S5), and subsets of DSGCs have immature dendritic arbors in the IPL at P1 positioned to interact with SACs (Peng et al., 2017). We have previously observed anti-Sema6A immunolabeling of oDSGCs but not ooDSGCs (Sun et al., 2015). HA-Sema6A is expressed in Spig1-GFP^+^ and Hoxd10-GFP^+^ DSGCs (Fig. 6A) of the AOS and their retinorecipient midbrain nuclei, including the medial terminal nucleus (MTN), nucleus of the optic tract (NOT) and dorsal terminal nucleus (DTN) (Fig. 6B; Dhande et al., 2013; Yonehara et al., 2008). We also detected HA-Sema6A in Hb9-GFP^+^ ventral and Drd4-GFP^+^ nasal motion-preferring ooDSGCs (Fig. 6C,D) that project to the shell of the dorsal lateral geniculate nucleus (dLGN) and the outer shell of the superior colliculus (SC) (Zhang et al., 2017). HA-Sema6A was detected within these ooDSGC retinorecipient targets in *Sema6a^HA-F/HA-F^* mice (Fig. 6E,F). To confirm that RGC axon terminals in these image-forming retinorecipient targets express HA-Sema6A, we performed enucleation experiments. HA-Sema6A in the dLGN shell and outer SC disappeared 1 week after unilateral enucleation (Fig. S9A,B). HA-Sema6A was not expressed by RGC axons in non-image forming retinorecipient nuclei (Fig. S9C). These results show that both DSGC subtypes synaptically connected to SACs, oDSGCs and ooDSGCs, express Sema6A, raising the possibility that Sema6A in DSGCs segregates the SAC scaffolds.

### Sema6A in RGCs influences ON-SAC plexus elaboration and RGC migration

**Fig. 7.**
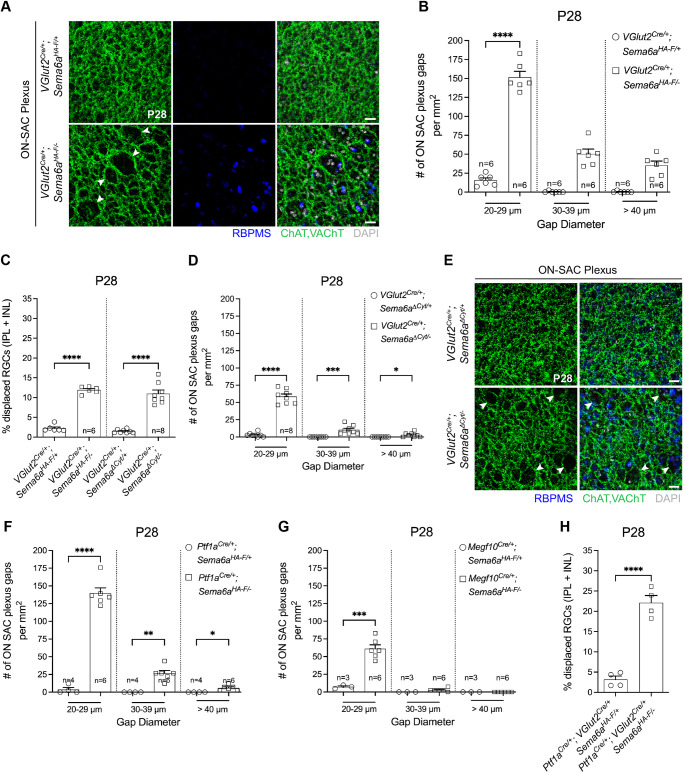
**Sema6A is cell-autonomously required in RGCs for their migration and for ON-SAC plexus organization.** (A,B) Large gaps (white arrowheads) (A) and quantification of gaps (B) in the ON-SAC dendritic plexus of *VGlut2^Cre/+^; Sema6a^HA-F/−^* cKO retinas. (C) Quantification of displaced RGCs. (D,E) Quantification (D) and representative images (E) of ON-SAC plexus gaps (white arrowheads) in *VGlut2^Cre/+^; Sema6a^ΔCyt/−^* cKO retinas. (F) Quantification of ON-SAC plexus gaps in pan-AC (*Ptf1a^Cre^*) cKO retinas. (G) Quantification of ON-SAC plexus gaps in SAC-specific cKO retinas. (H) Quantification of displaced RGCs in pan-AC (*Ptf1a^Cre^*) and pan-RGC (*VGlut2^Cre^*) double cKO retinas. *n*, number of retinas. *P<0.05; **P<0.01; ***P<0.001; ****P<0.0001; one-way ANOVA, Tukey's MCT (B-D,F,G); Student's *t*-test (H). Scale bars: 50 µm.

At E14.5, *Sema6A* mRNA is predominantly expressed by presumptive postmitotic RGCs (Fig. S10A), which are basally positioned in the embryonic INBL (Ray et al., 2018) before SAC migration into the INBL (Peng et al., 2020). Thus, RGC-derived Sema6A is situated to influence early retinal patterning. We used *VGlut2^Cre^* to conditionally remove *Sema6a* from embryonic, newly postmitotic RGCs (Wilkinson et al., 2021) in *VGlut2^Cre/+^*; *Sema6a^HA-F/−^* retinas (Fig. S10B). We found that ON-SAC dendritic plexus organization is compromised in these RGC-cKO retinas (Fig. 7A,B). The ON plexus exhibited large gaps reminiscent of *Sema6a^LacZ^* and *Plxna2* mutant retinas (Fig. S2G and Fig. 4A, arrowheads). We measured gap diameter (see Materials and Methods) and found that *VGlut2^Cre^* RGC-cKO retinas had ∼150 small (20-29 µm diameter) gaps per mm^2^, while controls had only 15 (Fig. 7B). Control ON-SAC plexuses rarely exhibited gaps greater than 30 µm, while RGC-cKO retinas had ∼50 intermediate (30-39 µm) and ∼35 large (>40 µm) gaps per mm^2^. RBPMS^+^ RGCs were often observed within these gaps, clumped together with other RBPMS^–^ cells (Fig. 7A). The total number of RGCs was not impacted (Fig. S11A), demonstrating that RGC-derived Sema6A is not required for RGC survival (Fig. 5I,K). Despite displacement of many RBPMS^+^ RGCs within S2, OFF-SAC plexus organization was not altered in RGC-cKO retinas (Fig. S11B). Only 12% of RGCs were displaced in RGC-cKO retinas, approximately half of what was observed in *Sema6a^−/−^* mutants (Figs 7C and 5J,L).

We next asked whether reverse Sema6A signaling influences SAC plexus elaboration. Large ON-SAC plexus gaps were detected in *VGlut2^Cre/+^*; *Sema6a^ΔCyt/−^* retinas, but the phenotype was less severe than in *VGlut2^Cre/+^; Sema6a^HA-F/−^* cKO retinas (Fig. 7D,E versus 7A,B). However, RBPMS^+^ RGCs were comparably displaced in *VGlut2^Cre/+^*; *Sema6a^ΔCyt/−^* and full-length cKO retinas (Fig. 7C, Fig. S11C). The total number of RGCs was also unchanged in *VGlut2^Cre/+^*; *Sema6a^ΔCyt/−^* retinas (Fig. S11A). Therefore, Sema6A reverse signaling within RGCs contributes to RGC settling in the developing inner retina and is partially required for ON-SAC dendrite plexus organization, a process likely influenced by displaced cell bodies and/or neurites within the plexus gaps. However, loss of reverse Sema6A signaling does not fully account for the ON-SAC plexus gap phenotype of full-length *Sema6a* RGC-cKO retinas, indicating that both Sema6A forward and reverse signaling events impact ON-SAC plexus organization.

Since 12% of RGCs are displaced in *VGlut2^Cre^* cKO but 25% are displaced in *Sema6a^−/−^* retinas, we investigated whether other cell types are partially responsible for RGC displacement. We conditionally removed *Sema6a* from all postmitotic ACs using *Ptf1a^Cre^* (Fig. S11D; Fujitani et al., 2006), from all embryonic SACs (*Megf10C^re^*) or in retinas where the cytoplasmic domain of Sema6A was removed from all embryonic SACs (*Megf10C^re/+^*; *Sema6a^ΔCyt/−^*). In each of these cKO backgrounds, total RGC number was unaltered and only ∼7% of RGCs were displaced (Fig. S11E,F), the same fraction as in *Sema6a^+/−^* retinas (Fig. 5J), indicating that RGC displacement in these AC-cKO backgrounds stems from *Sema6a* haploinsufficiency. However, RGC displacement was significantly greater in RGC-cKO retinas compared to *Sema6a^+/−^* retinas (Fig. S11G), implicating a specific role for RGC-derived Sema6A in RGC positioning.

We next assessed ON-SAC plexus organization in *Ptf1a^Cre^* AC-cKO retinas. The number of small gaps (20-29 µm) was comparable between AC- and full-length RGC-cKO retinas, but the number of intermediate and large gaps was greater in full-length RGC-cKO retinas (Fig. 7B,F). In SAC-cKO (*Megf10C^re^*) retinas, only the number of small gaps was altered (Fig. 7G). Small gaps in SAC-cKO retinas were ∼44% of pan-AC-cKO (*Ptf1a^Cre^*) retinas, demonstrating that non-SAC ACs impact ON-SAC plexus organization. Together, these results suggest that approximately half of the small gaps in the ON-SAC plexus of *Sema6a* mutants arise SAC-cell-autonomously, while the other half and intermediate and large gaps arise SAC-non-autonomously. Unlike *Sema6a^−/−^* retinas (Fig. S8A,B), SAC somas were not displaced in RGC- or AC-cKO retinas (Fig. S11H,I).

We next generated *VGlut2^Cre^* and *Ptf1a^Cre^* double transgenic mice to conditionally remove *Sema6a* from both embryonic RGCs and ACs. Total RGC number was unaltered in *Ptf1a^Cre^; VGlut2^Cre^* cKO retinas (Fig. S11J), but ∼22% of RGCs were displaced (Fig. 7H). Since loss of Sema6A in ACs was not sufficient to displace RGCs, these data suggest that ACs do not normally regulate RGC positioning. Reverse signaling through RGC-derived Sema6A normally contributes to RGC settling, and, in its absence, AC-derived Sema6A partially compensates to preserve localization of some RGCs through an unknown mechanism. Neither RGC- nor AC-derived Sema6A contributes to the progressive RGC loss observed from P35 to adulthood (Fig. S11A,E; Fig. 5I,K). Similarly, OFF-SAC INL positioning was preserved, while ∼22% of GCL-localized ON-SACs were missing, in *Ptf1a^Cre^; VGlut2^Cre^* double cKO retinas (Fig. S11H,I). This suggests that some ON-SACs are displaced into the IPL (Fig. 5) due to loss of inner retinal Sema6A, while others, and some OFF-SACs, do not survive (Fig. S8A,B) the RGC survival deficit that arises in *Sema6a^−/−^* retinas, the latter likely due to loss of Sema6A elsewhere along the visual pathway.

### Sema6A in RGCs promotes SAC dendrite stratification

**Fig. 8.**
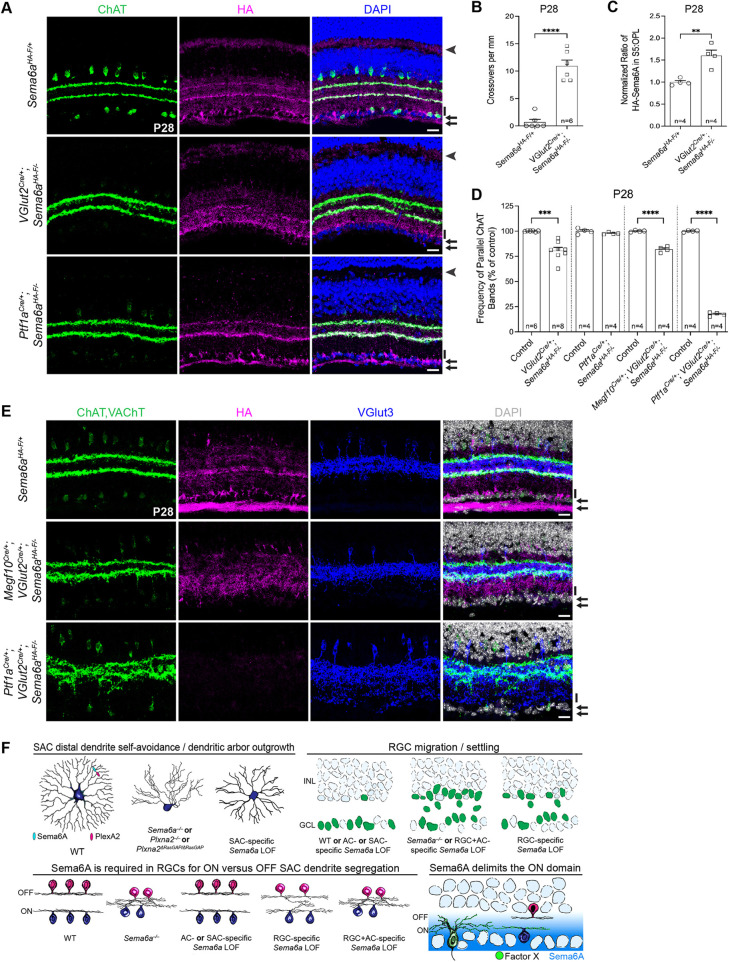
**RGC-derived Sema6A segregates SAC dendrites and defines the ON domain of the IPL.** (A) Anti-ChAT (SACs), anti-HA (Sema6A) and DAPI in retina cross-sections. Arrows indicate the GCL and nerve fiber layer (NFL). Arrowheads indicate the OPL; *Ptf1a^Cre^* recombines in horizontal cells (Fujitani et al., 2006), and correspondingly, HA-Sema6A is lost in the *Ptf1a^Cre^* cKO OPL. Black vertical bars indicate region of S5 scored in C. (B) IPL crossover quantification. (C) Quantification of upregulated HA-Sema6A in S5. (D) Quantification of parallel ChAT bands for the indicated genotypes. (E) Cross-sections labeled with anti-ChAT (SACs), anti-HA (Sema6A), anti-VGlut3 (VGlut3^+^ ACs) and DAPI. Arrows indicate HA-Sema6A in the GCL and NFL. Black vertical bars indicate a region of S5 with elevated HA-Sema6A. (F) Model of the roles of Sema6A in inner retinal development. Top left: isoneuronal Sema6A–PlexA2 signaling in SACs promotes distal dendrite self-avoidance and dendritic arbor morphology. Top right: RGC-derived Sema6A promotes RGC localization. Bottom left: RGC-derived Sema6A promotes SAC dendrite segregation. Bottom right: the proposed gradient of Sema6A provided by basally biased RGC dendrites repels (pushes) SAC dendritic arbors away from the GCL, while unknown factor(s) X basally fixes (pulls) the ON-SAC arbor relative to OFF-SACs. *n*, number of retinas. **P<0.01; ***P<0.001; ****P<0.0001; Student's *t*-test (B,C); one-way ANOVA with Tukey's MCT (D). Scale bars: 20 µm.

Sema6A in RGCs contributes to ON-SAC plexus organization; does it also regulate SAC IPL lamination? We assessed SAC lamination in P10 *VGlut2^Cre/+^; Sema6a^HA-F/−^* retinas, which exhibit a select loss of HA-Sema6A in RGCs (Fig. S12A). We detected crossovers between the SAC dendrite scaffolds (Fig. S12A-C), but the expressivity of this phenotype was not comparable to *Sema6a^−/−^* lamination defects (Fig. 5A-D). We next examined *VGlut2^Cre^* cKO retinas at P28 and, like P10, only infrequent crossovers between the SAC scaffolds were detected (Fig. 8A,B). Approximately 30% of crossovers were associated with DAPI^+^ nuclei in P28 *VGlut2^Cre^* cKO retinas, suggesting that displaced somas are not the primary driver of these SAC crossovers (Fig. S12D). Displaced retinal neurites, however, as observed in *Sema6a^−/−^* retinas (Fig. 5), are a likely candidate underlying SAC lamination deficits. We examined DSGCs in RGC-cKO retinas. oDSGC (Spig1-GFP) and ooDSGC (Drd4-GFP) dendrite lamination was unaltered (Fig. S12E,F), demonstrating that RGC-derived Sema6A does not regulate DSGC dendrite targeting. ooDSGC (Hb9-GFP) dendrite lamination in AC-cKO retinas was similarly preserved (Fig. S12G). Approximately 13% of ooDSGCs were displaced in RGC-cKO retinas, but not in AC-cKO retinas (Fig. S12H). These data reveal that RGC-derived Sema6A contributes to SAC, but not DSGC, dendrite lamination; the precise RGC subtype influencing SAC scaffold segregation remains obscured.

At P10 (Fig. S12A,I) and P28 (Fig. 8A,C), we observed a significant increase in HA-Sema6A in S5 of the IPL in RGC-cKO retinas. Both neonatal non-SAC ACs and RGCs express Sema6A (Fig. S5C), consistent with their ability to impact SAC development. AC-cKO did not alter SAC dendrite lamination (Fig. 8A,D), suggesting that AC-derived Sema6A does not normally segregate the SAC scaffolds. Removing *Sema6a* from all ACs highlights HA-Sema6A expression in DSGC dendrites (Fig. 8A, bottom), showing that broad Sema6A expression in neonatal RGCs refines to DSGCs as development proceeds, consistent with our *Sema6a^CreER^* expression analyses (Fig. S5). Indeed, *Sema6a^CreER^* reporter activity refines to DSGCs in aged mice (Hunyara et al., 2022). Increased S5 Sema6A in RGC-cKO retinas suggests that non-SAC ACs upregulate Sema6A in a compensatory fashion to preserve retinal patterning, supporting our observation that ACs partially maintain RGC localization in RGC-cKO retinas (Fig. 7). To investigate whether AC-derived Sema6A precludes detection of the *Sema6a^−/−^* SAC dendrite fusion phenotype in *VGlut2^Cre^* cKO retinas, we removed *Sema6a* from embryonic SACs and RGCs using *Megf10C^re^* and *VGlut2^Cre^*. We found that OFF- and ON-SACs were mostly segregated, as observed in *VGlut2^Cre^* cKO retinas, revealing that non-SAC AC-derived Sema6A is sufficient for SAC scaffold segregation (Fig. 8D,E).

Finally, we conditionally removed *Sema6a* from all ACs and RGCs using *Ptf1a^Cre^* and *VGlut2^Cre^*. HA-Sema6A was entirely lost in the inner retina and SAC scaffolds were essentially fused, as in *Sema6a^−/−^* retinas (Fig. 8D,E, bottom). Additionally, while VGlut3^+^ AC neurites are properly laminated in the single cKO backgrounds examined, including *Ptf1a^Cre^* (Fig. S12J), *VGlut2^Cre^* (data not shown) and in *Megf10C^re^; VGlut2^Cre^* cKO retinas (Fig. 8E, middle), they mis-project in contexts where SAC dendrites are fused (Fig. 8E, bottom; Fig. 5B). This demonstrates that, for some cell types, disruptions in IPL neurite lamination are a secondary consequence of the loss of discrete SAC scaffolds, rather than a direct consequence of aberrant Sema6A signaling.

Together, these results show that Sema6A in RGCs: (1) prevents ON-SAC plexus disorganization; (2) prevents RGC and other non-RBPMS^+^ cell displacement into the IPL; and (3) facilitates SAC dendrite scaffold segregation. Therefore, SACs rely on Sema6A expressed in RGCs for their dendritic arbor development in both planes – apicobasal and *en face* – and RGC-dependent elaboration of the SAC scaffolds is important for retinal patterning.

## DISCUSSION

SAC dendrites carve out the nascent IPL and segregate into distinct OFF/ON layers by P1 (Ray et al., 2018). SACs instruct DS circuit formation by forming scaffolds upon which DSGCs form synaptic connections (Peng et al., 2017; Duan et al., 2018). Here, we conducted *Sema6a* cKO experiments to investigate how cell type-specific Sema6A signaling events impact SAC scaffold elaboration. We predicted that SAC-specific *Sema6a* loss of function would phenocopy *Sema6a^LacZ^* loss of function and confirm our hypothesis that Sema6A in ON-SACs repels OFF-SACs (Sun et al., 2013). We instead discovered a crucial role for RGC-derived Sema6A in SAC scaffold elaboration.

### Sema6A–PlexA2 signaling in all SACs regulates dendritic arbor development

From SAC-specific *Sema6a* cKO retinas we learned that SAC-derived Sema6A is dispensable for SAC dendrite lamination, a critical first step in DS circuit formation (Peng et al., 2017; Duan et al., 2018). Examination of ON-SAC morphology in *Sema6a^−/−^*, SAC-cKO, and *Plxna2^ΔRasGAP/ΔRasGAP^* retinas revealed that forward isoneuronal Sema6A–PlexA2 signaling is essential for distal dendrite self-avoidance and dendritic arbor area (Fig. 8F). OFF-SAC dendrite arbors were similarly perturbed in the various mutant backgrounds we examined, consistent with decreased OFF-SAC dendritic arbor area in *Plxna2^−/−^* retinas (Sun et al., 2013). Our multimodal expression analyses revealed *Sema6a* expression in all SACs, corroborating the OFF-SAC dendritic arbor phenotypes we observed. These results demonstrate that forward isoneuronal Sema6A–PlexA2 signaling in all SACs promotes distal dendrite self-avoidance, underscoring complementarity between distinct dendritic self-avoidance mechanisms: protocadherin signaling is crucial for SAC primary dendrite self-avoidance (Lefebvre et al., 2012) and Sema6A–PlexA2 signaling promotes distal dendrite self-avoidance (Sun et al., 2013; this study).

SAC symmetry and overall plexus organization were preserved in SAC-specific *Sema6a* cKO retinas, which exhibit SAC distal dendrite self-avoidance errors, distinguishing between plexus gaps that originate cell-autonomously through impaired self-avoidance, and gaps likely to reflect SAC dendrite collapse in response to atypical non-cell-autonomous repulsive interactions. Large gaps in RGC-cKO retinas, where RGC position is disrupted, suggest that previously unreported repulsive interactions from displaced RGCs or their neurites disrupt the ON-SAC plexus. SAC dendrites collapse *in vitro* if they contact other cell types (Fig. S13), suggesting sensitivity of SAC arbor symmetry to non-SAC interactions. Additionally, pan-AC-cKO introduces more ON-SAC plexus gaps than SAC-cKO, indicating that non-SAC ACs regulate SAC dendrite development.

In *VGlut2^Cre^* cKO retinas, many RGCs are displaced into the OFF IPL layers and the basal-most INL, yet overall OFF-SAC plexus organization is undisturbed. ON-SAC plexus disorganization may be a secondary consequence of exposure to repulsive signaling that ON-SACs are selectively competent to respond to but would otherwise be isolated from under normal conditions. Alternatively, displacement of certain RGC subtypes or neurites into only the ON IPL layers may preclude OFF-SAC exposure to repulsive cues. Still, it is possible that *Sema6a* loss of function in a subset of RGCs removes a positive signaling pathway that promotes ON-SAC dendrite outgrowth or symmetry *en face*.

### Sema6A is required for RGC migration and settling in the GCL

Sema6A–PlexA2 signaling regulates retinal progenitor interkinetic nuclear migration (Belle et al., 2016). We show here that Sema6A is required for final positioning of some RGCs. RGC migration in zebrafish occurs in two phases: rapid radial soma translocation into the basal retina, followed by slower fine-positioning related to mosaic spacing. When basal RGC migration is impaired, RGCs stall near the apical retina surface (Icha et al., 2016). Here, basal RGC translocation was apparently preserved: RGCs were never found in the outer retina of *Sema6a* mutants. In neonatal *Sema6a^−/−^* retinas, RGCs accumulate at the outer neuroblastic layer/IPL boundary, suggesting that positioning defects in *Sema6a^−/−^* retinas do not reflect impaired basal RGC translocation. We propose that Sema6A instead contributes to RGC fine-positioning, and that in the presence of a previously unreported inhibitory boundary, loss of the attractive Sema6A signaling that normally facilitates RGC migration through the IPL, or a combination of both, blocks the final migration steps for ∼25% of *Sema6a^−/−^* RGCs.

We reasoned the displaced RGCs might consist of ooDSGCs, since ∼25% of total RGCs were displaced in *Sema6a^−/−^* retinas and ooDSGCs represent ∼29% of total RGCs (Chen et al., 2014). However, few ooDSGCs (13%) were displaced in *VGlut2^Cre^* cKO retinas, suggesting that displaced RGCs are likely not enriched for a single or a few subtypes, such as DSGCs. Notably, defective Sema6A–PlexA2 signaling impairs migration of later-born cerebellar granule neurons (Renaud and Chédotal, 2014). We propose that Sema6A promotes fine-scale RGC settling, specifically affecting later-born RGCs.

### Sema6A in RGCs delimits the ON domain of the IPL

Establishment of distinct OFF versus ON IPL domains is a key developmental event for patterning retinal circuits (Zhang et al., 2017). After segregation of OFF- and ON-SAC dendritic scaffolds, the inner retina is delineated from the outer retina by repulsive Sema5A and Sema5B signaling through PlexA1 and PlexA3 receptors (Matsuoka et al., 2011b). Here, we find that Sema6A expressed by postmitotic RGCs, the earliest-born retinal neuron subtype, initiates IPL circuit patterning by influencing SAC scaffold segregation. The new *Sema6a* null allele generated with our *Sema6a^HA−F^* cKO allele revealed a greater deficit in SAC scaffold lamination, consisting of near fusion of the ON- and OFF-SAC strata, than previously observed in *Sema6a^LacZ/LacZ^* retinas (Sun et al., 2013). Coupled with our observations of increased dendrite morphology deficits in *Sema6a^−/−^* ON-SACs and aberrant OFF-SAC dendrite morphology, which were not detected in *Sema6a^LacZ/LacZ^* retinas (Sun et al., 2013), we conclude that *Sema6a^LacZ^* is hypomorphic. Our observation of apparent synaptic localization defects in a background bearing the *Sema6a^LacZ^* allele, but not the bona fide null allele, supports the ability of *Sema6a^LacZ^* to drive neomorphic phenotypes, suggesting caution when using *Sema6a^LacZ^* and other gene-trap mice, as previously noted for *netrin-1^LacZ^* hypomorphic gene trap mice (Bin et al., 2015).

We observed modest SAC crossovers in RGC-cKO retinas that were similar in expressivity to *Sema6a^LacZ/LacZ^* retinas (Sun et al., 2013), but loss of *Sema6a* in all ACs was not sufficient to disrupt the SAC scaffolds. This suggests that normally RGC- but not AC-derived Sema6A regulates SAC scaffold segregation; otherwise, crossovers would have been evident in AC-cKO retinas. Similarly, we identified a specific role for RGC- but not for AC-derived Sema6A in final RGC positioning. AC-cKO, however, severely disrupted ON-SAC plexus organization, suggesting functional distinctions between AC- and RGC-derived Sema6A in inner retinal patterning. Consistent with specific roles for RGC-derived Sema6A in SAC segregation, Sema6A is expressed by more neonatal RGCs than ACs, and Sema6A is enriched in ON (S4) versus OFF (S2) DSGC dendrites. The sharp S4 band of Sema6A is lost in RGC-cKO retinas, indicating that RGCs establish asymmetric Sema6A expression potentially capable of segregating the SAC scaffolds. This asymmetric expression is developmentally maintained and exemplified in AC-cKO retinas (Fig. 8). Furthermore, we observed further compensatory upregulation of Sema6A in presumptive AC neurites adjacent to the GCL after RGC-cKO, but we did not observe this phenomenon after AC-cKO, supporting the idea that AC-derived Sema6A is normally dispensable for SAC segregation. For these reasons, we favor the parsimonious interpretation that RGC-derived Sema6A normally influences SAC scaffold segregation, mirroring the transient role for RGCs in organization of zebrafish AC neurites (Kay et al., 2004).

The compensatory upregulation of Sema6A suggests that ACs sense Sema6A to ensure sufficient levels for assembly of distinct OFF- and ON-SAC IPL circuits, which enables the downstream matching of pre- and postsynaptic retinal neurites (Duan et al., 2018). Arc-GFP-expressing ACs, the only neuron type that robustly expresses Sema6A in an adult mouse retina transcriptomic profiling database (Siegert et al., 2012), stratify neurites adjacent to the GCL (Siegert et al., 2009), suggesting they represent the failsafe Sema6A-sensing cell type that ensures ON versus OFF retinal development.

The greater abundance of Sema6A in ON (S4) versus OFF (S2) DSGC dendrites may result from the predominance of oDSGC dendritic arbors in S4 versus S2 (Dhande et al., 2013). DSGC dendrites are not fixed in discrete IPL laminae by P1 (Kim et al., 2010) when SAC lamination is complete (Ray et al., 2018), but instead occupy the IPL in a graded fashion, with increased density adjacent to the GCL (Peng et al., 2017), positioning them to establish an asymmetric Sema6A boundary to segregate ON- and OFF-SAC scaffolds. We propose that DSGC dendrites during early retinal development first repel all SAC dendrites. Subsequently, a push/pull mechanism may sort SAC dendrites. Sema6A on RGCs could push developing SAC arbors away from the GCL, while a select pull mechanism between ON-SAC and oDSGC dendrites driven by an unknown factor, e.g. CNTN5-mediated attraction (Peng et al., 2017) or FLRT-LPHN adhesion (Prigge et al., 2023), counters repulsion of the ON-SAC arbor to position it closer to the GCL. Although *Cntn5* loss of function does not impair SAC dendrite segregation, ooDSGCs and ON-SACs express additional CNTNs, contactin-related protein (Caspr) and other adhesion molecules (Rheaume et al., 2018; Peng et al., 2020), supporting the hypothesis that adhesive mechanisms promote basal-positioning of ON-SAC dendrites and SAC scaffold segregation.

Our analysis of Sema6A signaling during retinal development showcases a general requirement for SAC scaffolds in elaborating the IPL, a crucial early step for ON versus OFF circuit elaboration. SAC scaffold development is in part regulated by Sema6A–PlexA2 signaling in both ON- and OFF-SACs. However, the early segregation of SAC dendrites, which is required for overall IPL patterning, is unexpectedly regulated by Sema6A in RGCs, the earliest born neurons in the retina.

## MATERIALS AND METHODS

### Animals

All animal experiments and procedures were approved by the Johns Hopkins Animal Care and Use Committee of Johns Hopkins University. Mice (*Mus musculus*) were maintained in pathogen-free facilities at Johns Hopkins University under standard housing conditions with continuous access to food and water. Histological studies per performed using embryonic day 14.5 (E14.5) to adult mice, as indicated. Mice of both sexes were used, and no sexual dimorphisms were observed in any results reported here. None of the mice had noticeable health or immune status abnormalities, and were not subject to prior procedures. The genotype of mice is described where appropriate. For all genetic experiments, an *n* value of at least three retinas from at least two animals was examined, unless otherwise noted. For several of the complicated genetic crosses presented, generating additional animals was not feasible due to limitations in obtaining viable breeders (e.g. only *Megf10C^re/Cre^*; *VGlut2^Cre/+^*; *Sema6a^+/−^* but not *Megf10C^re/+^*; *VGlut2^Cre/+^*; *Sema6a^+/−^* females yielded pups that were *Megf10C^re/+^; VGlut2^Cre/+^; Sema6a^HA-F/−^*). *Megf10* and *Sema6a* are genetically linked, and we did not think it was ethical to continue breeding to increase our *n* values. Littermates were used as control animals, unless otherwise noted.

Several mouse lines were used. In *Sema6a^HA-F^*, a hemagglutinin (HA) tag was knocked in downstream of the signal peptide in exon 2 at the N-terminus of the extracellular domain of Sema6A. *LoxP* sites were inserted in introns flanking exon 3. The insertion was verified by sequencing. Recombination between the *loxP* sites introduces a frameshift and multiple premature stop codons, preventing expression of Sema6A. *Sema6a^HA-F^* was generated by the Gene Targeting & Transgenics Facility at Janelia Research Campus.

To generate the *Sema6a* null allele, a *Sema6a^HA-F^* male was bred with a *Sox2^Cre^* (see below) female, in which Cre recombinase is expressed in the germline. Sema6a^HA-F^* ^/+^; Sox2^Cre/+^ (* indicates recombined cKO allele) offspring were then backcrossed to C57BL/6 wild-type mice for five generations to (1) segregate *Sox2^Cre^* away from the Sema6a null allele and (2) create an isogenic stock.

In *Sox2^Cre^* (Jackson Labs 008454, Hayashi et al., 2002), Cre recombinase is expressed under control of the mouse SRY-box containing gene 2 promoter. In *Sox2^Cre^* animals, Cre recombinase activity is detected in the epiblast cells as early as embryonic day 6.5; Cre expression is present in the female germline.

In *Six3^Cre^* (Jackson Labs, 019755, Furuta et al., 2000), Cre recombinase is expressed under control of the sine oculis-related homeobox 3 (Six3) promoter. Six3 is a transcription factor that is required for the development of the eye. *Six3^Cr^*^e^ is expressed ubiquitously in the central retina, but is largely absent in the peripheral retina (Ray et al., 2018).

In *Chat^Cre^* (Jackson Labs 006410, Rossi et al., 2011), Cre recombinase activity is weakly evident in neonatal SACs and increases over early postnatal development (Ray et al., 2018).

In *ROSA-LSL^tdTomato^*, also known as Ai14 (Jackson Labs, 007914, Madisen et al., 2010), the red fluorescent variant tdTomato is expressed in a tissue-specific manner, depending on the cell type-specific expression of Cre recombinase.

In *Chat^CreER^* (Jackson Labs, 008364, Rotolo et al., 2008), tamoxifen-inducible Cre recombinase is expressed in neonatal SACs in the retina and across development upon administration of tamoxifen.

In *Sema6a^LacZ^*, a *Sema6a* gene trap mouse line (Leighton et al., 2001) was determined here to be a hypomorphic allele.

In *Megf10C^re^*, Cre was inserted in the *Megf10* locus so that endogenous MEGF10 expression was preserved, immediately downstream of the MEGF10 coding sequence, with the Megf10 and Cre reading frames separated by a T2A self-cleaving peptide sequence (Peng et al., 2020).

In *ROSA^LSL-Synaptophysin-tdTomato^* mice [B6;129S-*Gt(ROSA)26Sor^tm34.1(CAG-Syp/tdTomato)Hze^*/J, Jackson Labs, 012570], a *loxP*-STOP-*loxP* cassette prevents expression of a synaptophysin::tdTomato fusion protein in the absence of Cre recombinase.

In *Sema6a^CreER^*, a targeting construct was designed to insert the coding sequence for tamoxifen-inducible Cre recombinase fused with the mouse G525R mutant estrogen receptor 1 (ER; Schwenk et al., 1998) downstream of the stop sequence (which was removed) in exon 19 of the *Sema6a* locus. A furin cleavage RAKR sequence was placed directly downstream of the Sema6- coding sequence to separate Sema6A and the CreER fusion protein. A 2xV5 linker was added immediately downstream of the furin cleavage sequence to label the N-terminal fragment of the subsequent P2A self-cleaving peptide separating the Sema6A- and CreER-coding sequences. The *Sema6a^CreER^* allele was generated by the Gene Targeting & Transgenics Facility at Janelia Research Campus.

In *Plxna2^R1746A^* (Zhao et al., 2018), here referred to as *Plxna2^ΔRasGAP^*, CRISPR/Cas9 gene editing was used to generate a point mutation that replaces a catalytically essential arginine reside with alanine, rendering *Plxna2^ΔRasGAP^* deficient in GTPase activating protein (GAP).

In *Plxna2* nulls, insertion of the ITak-Neo cassette in the *Plxna2* locus generates only the truncated PlexA2 signal peptide (Suto et al., 2007).

In *Plxna4* nulls, insertion of the PGK-neo cassette replaces exons 18 and 19, which contain the transmembrane domain, of *Plxna4*, generating a null allele (Yaron et al., 2005).

In *VGlut2^Cre^* [B6J.129S6(FVB)-*Slc17a6tm2(cre)Lowl*/MwarJ, Jackson Labs, 028863], Cre recombinase is expressed in glutamatergic neurons under the control of the vesicular glutamate transporter 2 promoter (Vong et al., 2011).

In *Sema6a^ΔCyt^*, exon 19 of the *Sema6a* locus, which includes the intracellular domain (ICD) coding sequence of Sema6A, was flanked by loxP sites. Cre-dependent recombination of this allele generates Sema6A lacking its ICD that can act only as a ligand. A complete description of this line is presented elsewhere (Verhagen et al., 2023 preprint).

In *Ptf1a^Cre^*, the Cre-coding sequence replaces the coding sequence for Ptf1a at the translation initiation codon (Kawaguchi et al., 2002). The basic helix-loop-helix transcription factor *Ptf1a* is expressed in precursors of amacrine and horizontal cells from E12.5 to P3 in the murine retina (Nakhai et al., 2007).

### SAC culture

P0-P2 *Chat^Cre/+^; ROSA-LSL^tdTomato/+^* (Fig. S1E) or *Sema6a^HA-F/HA-F^* (Fig. S4) retinas were dissociated using Worthington Biochem Papain supplemented with DNase I and L-Cysteine, and were cultured according to Lefebvre et al. (2012). Briefly, SACs were cultured on poly-D-lysine hydrobromide-coated glass coverslips at a density of 15,000-20,000 cells per well for 8-10 days, with media refreshment every 2-3 days: one-third of the media was replaced with fresh media. Importantly, 15% of mouse cortical astrocyte-conditioned media was added to the RGC culture medium. Astrocyte-conditioned media was prepared from astrocyte cultures prepared as described by Albuquerque et al. (2009). SACs in Fig. S1E were identified by virtue of tdTomato expression. SACs in Fig. S4 were identified by their unique radial morphology and lack of an axon. Density was optimized to yield cultures with isolated SACs, since SAC morphology *in vitro* is compromised by contact other cells. Details of all reagents are provided in Table S1.

### Immunohistochemistry

Mice (*Mus musculus*) were euthanized by continuous exposure to CO_2_ followed by cervical dislocation. Eyes were harvested and fixed with 4% PFA for 1-2 h at room temperature and rinsed with PBS. For retinal cross sections, the lens was removed, and eyes were immersed in 30% sucrose until sinking before embedding in Neg-50 frozen section medium (Fisher). Retinas were cryosectioned at 20 µm onto Superfrost Plus slides (Fisher). Sections were washed briefly in PBS and permeabilized in 0.02% PBST+10% host antibody compatible serum. Antibodies diluted in antibody diluent (0.02% PBST plus 10% host antibody compatible serum) were then incubated with sections overnight at 4°C in a humidified chamber. Sections were washed with PBS before incubating with secondary antibodies (1:500) and DAPI in antibody diluent for 1-2 h at room temperature in a humidified chamber. Sections were further washed with PBS before mounting in Fluoro-Gel with DABCO (EMS). For whole-mount retina staining, retinas were dissected and permeabilized in PBS/BSA/Triton-X permeabilization solution overnight with turning. Permeabilized retinas were incubated with primary antibodies in an antibody diluent consisting of 0.02% PBST plus 10% host antibody compatible serum with 20% DMSO for 5-7 days at room temperature with turning. Retinas were washed five or six times with PBS for 1 h at room temperature, with turning, before incubating in secondary antibodies (1:500) plus DAPI in antibody diluent for 1-2 days at room temperature with turning. Retinas were washed five or six times with PBS for 1 h at room temperature before mounting onto Superfrost Plus slides (Fisher) in Fluoro-Gel with DABCO (EMS). Broken coverslip pieces were placed at the four corners beneath the coverslip to prevent contact between the retinas and the coverslip. Slides were meticulously sealed with nail polish to prevent evaporation of the Fluoro-Gel. Details of all reagents and antibodies are provided in Table S1.

### Image acquisition and analysis

Images were acquired on Zeiss LSM 710 confocal microscopes with 405, 488-515, 568 and 647 lasers, processed using Zeiss ZEN software, and analyzed using ImageJ (NIH). Section images were acquired with a 20× air or a 40× oil lens at the resolution of 1024×1024 pixels and a step size of 0.5-1.5 µm. ImageJ (NIH) software was used to generate maximum intensity projections. Adobe Photoshop CS6 was used for adjustments to brightness and contrast.

### Surgical procedures

Intravitreal injections of AAVs (*AAV2-FLEX-GFP*, UNC Vector Core) or cholera toxin B (CTB, Life Technologies) were performed as previously described (Sun et al., 2015), with animals anesthetized using isofluorane (3% in O_2_). A 30-gauge needle was used to make an incision at the limbus. A 33-gauge Hamilton syringe was used to slowly introduce 1 µl of AAV or CTB; the needle was removed 30 s after the injection, to allow for dispersion of the solution into the intravitreal space. For AAV experiments, tissue was collected 2 weeks after injection, to allow for sufficient expression. The AAV reporter did not penetrate deeply enough to sparsely label OFF-SACs, precluding OFF-SAC arbor characterization in these studies. For CTB injections before eye opening, the fused eyelid was opened using Vannas spring scissors (Fine Science Tools, 15003-08). Animals were allowed to recover on a heated pad.

For enucleation, animals were deeply anesthetized using isofluorane (3% in O_2_), and enucleation was performed as described by Lim et al. (2016). Briefly, tissue surrounding the eye was depressed slightly with blunt forceps, curved surgical scissors (ROBOZ, RS-5675) were used to slightly elevate the eye from the orbit, and the optic nerve was swiftly cut and the eye removed. Afterwards, a silver nitrate applicator was gently and swiftly (∼1 s) turned in the orbit to cauterize the tissue. Animals were recovered on a heated pad. CTB was intravitreally injected in the remaining eye at P14 before tissue collection at P15.

For tamoxifen-inducible expression of *ROSA-LSL^tdTomato^* and *ROSA-LSL-Synaptophysin^tdTomato^* using *Sema6a^CreER^*, tamoxifen was injected into the milk sac of P1-P3 pups; to examine expression at P6-P14, tamoxifen was injected subcutaneously (*n*=3 per timepoint). For neonatal recombination of *Sema6a^HA-F^* in Fig. 2, tamoxifen was injected into the milk sac of P0.5 pups, after milk was evident.

### Smart-Seq2 scRNA-seq

Single cell mRNA sequencing was performed using a modified Smart-seq2 protocol that has been described in detail elsewhere (Chevée et al., 2018). Briefly, 4-6 retinas from 3-6 *Chat^Cre/+^; ROSA-LSL^tdTomato/+^* mice were isolated at P1, P4, P8, P11 and P16 in ice-cold HBSS, and the retinal pigmented epithelium and inner limiting membranes were removed. Retinas were digested with papain, triturated slowly 15-20 times with a P1000 pipet tip, strained into a 50 ml conical tube, and pelleted for 3 min at 1300 rpm (340 ***g***). The pellet was gently resuspended and cells were washed three times in ice-cold Hibernate A media, with pelleting between the washes. Care was taken to tap the tube to dislodge the pellet gently before adding media and resuspending the pellet, in order to prevent cell lysis and subsequent accumulation of DNA and cellular debris, which forms as a sticky gel-like substance in the supernatant. Washed and resuspended cells in ice-cold Hibernate A were subjected to FACS to isolate single cells in cell lysis buffer before proceeding with Smart-seq2 mRNA sequencing as described by Chevée et al. (2018). Libraries were sequenced with the Illumina NextSeq platform at the Johns Hopkins Genetics Research Core Facility to generate 75 bp paired-end reads at mid output (150M reads). Details of all reagents are provided in Table S1.

### Primer sequences

Primer sequences were as follows: oligo-dT30VN, /5Biosg/AAGCAGTGGTATCAACGCAGAGTACTTTTTTTTTTTTTTTTTTTTTTTTTTTTTTVN; ISPCR, /5Biosg/AAGCAGTGGTATCAACGCAGAGT; TSO, /5Biosg/AAGCAGTGGTATCAACGCAGAGTACATrNrG+G.

scRNA-seq FASTQ files were aligned to the mouse reference genome GRCm38.p6 (mm10) using HISAT2 v2.1.0 (Kim et al., 2015). SAM files were converted to BAM files using SAMtools v1.3.1. Transcript abundance was quantified using Cufflinks v2.2.1 (Trapnell et al., 2012). FPKM values from Cufflinks were used as input for downstream analyses in the Monocle2 framework (Trapnell et al., 2014) in RStudio v1.4.1103 or Seurat (Satija et al., 2015) in R v3.6.2, to generate violin plots. Normalized FPKM values were converted to estimated copies per cell using the Census algorithm (Qiu et al., 2017). Cells with expression of >2000 and <50,000 mRNAs were selected for further analysis. Analyzed cells had >425,000 reads/cell and >3900 genes/cell. OFF- and ON-SAC clusters were defined by virtue of *Rnd3* and *Fezf1* expression (Peng et al., 2020). The total number of ON- and OFF-SACs sequenced and analyzed at each timepoint are provided in Table 1.

**Table 1.**
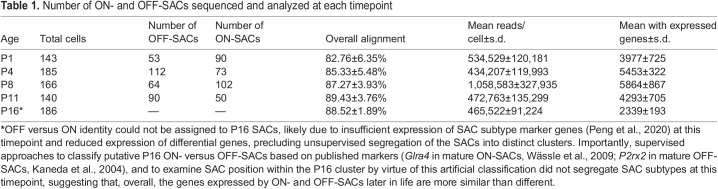
Number of ON- and OFF-SACs sequenced and analyzed at each timepoint

### Quantification and statistical analysis

Whenever possible, an *n*≥3 animals was quantified for each phenotype assessed, except in extreme instances of breeding limitations, as described above. Sample size was projected based on the effect size observed for comparable analyses by Sun et al. (2013). Using these historical sample sizes, we were sufficiently powered to detect robust significant differences, as described. In Fig. 1, the germline and pan-retinal *Sema6a* cKO SAC lamination defects were too pronounced to score single crossovers between the OFF and ON strata. To overcome this, we drew and measured lines in ImageJ to define the occurrence of parallel and uninterrupted ChAT^+^ SAC immunolabeling between the OFF (S2) and ON (S4) SAC strata per 320 µm in 320 µm×320 µm images. An *n*≥3 animals per genotype was quantified, with three images per retina averaged, and the value of both retinas per animal averaged. For crossovers between S2 and S4 (Fig. 4B, Fig. 8B and Fig. S8C), ChAT^+^ signal spanning both strata that was ≥2 µm wide was considered a bona fide crossover, and independent crossovers were scored as such when separated by at least 20 µm. Given this stringent scoring method, which was chosen to exclude duplicate scoring of single arbors crossing over more than once and to capture crossovers of independent SACs/SAC arbors, it is possible that crossover events were undercounted. SAC dendritic field areas were quantified in ImageJ by joining a segmented line drawn around the perimeter of the dendritic arbors, and SAC symmetry indices were calculated as described by Sun et al. (2013) (*n*, number of SACs, is presented in each quantification).

To quantify retinal neurite phenotypes related to loss of *Sema6a* (Fig. 5), including PKCɑ^+^ and HCN4^+^ axon termination zones and VGlut3^+^ AC neurite lamination deficits, a max projection of five to eight optical sections from *z*-stack images was generated for three or four retinal sections per retina. A perimeter was drawn around the specific neurite signal and the area was measured in Image J. This neurite area was normalized to the total area of the retinal section, as outlined by DAPI. Retinal averages per genotype were presented in quantifications. When quantifying RBPMS^+^ RGC displacement in whole-mount retinas, a *z*-stack image was acquired in each quadrant of a whole-mount retina flattened by four releasing cuts placed ∼90° apart. The image was centered as much as possible along the central to peripheral retinal axis in each quadrant. Total RGCs in register with DAPI nuclear counterstain in each 320 µm×320 µm image were counted throughout the stack, corresponding to RGCs in the GCL, IPL and INL. Quadrant values were averaged to generate a value (% of displaced RGCs, or total RGCs) per retina. To quantify neonatal RGC displacement, max projections of four optical sections from *z*-stack images were generated for three or four retinal sections per retina. The RBPMS^+^ signal area apical to the magenta line defining the upper limit of the INBL (see Fig. 5N) was measured in retinal cross-sections, and this was normalized to the total area of the retinal section outlined by DAPI. The identities of displaced/lost RGCs remain to be determined. Melanopsin^+^ intrinsically photosensitive RGCs (ipRGCs) account for only ∼2.5% of total RGCs, with normally displaced ipRGCs representing ∼0.35% of the population (Valiente-Soriano et al., 2014). Thus, normally displaced ipRGCs do not significantly impact our quantifications of RGC displacement.

To quantify gaps in the ON-SAC plexus (related to Fig. 7), a line was drawn within each gap as its largest diameter, and only gaps larger than 20 µm were considered a bona fide gap, versus the normal holes within the honeycomb structure of SAC arbors. For quantification of HA-Sema6A protein in S5 of the IPL following *Sema6a* cKO in RGCs (Fig. 8C and Fig. S11D), the segmented line tool in ImageJ was used to draw a closed object around the ChAT^+^ ON-SAC band in S4. This object was then moved down into S5, adjacent to the DAPI^+^ nuclear GCL, and HA-Sema6A signal was measured. A segmented line encompassing the OPL was subsequently drawn and the HA-Sema6A signal was measured to act as an internal control for normalization of changes in HA-Sema6A in the IPL. The ratio of HA-Sema6A in S5/OPL for three images per retina for two mice per genotype was averaged per experiment (*n*=4 retinas from two mice).

All data are shown as mean±s.e.m. with *n* representing the sample number from at least four retinas from at least two mice, if not explicitly designated otherwise. For neuronal morphology quantifications, at least eight neurons were quantified. Power calculations were not performed to predetermine the sample size due to our limited ability to generate many mice with complicated genetic backgrounds. Data were not excluded from any analysis. Two-tailed Student's *t*-tests were used for comparisons between two groups, and one-way ANOVA with post-hoc Tukey's or Dunnett's multiple comparisons tests (MCTs) were used for multi-group comparisons, as indicated. Tukey's was used when comparing means of all groups in an experiment, and Dunnett's was used when comparing means of groups to a single control. *P<0.05; **P<0.01; ***P<0.001; ****P<0.0001.

## Supplementary Material



10.1242/develop.204293_sup1 Supplementary information
