# Supplementary material for: Retinal ganglion cell-derived semaphorin 6A segregates starburst amacrine cell dendritic scaffolds to organize the mouse inner retina
Source: Development. 2024 Nov 26;151(22):dev204293. doi: 10.1242/dev.204293 (PMC11634039; doi:10.1242/dev.204293)
Supplement: Supplementary information [file develop-151-204293-s1.pdf]

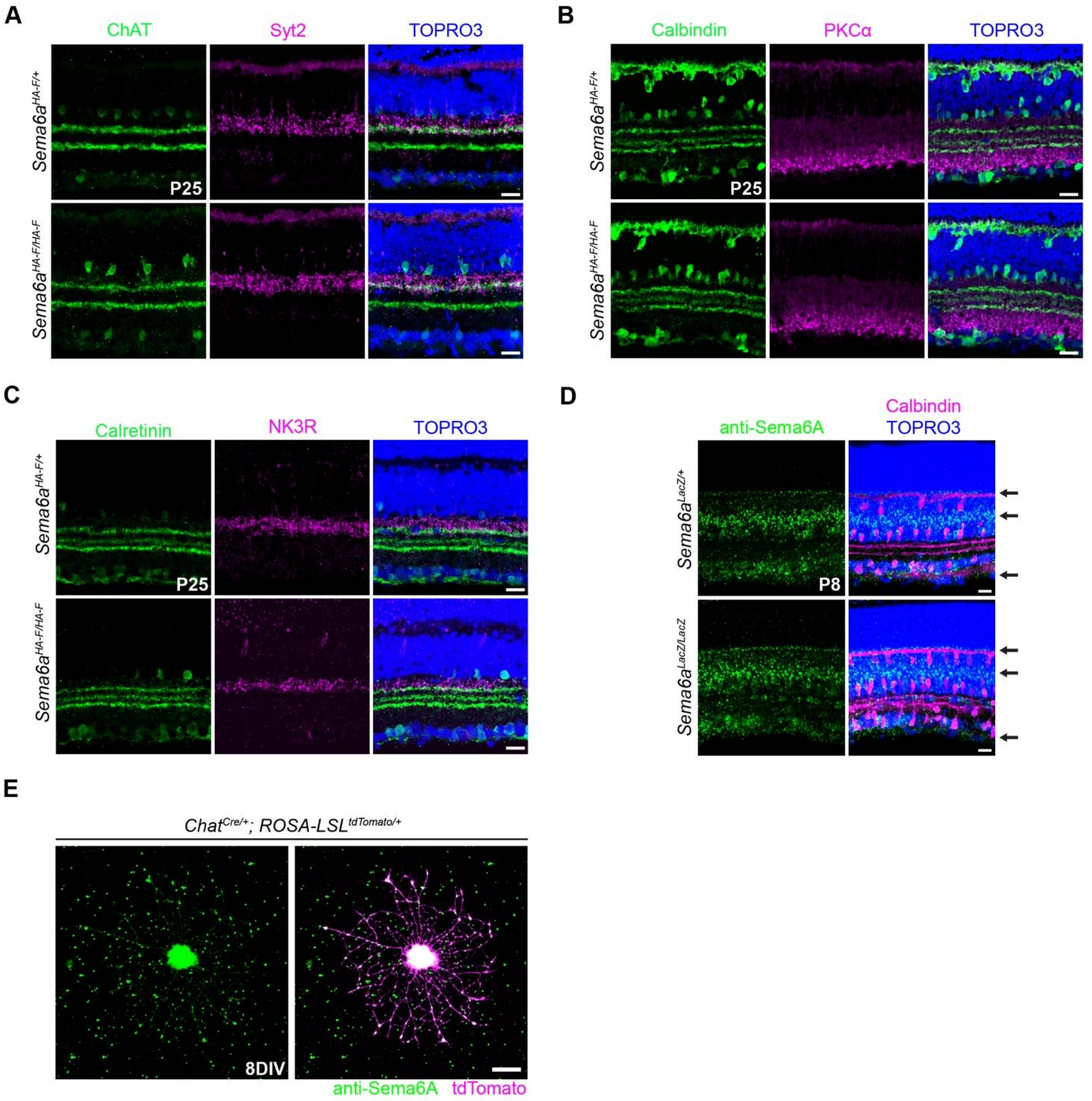

Fig. S1. The *Sema6a*$^{HA\text{-}F}$ allele is sufficient for retinal development, and characterization of anti-Sema6A immunolabeling non-specificity. P25 retinal cross sections demonstrating proper lamination of SACs (anti-ChAT, green) and type 2 OFF CBC axon terminals (anti-Syt2, magenta) **(A)**, calbindin^{+} ACs and RGCs (anti-calbindin) and rod BC axon terminals (anti-PKCα) **(B)**, and calretinin^{+} ACs and RGCs (anti-calretinin, green) and type 1 and 2 OFF CBC axon terminals (anti-NK3R, magenta) **(C)** in *Sema6a*$^{HA\text{-}F/HA\text{-}F}$ retinas. **(D)** P8 retinal cross sections demonstrating non-specific anti-Sema6A immunolabeling (black arrows). Despite the presence of Sema6A protein in *Sema6a*$^{LacZ/+}$ retinas (upper panels), the anti-Sema6A antibody does not reveal specific labeling

Development • Supplementary information

of Sema6A in the IPL (see Fig. 1B). **(E)** anti-Sema6A immunolabeling yields a highly speckled background signal in retinal cultures at 8 days *in vitro* (DIV). An abundance of signal is detected that does not coincide with genetically labeled *Chat*^{Cre}*; ROSA-LSL*$^{tdTomato/+}$ SACs or other cells. Scale bars, 20µm

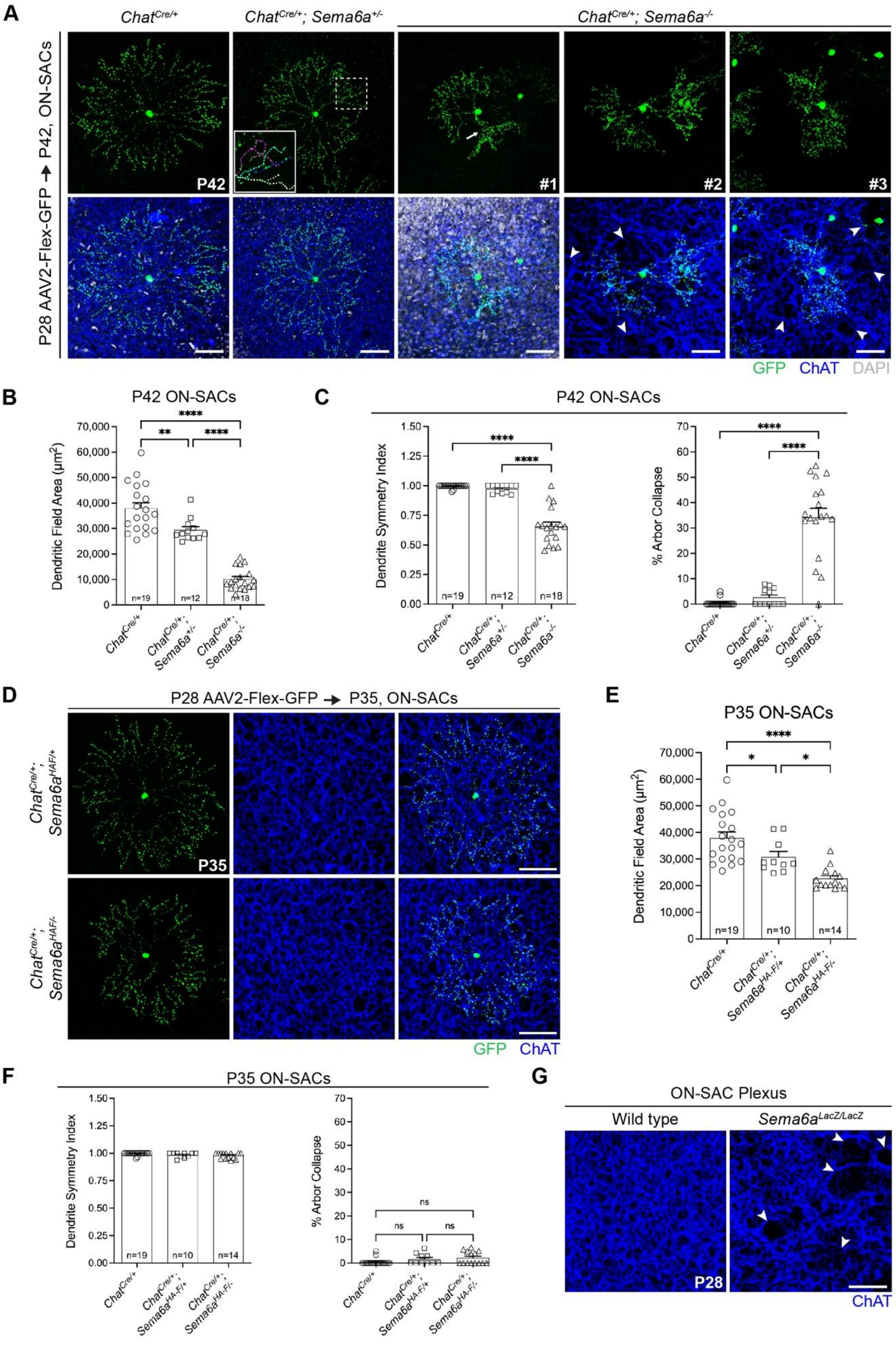

Development • Supplementary information

Fig. S2. ON-SAC phenotypes in various *Sema6a* mutant backgrounds. (A) Examples of ON-SACs in *Sema6a*$^{-/-}$ retinas labeled with AAV2-Flex-GFP. Some ON-SACs are missing portions of their dendritic arbor restricted to a single quadrant, while others are missing opposite quadrants (#2), or ~half of their overall dendritic arbors (#2, #3). Some *Sema6a*$^{-/-}$ ON-SACs were aberrantly bundled at the second branch point from the ON-SAC cell body (#1, arrow), while some failed to elaborate distinct primary dendrites (#3). #2 and #3 demonstrate that although the OFF- and ON-SAC layers are nearly fused in *Sema6a*$^{-/-}$ retinas (Fig. 5), large gaps are evident in the SAC plexus (white arrowheads). Quantification of ON-SAC dendritic arbor area **(B)**, symmetry **(C)**, and % arbor collapse **(D). (E)** ON-SACs of pan-SAC (*Chat*^{Cre}) *Sema6a* cKO retinas sparsely labeled by AAV2-Flex-GFP display impaired distal dendrite self-avoidance, but dendritic arbor radial symmetry is unaltered. Quantification of ON-SAC dendritic arbor area **(F)**, symmetry **(G)**, and % arbor collabpse **(H)** in *Chat*^{Cre} cKO retinas. **(I)** P28 *Sema6a*$^{LacZ/LacZ}$ mutant retinas have large holes and gaps in the ON SAC plexus (arrowheads). n, # of SACs. *, p<0.05; **, p<0.01; ****, p<0.0001; one-way ANOVA, Tukey's MCT. Scale bars, 50μm.

Development • Supplementary information

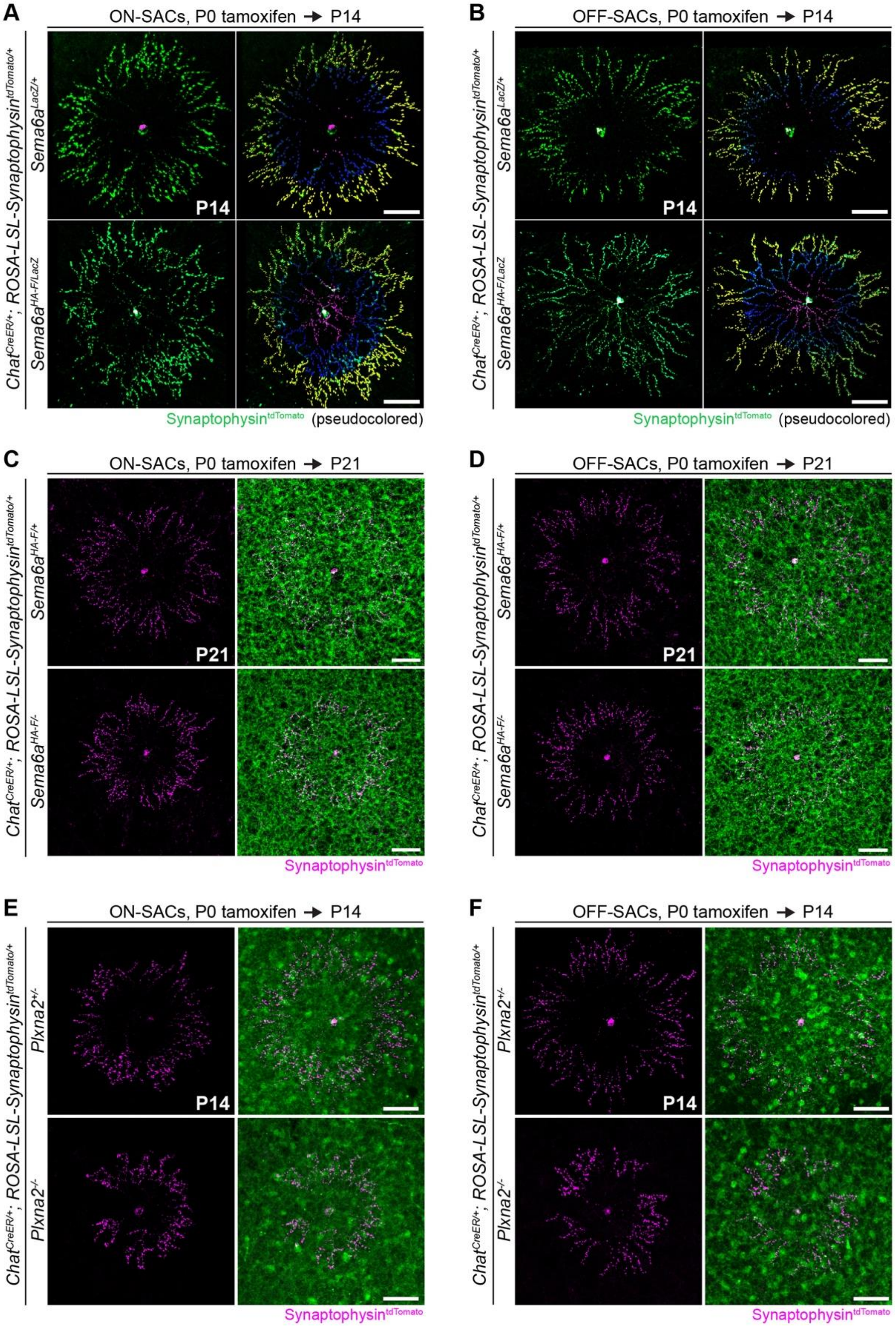

Development • Supplementary information

Fig. S3. Sema6A–PlexA2 signaling is not required for presynaptic specialization in SACs. Representative images of synapse distribution in P14 control and singly targeted *Chat*$^{CreER/+}$; *Sema6a*$^{HA\text{-}F/LacZ}$ ON- **(A)** and OFF-SACs **(B)** labeled by *ROSA-Synaptophysin-LSL*^{tdTomato}. The amount of tdTomato^{+} presynaptic puncta (pseudocolored green) localized to the inner (pseudocolored magenta) and middle (pseudocolored blue) thirds of the SAC dendritic arbor was increased in both ON- and OFF-SACs when *Sema6a* was conditionally removed from SACs in combination with the *Sema6a*^{LacZ} gene trap allele. Synaptic puncta (magenta) distribution at P21 was preserved when *Sema6a* was conditionally removed from isolated ON- **(C)** and OFF-SACs **(D)** in combination with the new *Sema6a* null allele. Synaptic puncta (magenta) distribution was similarly unaltered in *Plxna2*$^{-/-}$ ON- **(E)** and OFF-SACs **(F)**. *Sema6a* cKO and presynaptic labeling was induced by tamoxifen injection at P0 for all experiments. Scale bar, 50 µm.

Development • Supplementary information

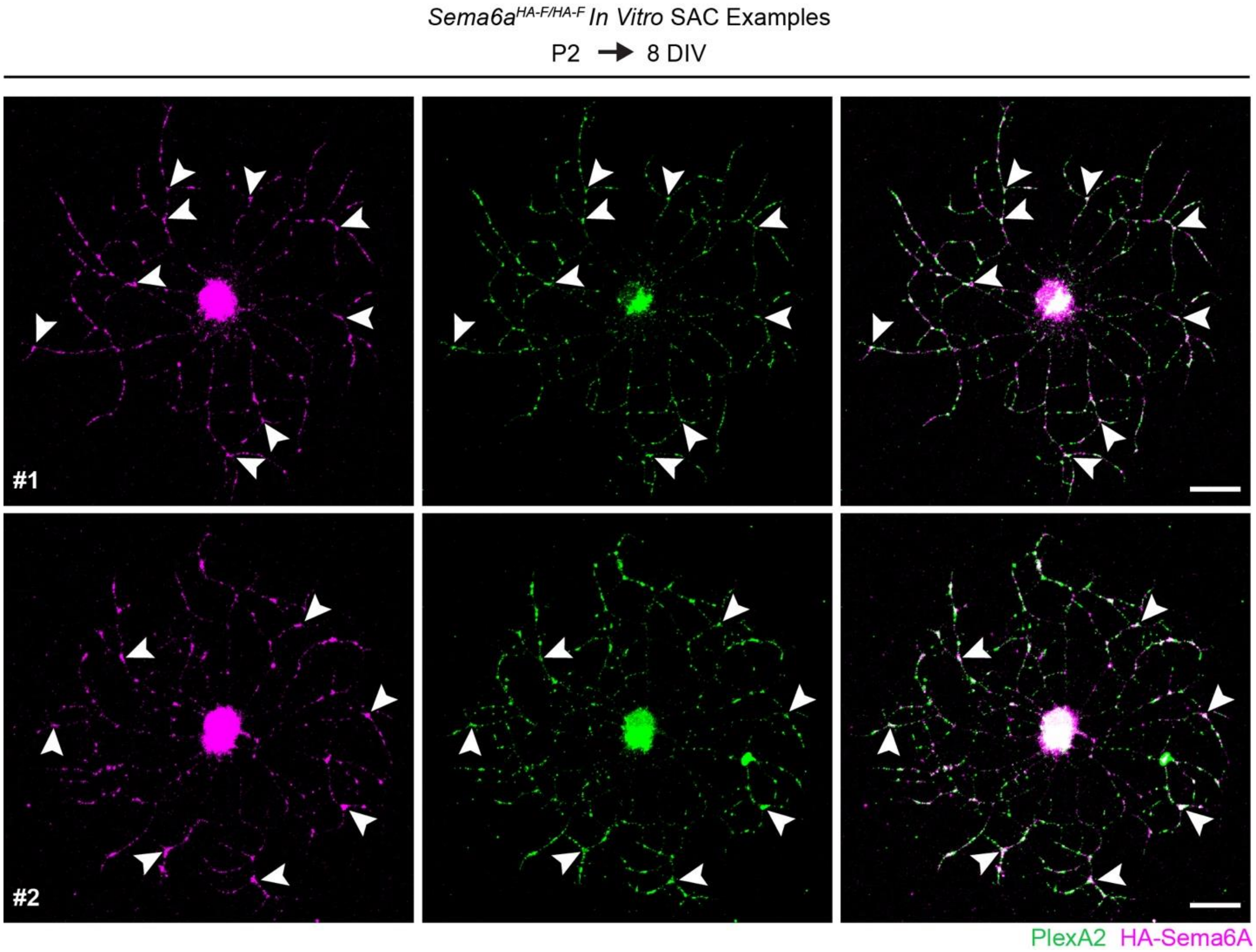

Fig. S4. Subcellular localization of Sema6A and PlexA2 in SACs *in vitro*. Representative SACs isolated from P2 *Sema6a*$^{HA-F/HA-F}$ retinas, cultured for 8 DIV. HA-Sema6A (magenta) and PlexA2 (green) are enriched in distal SAC dendrites and strongly colocalize at branch points (white arrowheads). Scale bars, 20µm.

Development • Supplementary information

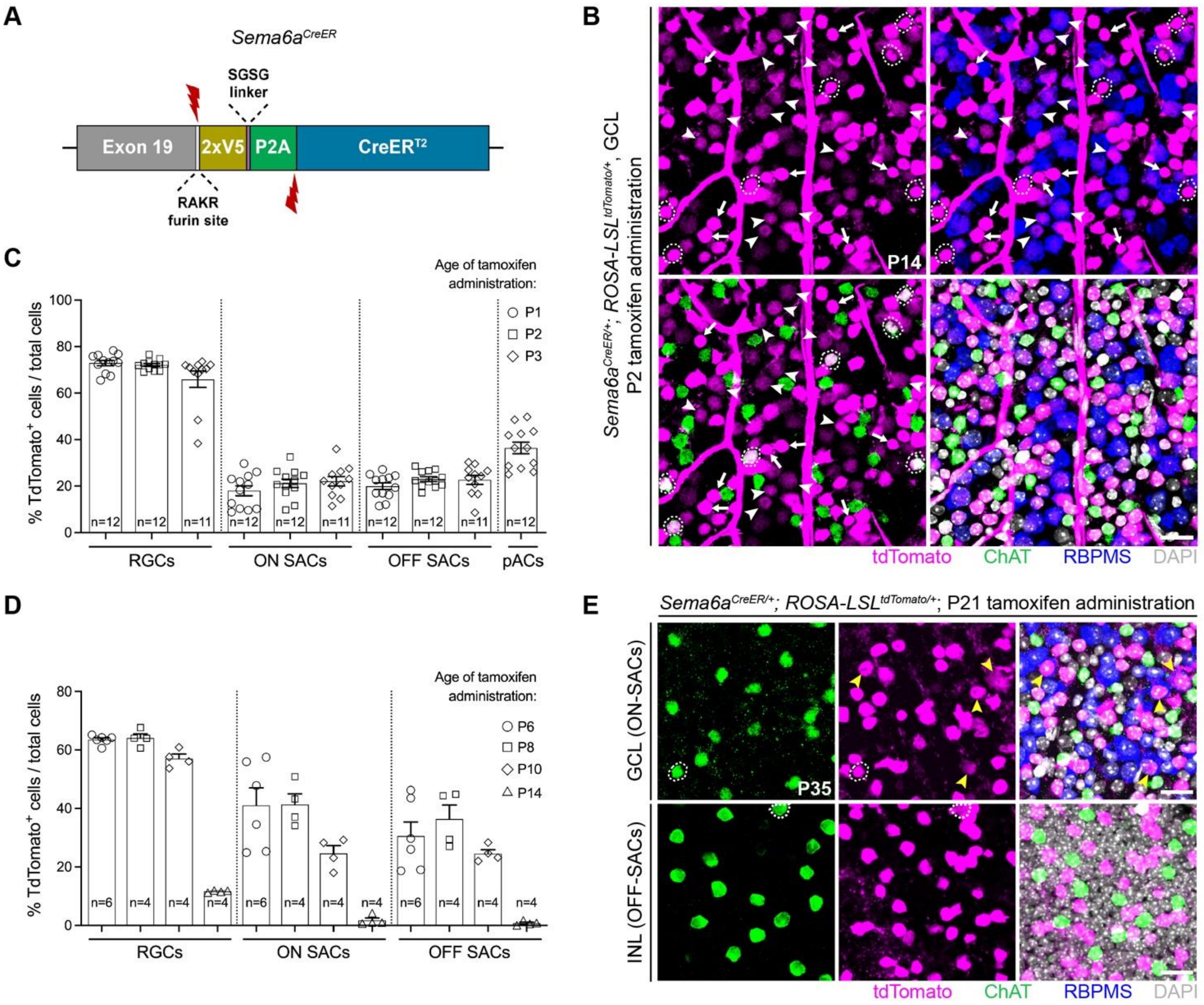

Fig. S5. *Sema6a* is robustly expressed by RGCs and displaced non-SAC ACs in the neonatal retina. (A) Model of the *Sema6a*^{CreER} knock-in allele. A Cre^{ER} cassette is knocked into exon 19 of the endogenous *Sema6a* locus. Red lightning bolts: engineered (furin RAKR) and P2A cleavage sites. **(B)** Representative image of tdTomato expression in a P14 *Sema6a*$^{CreER/+}$*; ROSA-LSL*$^{tdTomato/+}$ retina following P2 tamoxifen administration. tdTomato (magenta) is expressed by most RBPMS^{+} RGCs (blue; white arrowheads), some ON-SACs (green; outlined circles), presumptive displaced non-SAC ACs (white arrows), and endothelial cells. **(C)** Quantification of RGCs, ON-SACs, OFF-SACs, and presumptive non-SAC ACs (pACs) expressing *Sema6a* (tdTomato^{+}) at the indicated tamoxifen pulse timepoints, quantified at P14. **(D)** Quantification of RGCs, ON-SACs, and OFF-SACs expressing *Sema6a* (tdTomato^{+}) at the indicated tamoxifen pulse timepoints, quantified at P14 (for P6/P8 tamoxifen pulsed) or P21 (for P10/P14 tamoxifen pulsed). *Sema6a* transcriptional activity in SACs peaks at ~40% at P6 and P8, and substantially decreases by P14: only 1.75% of ON- and 0.77% of OFF-SACs exhibit *Sema6a* transcriptional

Development • Supplementary information

activity at P14. **(E)** Representative image of tdTomato expression in a P35 *Sema6a*$^{CreER/+}$; *ROSA-LSL*$^{tdTomato/+}$ retina following P21 tamoxifen administration. tdTomato (magenta) is expressed by most RBPMS^{+} RGCs (blue; white arrowheads), some ON-SACs (green; outlined circles), presumptive displaced non-SAC ACs (white arrows), and endothelial cells. Importantly, *Sema6a* is comparably expressed by ON- and OFF-SACs at all timepoints examined. Scale bar, 20µm.

Development • Supplementary information

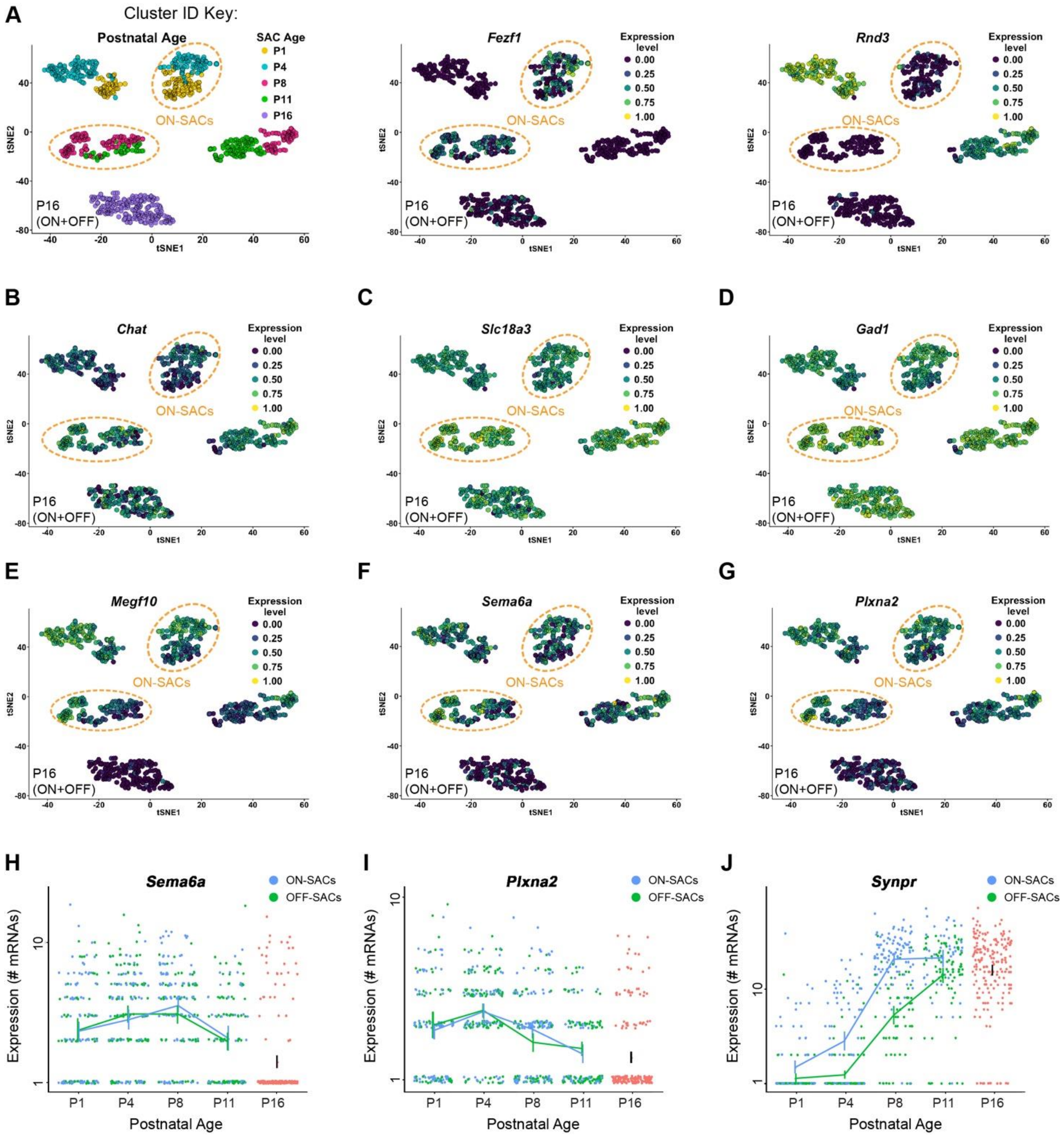

Fig. S6. SAC scRNA-seq across early postnatal development. (A) tSNE dimension reduction of gene expression profiles from *Chat*$^{Cre/+}$*; ROSA-LSL*$^{tdTomato/+}$ SACs at P1, P4, P8, P11, and P16 labeled by age (left), *Fezf1* expression (middle, ON-SAC marker), and *Rnd3* expression (right, OFF-SAC marker). Younger SACs (P1/P4) clustered together at the top of the plot, and subclusters correspond to ON- versus OFF-SAC identity. P8 and P11 SACs similarly clustered together in the middle of the tSNE plot and were segregated by SAC identity. At P16, ON- and OFF-SACs were not separable, likely owing to the downregulation of genes by P16 responsible

for conferring SAC identity and establishing ON vs. OFF circuits (see plots of *Fezf1, Rnd3, Megf10, Sema6a,* and *Plxna2*). Relative expression is depicted on a viridis scale: purple (no expression, "0.00") to yellow (high expression, "1.00") for tSNEs labeled with gene expression patterns. **(B-G) Normalized expression levels of the indicated transcripts. (B-C)** Known SAC markers *Slc18a3* (VAChT) and *Chat* are enriched in the dataset, as expected. *Slc18a3* and *Chat* are exclusively expressed by SACs in the retina (Brandon, 1987; Koulen, 1997). **(D**) Inhibitory interneuron marker *Gad1* (Kosaka et al., 1988) is enriched in the dataset, as expected. **(E)** SAC marker *Megf10* is strongly expressed by neonatal SACs and is downregulated over time, as expected (Kay et al., 2012). Accordingly, *Megf10* is not expressed in P16 SACs. **(F-G)** *Sema6a* and *Plxna2* have remarkably similar expression profiles. **(H-I)** Jitterplots of *Sema6a* and *Plxna2* expression across development. The average number of mRNA transcripts is comparable across all ages in both ON- (blue) and OFF-SACs (green). SAC identity is not separable at P16 (pink). **(J)** Levels of gene expression for the synaptic vesicle protein *Synpr* (synaptoporin) increase in all SACs as development proceeds, suggesting that the downregulation of other genes (eg. *Sema6a, Plxna2, Fezf1, Rnd3*) by P16 reflects a switch in transcriptional cascades away from circuit elaboration/development towards general circuit function (see also *Slc18a3*, *Chat*, and *Gad1*). This suggests that once specialized ON- and OFF-SAC circuits have formed, differentially expressed genes turn off and SAC subtypes become more similar to one another. Interestingly, *Synpr* expression lags in OFF-SACs and is significantly higher in ON-SACs at P4 and P8, suggesting that ON-SACs may mature earlier than OFF-SACs.

Development • Supplementary information

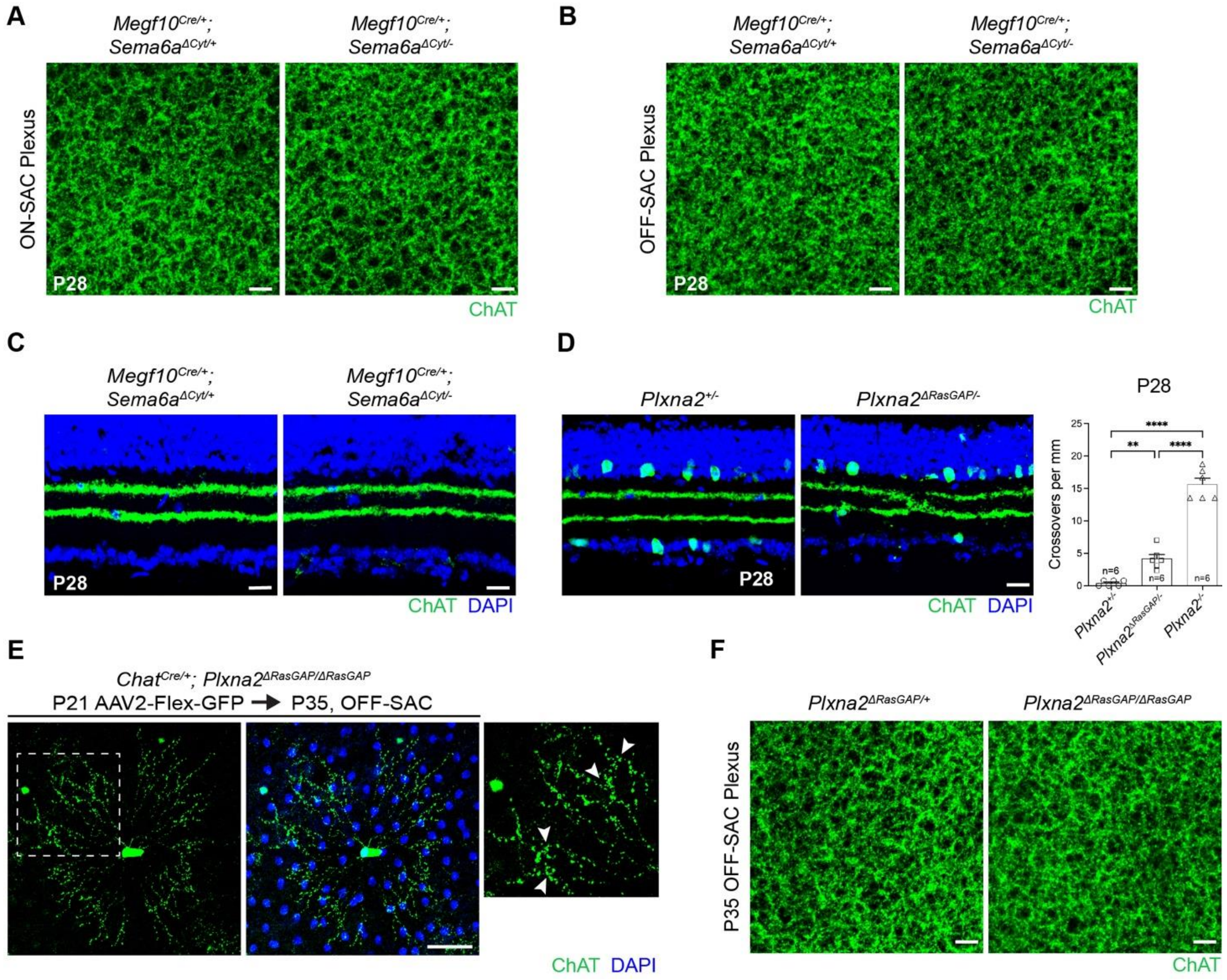

Fig. S7. Reverse Sema6A signaling in SACs is dispensable for SAC radial symmetry and dendritic lamination. P28 ON- **(A)** and OFF-SAC **(B)** plexus organization following conditional embryonic removal of the Sema6A cytoplasmic domain in all SACs using *Megf10*^{Cre}. The Sema6A cytoplasmic domain is not required in SACs for elaboration of their dendritic arbor plexuses. **(C)** P28 SAC lamination in Sema6A cytoplasmic domain deleted SACs. The cytoplasmic domain of Sema6A is not required for laminar segregation of ON- and OFF-SAC dendrites, as expected. **(D)** Representative image of SAC lamination in P28 *Plxna2*$^{1RasGAP/-}$ retinas (left). Quantification of SAC crossovers (right). **(E)** Representative image of a AAV2-Flex-GFP labeled OFF SAC from a *Chat*$^{Cre/+}$; *Plxna2*$^{1RasGAP/1RasGAP}$ retina. Examination of the few labeled OFF SACs obtained from these experiments revealed self-avoidance errors in distal dendrites (white arrowheads; dashed box corresponds to the high-resolution image of distal dendrites on the right). **(F)** P28 OFF SAC plexus organization in *Plxna2*$^{1RasGAP\ /1RasGAP}$

Development • Supplementary information

retinas compared to control. The rasGAP domain of PlexA2 is not required for OFF SAC plexus elaboration *en face*. n in **(D)**, # of retinas. **, $p<0.01$; ****, $p<0.0001$; one-way ANOVA, Tukey's MCT. Scale bars for **(A-B, E-F)**, 50µm. Scale bars for **(C-D)**, 20µm.

Development • Supplementary information

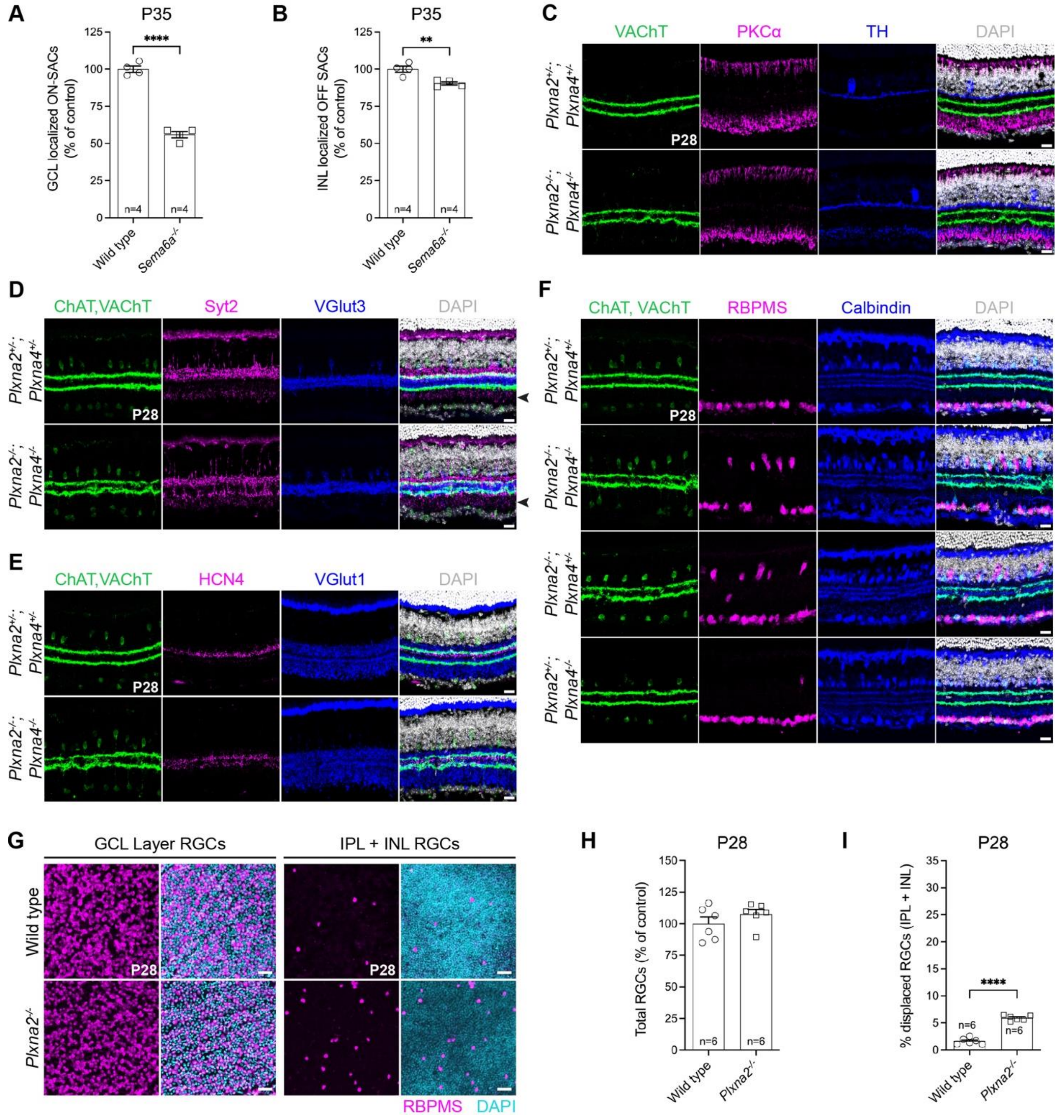

Fig. S8. ***Plxna2*** **and** ***Plxna4*** **only partially account for the range of phenotypes observed in** ***Sema6a*** **null retinas. (A)** Quantification of ON-SACs localized in the GCL. **(B)** Quantification of INL-localized OFF-SACs. **(C)** P28 retina cross sections labeled with anti-VAChT (SACs), anti-PKCα (rod BCs), and anti-tyrosine hydroxylase (TH^+ ACs). **(D)** P28 retina cross sections labeled with anti-ChAT and anti-VAChT (SACs), anti-Syt2 (type 2 OFF CBCs, intense labeling indicated by black arrow; type 6 ON CBCs, weak labeling indicated by black arrowhead), and anti-VGlut3 ($VGlut3^+$ ACs). **(E)** P28 retina cross sections labeled with anti-ChAT and anti-VAChT (SACs), anti-HCN4 (type 3a BCs), and anti-VGlut1 (BC axon terminals in the inner retina). **(F)** P28 retina cross sections labeled with anti-ChAT and anti-VAChT (SACs), anti-RBPMS (RGCs), and anti-calbindin (subtypes of RGCs and ACs, including SACs). **(G)** Whole mount retinas imaged through

the GCL (left) or the IPL + INL (right). Images represent Z-projections through the indicated regions and demonstrate mislocalization of $RBPMS^+$ (magenta) RGC cell bodies in the IPL + INL of *Plxna2*$^{-/-}$ mutant retinas. **(H)** Quantification of total RGCs for the indicated genotypes. **(I)** Quantification of displaced RGCs in the IPL + INL for the indicated genotypes. Wild type, C57BL/6. n, # of retinas. **, $p<0.01$; ****, $p<0.0001$; Student's t-test. Scale bars **(C-F)**, 20µm. Scale bar **(G)**, 40µm.

Development • Supplementary information

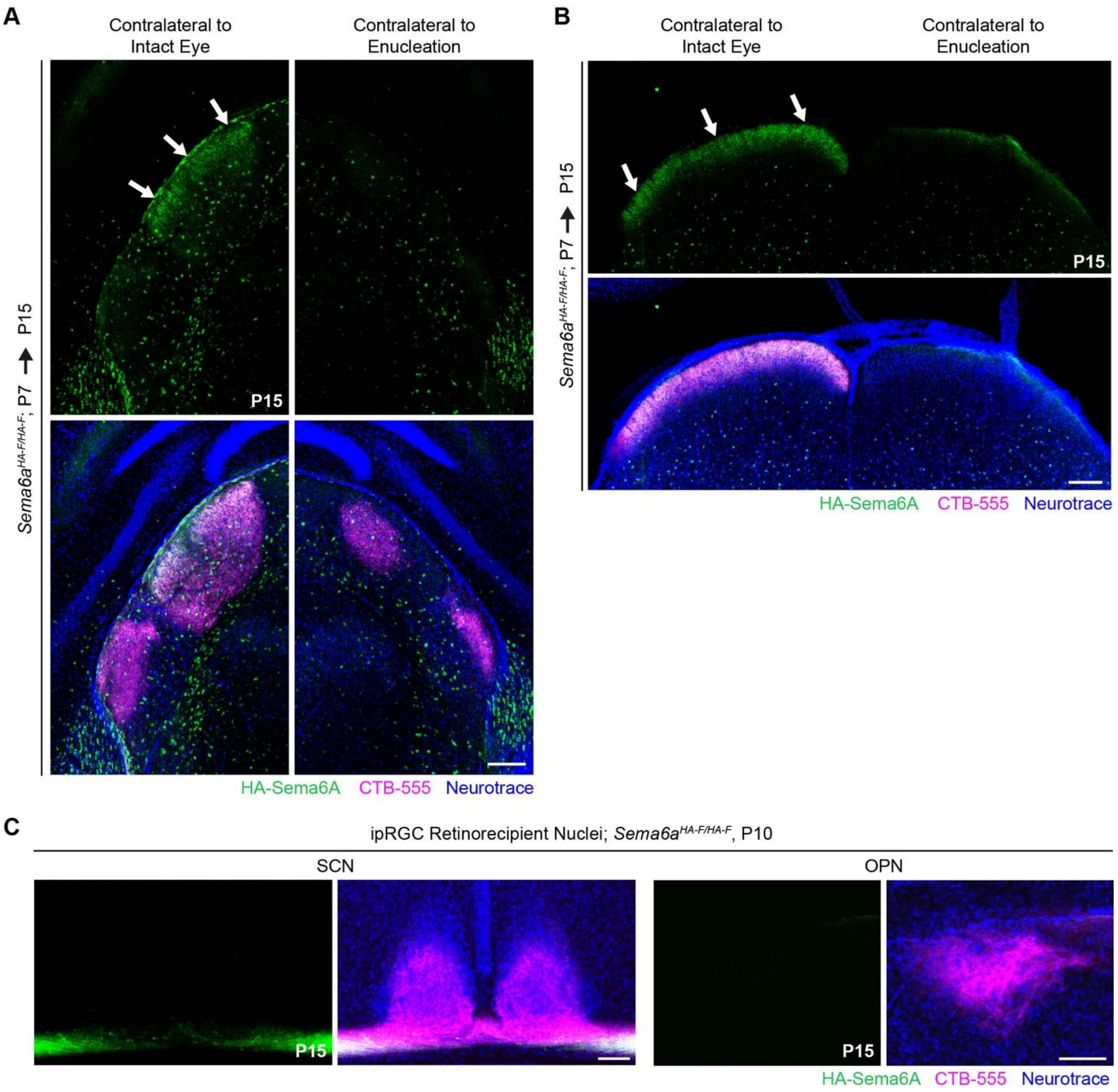

Fig. S9. Sema6A-expressing RGC axons project to image-forming visual targets. HA-Sema6A expression in *Sema6a*$^{HA\text{-}F/HA\text{-}F}$ mice was evaluated at P15 following P7 monocular enucleation; CTB-555 was injected at P14 to label retinorecipient midbrain nuclei. **(A-C)**. **(A)** HA-Sema6A is detected in the ooDSGC retinorecipient shell of the dLGN contralateral to the intact eye (white arrows). This signal is lost contralateral to the enucleated eye. Scale bar, 200µm. **(B)** HA-Sema6A is detected in the ooDSGC retinorecipient outer layer of the superior colliculus contralateral to the intact eye (white arrows) and is lost contralateral to the enucleated eye. Scale bar, 200µm. **(C)** HA-Sema6A is not detected in non-image forming ipRGC retinorecipient nuclei f P15 *Sema6a*$^{HA\text{-}F/HA\text{-}F}$ mice, including the suprachiasmatic nucleus (SCN) and the olivary pretectal nucleus (OPN). Scale bars, 100µm.

Development • Supplementary information

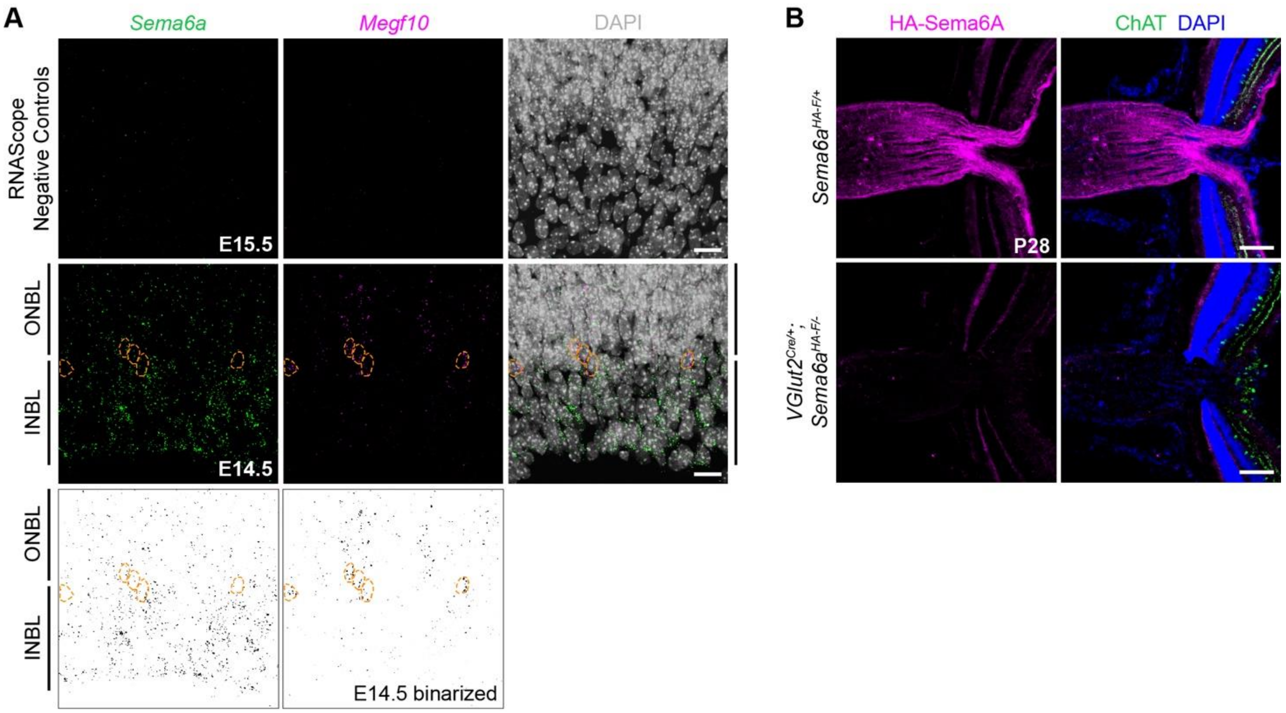

Fig. S10. Embryonic *Sema6a* expression and loss of Sema6A expression in RGC-specific cKO retinas. (A) RNAScope® *in situ* hybridization of *Sema6a* transcripts in wild-type embryonic tissue. *Sema6a* mRNA is not readily detectable in embryonic SACs identified by *Megf10* mRNA expression (orange dashed circles). *Sema6a* mRNA is enriched in the basal-most, RGC-rich inner neuroblastic layer (right bottom black bar). Scale bars **(A)**, 20µm. **(B)** Representative image of an RGC-specific $VGlut2^{Cre/+}$; $Sema6a^{HA\text{-}F/-}$ cKO retina cross section at P28 demonstrates a complete loss of Sema6A in RGC axons of the optic nerve compared to a $Sema6a^{HA\text{-}F/+}$ retina, validating RGC-specific *Sema6a* cKO. Scale bars **(B)**, 100µm.

Development • Supplementary information

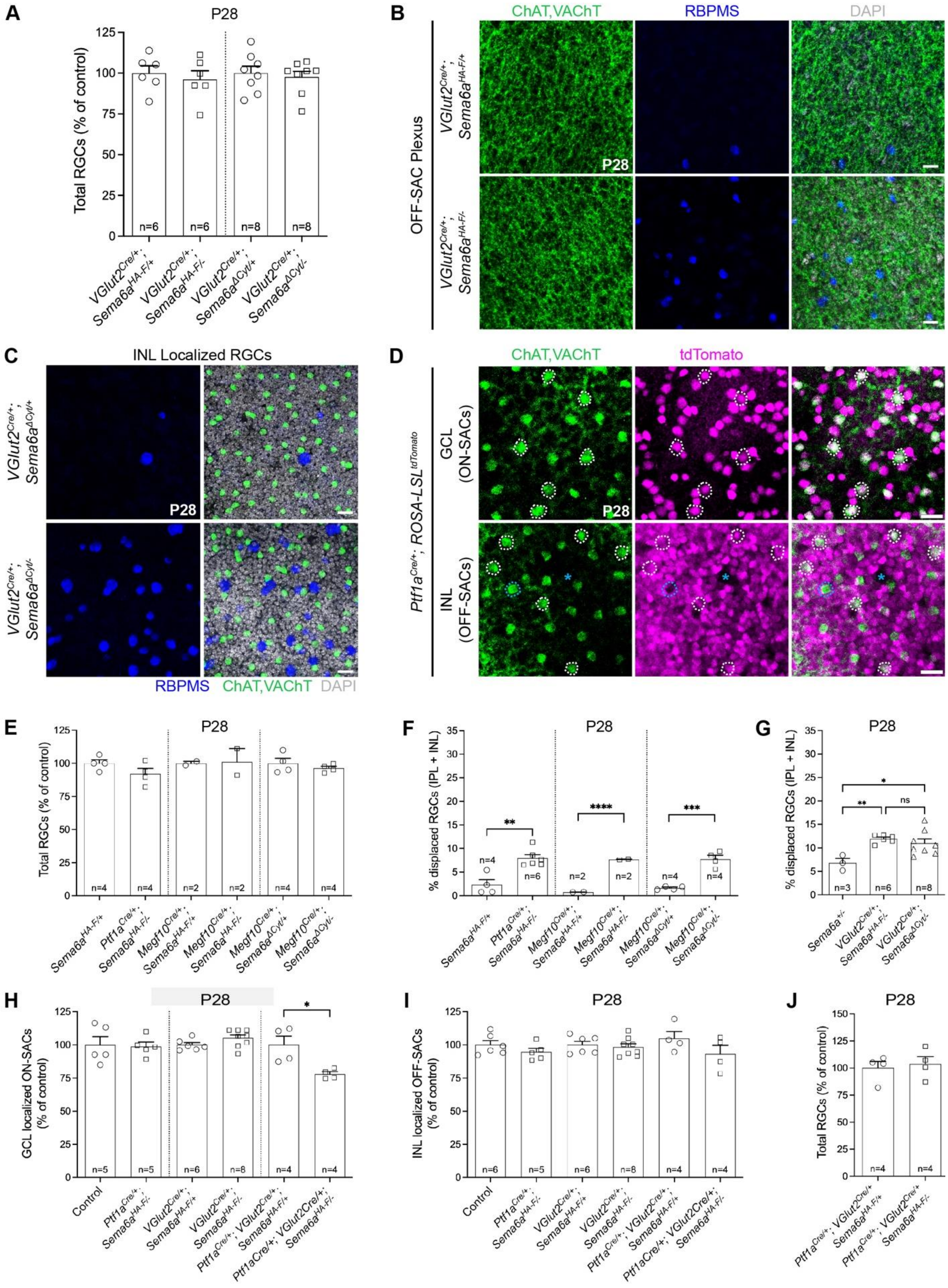

Development • Supplementary information

Fig. S11. Sema6A in RGCs prevents RGC mislocalization while Sema6A in ACs is normally dispensable for RGC localization. (A) Quantification of total RGCs for the indicated genotypes. **(B)** Representative images of the P28 OFF-SAC plexus (anti-ChAT) and RGC displacement (anti-RBPMS) in S2 of RGC-specific cKO retinas. **(C)** Representative images of RGC (anti-RBPMS) displacement into the INL (anti-ChAT) of RGC-specific Sema6A cytoplasmic domain cKO retinas. **(D)** *Ptf1a*^{Cre}-driven *ROSA-LSL*^{tdTomato} reporter expression in the GCL (top) and INL (bottom) at P28. ChAT^{+} SACs express tdTomato (white dashed circles). Dense tdTomato expression in the INL, consistent with Cre expression in all ACs, required image acquisition with minimal gain to avoid image oversaturation, limiting resolution of tdTomato in some cells (signal appears very low, but is present). Rare gaps lacking tdTomato expression were detected in the INL (cyan asterisk), however the majority of ACs express tdTomato. SACs lacking tdTomato expression were rarely observed (cyan dashed circle). **(E)** Quantification of total RGCs in various AC-specific *Sema6a* cKO backgrounds. **(F)** Quantification of displaced RGCs in various AC-specific *Sema6a* cKO backgrounds. **(G)** Quantification of ON-SACs localized in the GCL. **(H)** Quantification of INL- localized OFF-SACs. “Control” in AC cKO experiments in (G-H) correspond to pooled *Ptf1a*$^{Cre/+}$; *Sema6a*$^{HA\text{-}F/+}$ and *Sema6a*$^{HA\text{-}F/+}$ retinas. **(I)** Quantification of total RGCs in AC- and RGC-specific *Sema6a* cKO retinas. n, # of retinas. *, $p<0.05$; **, $p<0.01$; ***, $p<0.001$; ****, $p<0.0001$; Student’s t-test. Scale bars, 20μm.

Development • Supplementary information

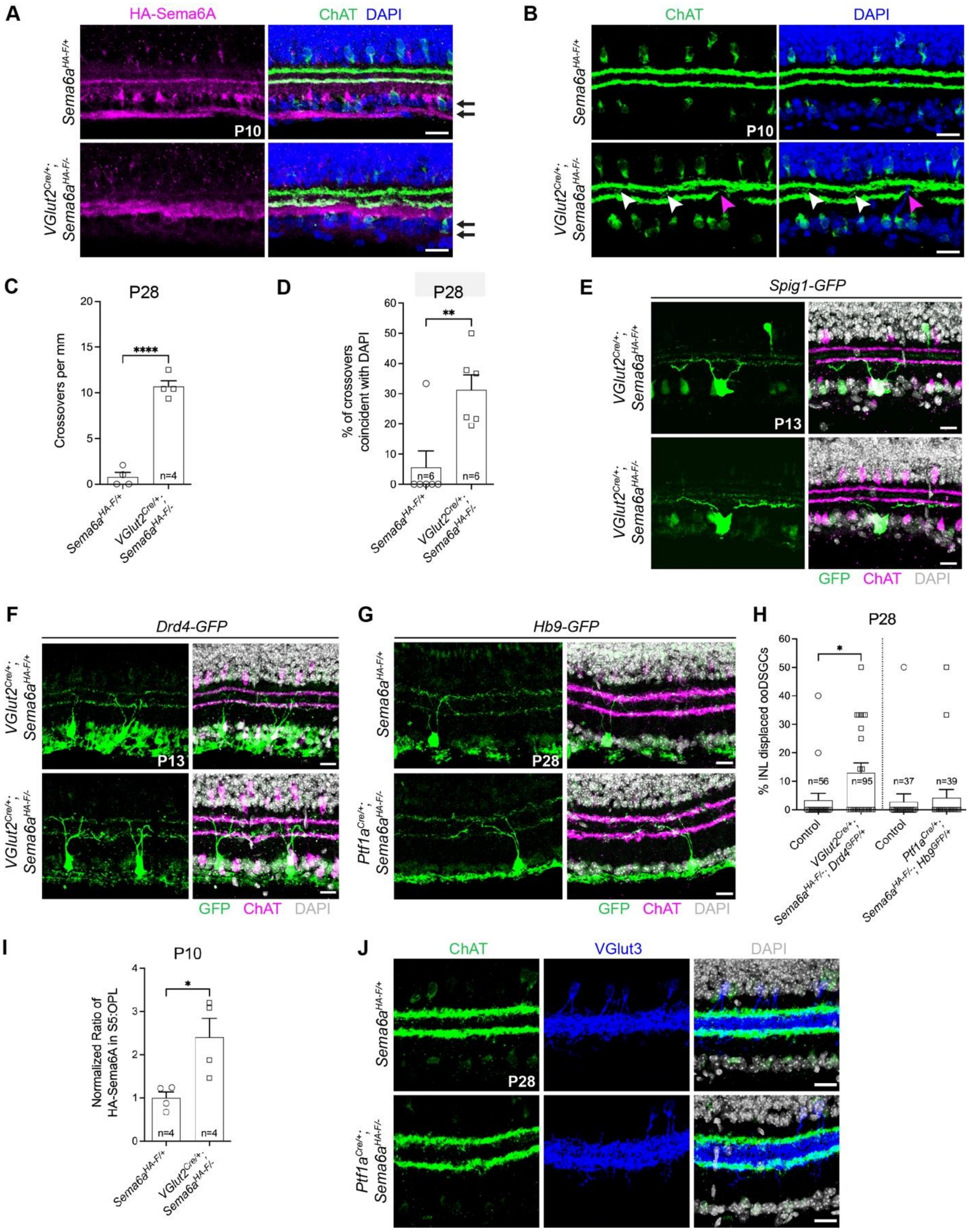

Fig. S12. Sema6A in RGCs promotes SAC dendritic stratification. (**A**) Representative image of a P10 *VGlut2*$^{Cre/+}$; *Sema6a*$^{HA-F/-}$ cKO retinal cross section demonstrating a complete loss of Sema6A in the GCL (upper black arrows) and nerve fiber layer (bottom black arrows) compared

to *Sema6a*$^{HA\text{-}F/+}$, validating the loss of Sema6A in cKO RGCs. **(B)** Representative image of crossovers between S2 and S4 in the IPL of P10 *VGlut2*$^{Cre/+}$; *Sema6a*$^{HA\text{-}F/-}$ cKO retinas (bottom, white arrowheads) compared to *Sema6a*$^{HA\text{-}F/+}$ control retinas. Magenta arrow corresponds to a crossover associated with a DAPI^{+} cell body. **(C)** Quantification of crossovers between S2 and S4 at P10. **(D)** Quantification of the % of crossovers associated with DAPI^{+} cell bodies in the IPL. **(E)** Spig1-GFP^{+} oSDGC lamination is preserved in RGC-specific *Sema6a* cKO retinas. **(F)** Drd4-GFP^{+} ooSDGC lamination is preserved in RGC-specific *Sema6a* cKO retinas. **(G)** Hb9-GFP^{+} ooSDGC lamination is preserved in AC-specific *Sema6a* cKO retinas. **(H)** Quantification of the % of displaced ooDGSCs in retinal cross sections. n, # of ooDSGCs examined. Since differences in ooDSGC displacement were not detected between postnatal timepoints examined, P8 and P13 Drd4-GFP^{+} ooDSGCs were pooled from RGC-specific (*VGlut2*^{Cre}), and P8 and P28 Hb9-GFP^{+} ooDSGCs were pooled from AC-specific (*Ptf1a*^{Cre}), cKO experiments. **(I)** Quantification of the ratio of HA-Sema6A expression in S5 of the IPL compared to the OPL at P10. HA-Sema6A is upregulated in *VGlut2*$^{Cre/+}$; *Sema6a*$^{HA\text{-}F/-}$ cKO retinas. **(J)** Representative image of SAC dendrite stratification in pan-AC (*Ptf1a*^{Cre}) *Sema6a* cKO retinas. Lamination is not altered by loss of Sema6A in all ACs. VGlut3^{+} AC dendritic targeting does not rely on Sema6A in ACs. n (C-D, I), # of retinas. *, $p<0.05$; **, $p<0.01$; ****, $p<0.0001$; Student's t-test or one-way ANOVA, Tukey's MCT. Scale bars, 20µm.

Development • Supplementary information

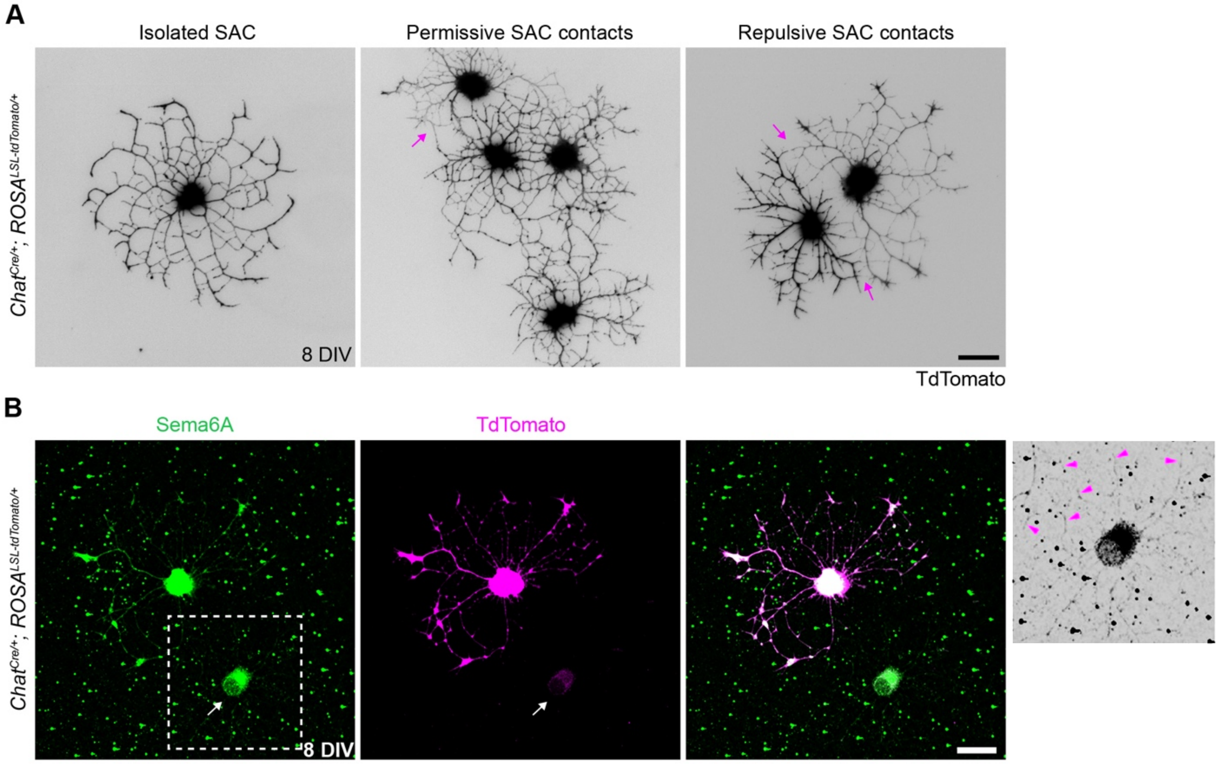

Fig. S13. SAC morphology *in vitro*. (**A**) Representative tdTomato^{+} FACS sorted SACs isolated from *Chat$^{Cre/+}$; ROSA$^{LSLtdTomato/+}$* retinas at P1 and cultured for 8 days *in vitro* (DIV). In pure SAC cultures, isolated SACs adopt a radial morphology (left). SACs experience both permissive contacts, presumably when SACs of the same subtype contact, that do not prevent arbor elaboration (middle), and repulsive contacts that prevent elaboration of the dendritic arbor of one or both contacting SACs (right). Magenta arrows indicate regions where SAC arbors did not elaborate to adopt a radial morphology. **(B)** Representative anti-Sema6A^{+}/tdTomato^{+} SAC in a primary retinal culture. SAC arbors do not elaborate when they contact non-SAC neurons/neurites. White arrows indicate a Sema6A^{+}/tdTomato^{-} non-SAC neuron. The dendritic arbor of the tdTomato^{+} SAC did not elaborate in regions occupied by neurites of the non-SAC neuron. Magenta arrowheads in inset show weakly Sema6A^{+} neurites of the non-SAC neuron.

Development • Supplementary information

Table S1. Key resources

Reagent Type	Designation	Source or reference	Identifier	Additional Information
Antibody	Sema6A; goat, 1:200	R&D Systems	AF1615	
Antibody	HA; rabbit, 1:500	Cell Signaling	C29F4	
Antibody	ChAT; goat, 1:250	EMD-Millipore	AB144P-200µL	SACs
Antibody	NK3R; rabbit, 1:3000	NovusBio	NB300-102	NK3R/TACR3/Neurokinin B Receptor; type 1/2 BCs
Antibody	Calretinin; goat, 1:2500	Swant	CG1	RGCs, ACs
Antibody	DsRed; rabbit, 1:1000	Clontech	632496	
Antibody	RBPMS; guinea pig, 1:500	PhosphoSolutions	1832	RGCs
Antibody	VAChT; goat, 1:1000	EMD-Millipore	ABN100	SACs
Antibody	PKCα; rabbit, 1:1000	Sigma Aldrich	P4334	Rod BCs
Antibody	TH; sheep, 1:400	EMD-Millipore	AB1542	Dopaminergic ACs
Antibody	Syt2; mouse, 1:2000	DSHB	ZNP-1	Type 2/6 CBCs
Antibody	VGlut3; guinea pig, 1:2500	Synaptic Systems	135 204	S2/S3 ACs
Antibody	HCN4; rabbit, 1:500	Alomone Labs	APC-052	OFF CBC type 3a
Antibody	VGlut1; guinea pig, 1:1000	EMD-Millipore	AB5905	BC axons
Antibody	Calbindin; rabbit, 1:2500	Swant	CB-38a	RGCs, ACs
Antibody	GFP; chicken, 1:1000	AVES	GFP-1020	
Antibody	PlexA2; rabbit, 1:500	Generous gift from F. Suto		Murine SACs; IPL layers S2 and S4
Chemical compound	TO-PRO®-3 iodide	Invitrogen	T3605	
Chemical	NeuroTrace® 640⁄660	Life	N-21483	

Development • Supplementary information

compound	Deep-Red Fluorescent Nissl Stain	Technologies		
Chemical compound	DAPI	Life Technologies	D1306	
Chemical compound	Tamoxifen	Sigma Aldrich	T5648-1G	
Chemical compound	Ponceau S Staining Solution	ThermoFisher	A40000278	
Chemical compound	Papain Suspension	Worthington Biochem	LK003176	
Chemical compound	Poly-D-Lysine Hydrobromide	Sigma Aldrich	P1399-25mg	
Chemical Compound	Betaine	Sigma Aldrich	61962-50g	
Biological sample (AAV)	AAV2-FLEX-GFP	UNC Vector Core	100µL: AAV2-CAG-FLEX-GFP	
Biological sample	CTB-555	Life Technologies	C-34776	Cholera Toxin Subunit B (Recombinant), Alexa Fluor® 555 Conjugate
Surgical tool	Silver Nitrate Applicator, 6”	MedVet	RXSNA6	
**Key Smart-Seq2 Reagents**				
	Hibernate A without Ca^{2+}, Mg^{2+}, and Phenol Red	Brainbits	Custom order	
	SMARTScribe™ Reverse Transcriptase	Clontech	639536	
	KAPA HiFi HotStart ReadyMix (100 rxn)	KAPA Biosystems	KK2601	
	Advantage® UltraPure	Clontech	639125	

Development • Supplementary information

	PCR Deoxynucleotide Mix (10 mM each dNTP)			
	Nextera XT V2 DNA Library Prep Kit	Illumina	FC-131-1096	
	Nextera XT Index Kit	Illumina	FC-131-1002	
	Agencourt AMPure XP beads	Beckman Coulter	A63881	
	Qubit® dsDNA HS Assay Kit (500 reactions)	ThermoFisher	Q32854	
