# Supplementary material for: Retinal ganglion cell-derived semaphorin 6A segregates starburst amacrine cell dendritic scaffolds to organize the mouse inner retina
Source: Development. 2024 Nov 26;151(22):dev204293. doi: 10.1242/dev.204293 (PMC11634039; doi:10.1242/dev.204293)
Supplement: Supplementary file 1 [file develop-151-204293_review_history.pdf]

Retinal ganglion cell-derived semaphorin 6A segregates starburst amacrine cell dendritic scaffolds to organize the inner retina

Alex Kolodkin, Rebecca E. James, Natalie R. Hamilton, Matthew P. Brown, Lola Nicole Huffman, Victoria N. Neckles, R. Jeroen Pasterkamp and Loyal A. Goff
DOI: 10.1242/dev.204293

Editor: Steve Wilson

Review timeline

Original submission:	21 November 2023
Editorial decision:	4 January 2024
Resubmission:	31 July 2024
Editorial decision:	20 September 2024
First revision received:	14 October 2024
Accepted:	21 October 2024

Original submission

First decision letter

MS ID#: DEVELOP/2023/202552

I have now received all the referees' reports on the above manuscript, and have reached a decision. The referees' comments are appended below, or you can access them online: please go to BenchPress and click on the 'Manuscripts with Decisions' queue in the Author Area.

As you will see, the referees express considerable interest in your work, but have some criticisms and quite a lot of suggestions for improvement. I think that most of the suggestions are constructive but a few, particularly from reviewer 2, would involve a great deal of extra experimental work (live imaging, generating new genetic lines) that, unless already underway/done, are unlikely to be feasible within a reasonable time for revision. In these cases, you may wish to focus on ensuring that the related data that you do present is as robust as possible and conclusions drawn from such data suitably nuanced. If you are able to revise the manuscript along the lines suggested, I will be happy receive a revised version of the manuscript. Please also note that Development will normally permit only one round of major revision. If it would be helpful, you are welcome to contact us to discuss your revision in greater detail. Please send us a point-by-point response indicating your plans for addressing the referees' comments, and we will look over this and provide further guidance.

Previous reviews

Reviewer 1 Advance Summary and Potential Significance to Field:

In this article James et al., investigate the cell-specific role of Semaphorin (Sema) 6A in regulating neurite lamination in the inner mammalian retina. Using an impressive array of transgenic lines, the authors perturb Sema6a signaling (forward and reverse) and expression in a cell type specific manner and uncover that Sema6a expression in ganglion cells is important for directing dendritic organization of the amacrine cell type central to the direction-selective (DS) circuitry – the starburst amacrine cell (SAC). The authors also uncover that Sema6a expression in the SAC regulates distal dendritic avoidance and cell outgrowth. The study is exhaustive in terms of its implementation of several transgenic lines coupled with viral expression of reporters to study individual SACs. Single cell sequencing is also used to compare gene expression profiles across SAC subtypes and comparisons are made across multiple developmental time points which together

© 2024. Published by The Company of Biologists under the terms of the Creative Commons Attribution License (https://creativecommons.org/licenses/by/4.0/).

enable an understanding of the role(s) of Sema6a during establishment of the retinal circuitry. The study provides new insights into the cell type specific roles of Sema6a for neurite organization in the inner retina. The findings will be of great interest to readers from the neural development and retinal neurobiology fields.

Reviewer 1 Comments for the Author:
I have the following concerns for the authors to address:

i. In relation to the crucial role of the SACs in the DS circuit, and the expression of Sema6a across the DS circuit (in the SACs and DS RGCs), does the lamination and dendritic organization of the DS RGCs (ooDS and/or oDS) get perturbed in the mutants where SACs show dendritic collapse and mislamination? This together with an assessment of the synaptic connectivity between SAC and DS would be necessary for an understanding of how the functional organization of the retina is impacted. Currently the title of the paper states a role for Sema6a in the 'functional specialization' of the retina, but the data included currently is regarding SAC dendritic organization. It can thus not speak to the functioning of the DS circuit and as such the title is not accurate in relation to the current data included. The authors can include synaptic assessments or functional data in terms of the DS circuit to make the connection that Sema6a regulates retinal function. Of note synapses across SAC dendrites are distributed such that the output GABAergic synapses (key for generating DS responses in DS RGCs) are positioned at the outer third of the dendritic arbor. Given that loss of Sema6a signaling in SACs leads to distal dendritic collapse it would be of interest to determine how the SAC synaptic distribution is altered when it is missing its dendritic quadrant or when the distal dendrites collapse as a result of a failure of self-avoidance. The least the authors can do is infer upon the probable synaptic and associated functional deficits across the different mutants with SAC dendritic perturbations – consideration of the ooDS and oDS in this regard would be helpful.

ii. In relation to the Sema6a creER knock-in line, the authors state that 70% of RGCs express Sema6a in this line but it is not clear which RGCs types express Sema6a. This has to be more than the DS RGC population if the stated amount is 70%. The HA labeling as shown in Fig 8A shows the 2 DS bands in the inner retina prominently expressing Sema6a and Fig 6 is centered on the DS RGCs expressing Sema6a in the HA line so it is not obvious which other non-DS RGC types express Sema6a. Along the same thread, the authors state that Sema6a expression in SACs is gone by P14 in the creER line but the HA line shows continued expression corresponding to the 2 ChaT bands even till P28 as shown in Fig 8A. The authors should clarify these differences. Further in relation to the Sema6a expression in DS RGCs (Fig 6), it would be helpful to quantify the fraction of Sema6a positive cells for each DS RGC subtype and for the DS RGC population as a whole and relative to the total RGC population (assessable by RBPMS labeling).

iii. What is the identity of the displaced RGCs when Sema6a signaling is disrupted? In the discussion section, the authors infer that this could specifically be later born RGCs. Whether DS-RGCs are part of this group would be helpful to know and can be ascertained by use of DS RGC specific markers. Also is it the same type of RGCs displaced in the global Sema6a deletion mutants compared to the conditional mutants which show about ½ of the displaced RGC amount? It would be helpful to know the types of RGCs that get displaced in addition to the amount of displacement.

iv. Dendritic collapse errors across mutants: can this be quantified relative to the entire dendritic arbor such that it can be expressed as a % (fraction) of the entire arbor and compared across all the different genotypes. This will help the reader gather whether the error is more pronounced in a specific genotype. The metric of radial symmetry does not encapsulate this entirely as it does not yield the % or extent of the entire dendrite that has collapsed and is thus not available for connecting with synaptic partners. Also, for the mutants where the SACs seem to lose a dendritic quadrant is the loss always the same region/quadrant of the dendritic arbor?

© 2024. Published by The Company of Biologists under the terms of the Creative Commons Attribution License (https://creativecommons.org/licenses/by/4.0/).

v. Fig 1: Please include quantifications from the Megf10cre (shown in 1G) in the 1D plot. Also, this metric of the occurrence of the parallel ChAT bands should be performed for the RGC specific (Vglut2) and RGC+AC (Vglut2+ Ptf1a) Sema6a mutants.

vi. In the Sema6a global KO there seems to be an error in the migration of SAC somas as seen in Fig 5C- the ON and OFF SAC somas seem to be misplaced in the IPL. The authors need to quantify this deficit in this mutant and in the SAC-specific, RGC-specific, and RGC+AC Sema6a mutant to determine whether Sema6a signaling in the AC or RGC fraction regulates this deficit.

vii. In the global Sema6a mutant (Fig 5) quantification of the deficits in bipolar cell and amacrine cell lamination should also be included. The included images of the different markers should be complemented with the corresponding quantification.

viii. Fig 2: Please include P42 quantifications for the OFF SACs as well.

ix. Fig 3C: why not include P10 quantifications in this graph as the images in the remaining figure panels are all from P10.

x. Given that Sema6a signaling in RGCs regulates the dendritic organization of the ON but not OFF SACs, a discussion of why the role of Sema6a is restricted to the ON SAC circuit in terms of possible Sema6a analogs for the OFF SAC circuit would be helpful.

xi. For the plexin independent signaling of Sema6a for organizing dendrites of retinal neurons please expand discussion on which possible pathways these could be.

xii. The quantifications presented are from 2 animals as stated in the methods section and in general for mouse transgenic studies an N of at least 3 animals is generally preferred for rigor and reproducibility. The authors also did not include a power analyses to confirm that a N of 2 animals would be sufficient to reliably capture the phenotypes across the mutants. I would suggest additional analyses for the quantifications where N of 2 animals have been used to increase the sample size to at least 3 animals and an inclusion of the cell numbers (n) and animal numbers (N) in the figure legends for each quantification graph so there is no ambiguity as to the sample size used for the quantifications.

<u>Minor concerns:</u>

i. Fig 1C: what timepoint is this data from. Fig 1D please include the timepoint used for the quantifications.

ii. Fig 3D: the legend states that this violin plot shows expression levels across multiple timepoints, but this is not clear in the included plot.

iii. Suppl Fig 8E: please include labels here regarding what each color channel represents.

iv. In plots depicting “Total RGCs” - does this represent the RGCs in the GCL only or are the displaced RGCs in the INL included as well? Please include clarification.

v. Fig S9B: legend states white arrows but none are included in the figure panel.

vi. Fig S6H-J – why is there a P16 distribution under ‘SAC type’. P16 is already included in the timeline axis and so it is unclear what the P16 red dots represent. Please clarify.

vii. Fig S5D: It is not clear what P14 assessed at P14 represents specially since these data points are at the axis. Please clarify or amend.

viii. Fig 8E: there are always some RGCs that are displaced in the INL but in the schematic no displaced RGC is shown in the INL for WT. Please clarify or amend.

© 2024. Published by The Company of Biologists under the terms of the Creative Commons Attribution License (https://creativecommons.org/licenses/by/4.0/).

ix. Fig 7: Some figure panels use the label cyto instead of cyt for presumably the same Sema6a cytoplasmic mutant – please amend to a single nomenclature so it is not confusing for the readers.

x. Page 14: please specify timepoint of ‘aging’ retina.

xi. Page 23 methods section: typo ‘awas’ instead of was; ‘meg10’ instead of megf10

Reviewer 2 Advance Summary and Potential Significance to Field:
The authors provide a detailed and comprehensive characterization of the roles of Semaphorin 6A, an axon guidance cue, in the development of the mouse retina. They employ advanced genetic approaches to dissect the roles of various sources of Semaphorin 6A in vivo. The authors propose that Semaphorin 6A in starburst amacrine cells (SACs) regulate dendritic arborization in a cell-autonomous manner, while Semaphorin 6A in RGCs regulates the stratification of SAC dendrites. The study has the potential to enhance our understanding of how the inner plexiform layer is formed, adding a new layer to the knowledge of how neuropil formation is regulated. That being said, additional experiments are required to substantiate their main conclusion that RGCs regulate IPL stratification via Semaphorin 6A. Importantly, it is not clear whether the stratification defects observed are caused by the lack of Semaphorin 6A in the RGC population or are secondary to the mispositioning of the RGCs observed in this condition. Moreover, significant concerns regarding the manuscript's clarity, the precision of conclusions stated, and the experimental designs must be addressed prior to consideration for publication.

Reviewer 2 Comments for the Author:
Major comments
1- At times, the manuscript's writing style is difficult to understand. The manuscript's narrative would benefit from a more cohesive structure and focused approach. The connections between paragraphs and sections are somewhat loose. For example, page 4, paragraph 3, the authors abruptly transition from introducing their new mouse line, which provides “a robust avenue for analysis of Sema6a protein distribution”, to deleting the Sema6a gene in different populations without clarifying its expression pattern, which is only addressed in Figure 3. Similarly, on page 8, paragraph 2, the authors introduce a hypothesis about other cell types regulating SAC dendritic stratification via Sema6a. However, this is not clearly connected to the subsequent section on the role of PlexA2.
The authors should consider reorganizing the data presentation to improve flow and facilitate interpretation. Reducing the number of conclusions drawn from minor experiments would aid in clarifying the overall narrative. Moreover, the introduction would be more impactful with a more explicit statement of the knowledge gap and novelty. It might be improved by emphasizing essential points related to the main findings. For instance, the lack of reference to prior research on RGCs regulating IPL formation (Development (2004) 131 (6): 1331–1342) is a missed opportunity, as this is relevant to their primary discovery.
Addressing these issues is important to enable a thorough assessment of the extent to which the data supports the conclusions and would enhance the impact of the manuscript.

2- The authors draw conclusions regarding defects in dendritic outgrowth and self-avoidance based solely on fixed morphological data. For example, from the data presented in Figures 2A, 2E and S3B, the authors infer that SACs exhibit these defects. Could the morphological changes observed be caused by other types of defects, such as dendrite degeneration? Both dendritic outgrowth and self-avoidance are dynamic processes, ideally requiring observation of live cells over time to be accurately assessed. The authors should revise their interpretation to more closely align with what can be conclusively inferred from morphological data, or conduct additional experiments that directly assess growth and self-avoidance. Nonetheless, to substantiate their conclusions throughout the manuscript regarding dendrite self-avoidance errors, the authors should at a minimum quantify the dendrite crossovers.

3- The authors state that inactivation of PlexA2 signalling causes large GAPs on the ON-SAC dendritic plexus (Figure 4E). However, the images presented are variable and no quantification is provided. Employing thresholding of the ChAT signal and measuring the size of areas lacking signal may offer a reliable approach to substantiate this claim.

© 2024. Published by The Company of Biologists under the terms of the Creative Commons Attribution License (https://creativecommons.org/licenses/by/4.0/).

4- Given the conclusion that Sema6a is haploinsufficient, the rationale behind using heterozygous animals as controls in the experiments shown in Figure 5 is unclear. Similarly, in the experiments presented in Figures 7 and 8, the null allele is used alongside the conditional allele to study the function of Sema6a provided by different cellular populations. In conditions like VGlut2Cre; Sema6aHA-F/-, all the cells possess have half the amount of Seman6a compared to the control, while specific populations expressing active Cre recombinase are fully depleted of it. How do the authors distinguish between the effects caused by haploinsufficiency and those specific to the cell type-targeted depletion as well as potential additive effects?

5- The authors report that "Only 12% of RGCs are displaced in VGlut2Cre cKO retinas, yet 25% of total RGCs are displaced in Sema6a-/- mutants". It is unclear whether the observed differences are due to specific genetic conditions or the developmental stages at which the assessments were made. The RGC mispositioning in RGC cKO was evaluated at P28, while in Sema6a-/- mutants it was assessed at P35 and at adult stages. To determine whether whole retina deletion and RGC-specific deletion have different impacts on RGC mispositioning, the two genetic conditions need to be compared at the same developmental stage.

6- The authors propose that Sema6a derived from RGCs regulates SAC plexus organization and SAC dendritic stratification based on results of conditional deletion of Sema6a in RGCs. Although this is an intriguing hypothesis, the experiments need to be expanded to substantiate their claims. The authors show that Sema6a cKO in RGCs also affects RGC positioning. This raises the question of whether the IPL disorganization is directly caused by deletion of Sema6a or a secondary consequence of the presence of RGCs at ectopic locations. Previous work referenced by the authors (Icha et al., 2016) show that ectopic RGCs affect subsequent neuronal lamination events. Being misplaced, RGCs could potentially misguide other neurons or even create physical barriers, contributing to the disorganization of the IPL. This hypothesis is in line with their observation that cKO in RGCs and ACs shows more severe IPL segregation defects as well as higher number of ectopic RGCs. The authors need to isolate the effects of Sema6a deletion in RGCs from the confounding factor of RGC misplacement. Have the authors evaluated if there is spatial correlation between ectopic RGCs and the observed crossovers at P10 (Figure S11B)? Could the authors delete Sema6a in the RGCs after they reached their correct position using a CreER that is specifically expressed in RGCs? Alternatively, could the authors perform rescue experiments by expressing Sema6a in the RGCs via intravitreal viral injection? If the phenotype is rescued even in the presence of ectopic RGCs, it would strongly support the notion that the loss of Sema6a is the primary cause of the defects in IPL organization.

7- The author's observation of large gaps in the plexus following targeted deletion of Sema6a in RGCs is a key finding of the study. As mentioned in point 6, the exact cause of this phenotype is still not clear. A more detailed characterization of how these gaps form is needed. It would be particularly informative to investigate whether SAC arborization is affected in this condition. One possible cause for large gaps in the plexus might be defects in SAC symmetry.
Additionally, live imaging would help determine the temporal relationship between the gap formation and the appearance of ectopic RGCs. This strategy could help discern between cause and consequence, clarifying the hierarchy of these events.

8- It is unclear why the authors assert a significant role for Sema6a provided by RGCs on IPL stratification, given the relatively mild phenotype observed in the conditional knockout as shown in Figure 8A. While the authors suggest compensation by amacrine cells in this condition, this hypothesis is not substantiated with sufficient evidence. Additional data supporting this compensatory mechanism would strengthen the authors' claims regarding the relevance of Sema6a from RGCs. Moreover, how do the authors reconcile the evidence for compensation in IPL stratification with the apparent lack compensation in plexus organization?

9- The authors should include detailed information about sample sizes and statistical analyses in the figure legends. This is crucial not only for experiments quantified but especially for those with qualitative evaluations, such as those depicted in Figures 5 and S8. In qualitative experiments where minor defects in IPL organization are observed, how did the authors exclude potential artefacts introduced by tissue sectioning? This concern is of relevance for the key experiments

© 2024. Published by The Company of Biologists under the terms of the Creative Commons Attribution License (https://creativecommons.org/licenses/by/4.0/).

shown in Figure 8. Crossover needs to be quantified in all the conditions depicted in this figure to substantiate their conclusions.

Minor comments
1- The authors use scRNA-seq to validate the expression pattern of Sema6a in developing SACs. Although this dataset is an important resource for the community, it is unclear what it adds to the already detailed expression data presented in Figures 3 and S5. Moreover, the presentation of the scRNA-seq data does not effectively illustrate the expression pattern. The authors should streamline the description and the visual representation of this data.

2- In Figure 1C, the germline deletion is termed Sox2Cre; Sema6aHA-F whereas in Figure 1E the null allele originating from a germline deletion is referred as Sema6a-/-. Given the complexity of the experiments, including schematics of the alleles and Cre recombination would greatly aid interpretation of the data, helping clarify the difference between the approaches used in S3B and S3C.

3- The authors claim that the Sema6aHA-F allele produces a “fully functional Sema6a protein”. It's important to note that the authors did not directly assess the functionality of the Sema6a protein at the molecular level. Instead, they should accurately describe what was observed in their control experiments, such as the absence of noticeable differences in the process of interest.

4- The authors concluded that “the Sema6aLacZ allele is hypomorphic”, basing this on phenotypic differences between Sema6aLacZ and Sema6aHA-F. However, they do not perform direct comparisons in the current study. Comparisons and conclusions involving other studies should be relegated to discussion section. This is a recurring issue throughout the manuscript.

5- What distinguishes the experiments shown in Figures 2A and S3B? The authors note that in S3B: “The AAV reporter was unable to penetrate deeply enough to label sparse OFF-SACs, precluding quantification of OFF- SAC phenotypes.” Does the approach used in Figure 2A not provide the necessary information to overcome this limitation? A clarification on how these two figures complement each other or provide different insights is needed.

6- The authors claim that “Sema6A mRNA is expressed by postmitotic RGCs in the inner neuroblastic layer at E14.5”. However, no RGC marker was used in the experiment to identify RGCs. Moreover, it is unclear how the authors define which cells are the SACs. The authors need to refine their conclusions to accurately reflect what was observed in their experiments. This issue of imprecision of the conclusions is a recurring concern throughout the manuscript and needs to be systematically addressed to enhance credibility of their findings.

7- The authors discuss their findings in relation to a previous zebrafish study on RGC migration (Icha et al., 2016). They claim that RGC migration is preserved in Sema6A knockout and only fine-positioning is affected. They state: “When basal RGC migration is impaired, RGCs stall near the apical retina surface. Here, basal RGC translocation was entirely preserved.” This statement raises several concerns. Without directly assessing RGC migration in their study, the basis for this conclusion remains unclear. Icha et al., 2016 demonstrated that interference with RGC migration causes RGCs to halt at various positions, including at positions near the RGC layer. The presence of RGCs at the INL in the Sema6A knockout could indicate a delay in their migration, as RGCs might not be able to cross the emerging IPL, as shown for horizontal cells in zebrafish (see https://doi.org/10.7554/eLife.76408). Additionally, extrapolating findings from zebrafish to mice is problematic, it is not clear whether RGCs in mice undergo the same migration phases. The authors should thoroughly review the manuscript to correct similar issues of imprecision in their statements.

8- The manuscript dedicates an entire section of the results to discussing the data presented in Figure S8. The authors should consider condensing the description and integrating it with other relevant sections for a more streamlined presentation.

Reviewer 3 Advance Summary and Potential Significance to Field:

© 2024. Published by The Company of Biologists under the terms of the Creative Commons Attribution License (https://creativecommons.org/licenses/by/4.0/).

In this manuscript, authors James et al. revisit the role of Sema6A signaling in mouse retinal direction-sensitive circuit formation. With several newly generated Sema6a alleles, including the first true null allele, the authors delineate how Sema6A influences multiple aspects of inner retinal development. Through methodical definition of the expression and function of Sema6A and its receptor PlexA2, they make several interesting findings including:

- Sema6A has general roles in organizing the ON sublayer of the IPL synaptic neuropil. This repulsive molecule is strongly enriched in the ON IPL, and when Sema6a is absent, many types of retinal neurons that normally avoid ON IPL begin to innervate these IPL regions.

- Laminar targeting of starburst amacrine cells (SACs) within the IPL is controlled by Sema6a expression in RGCs. This cell nonautonomous function is surprising based on previous findings from the Kolodkin lab. However, Sema6A acts cell autonomously to control SAC dendrite self-avoidance.

- Sema6A is required to confine retinal ganglion cells to the GCL. In Sema6a null mutants, about 30% of RGCs become displaced to the INL.

- Forward signaling through the receptor PlexA2 is required for SAC arbor patterning, but reverse signaling through the Sema6A intracellular domain is also involved in SAC IPL projections and RGC soma placement.

In addition to these findings, the authors also provide a single-cell RNA-seq dataset of OFF and ON SACs across development which will be an important resource for the field. Altogether, this work is very carefully done with impressive genetic tools, and is likely to be of substantial interest to developmental neurobiologists as it advances our understanding of retinal circuit development as well as the function of the important guidance molecule Sema6a.

<u>Reviewer 3 Comments for the Author:</u>
There are some areas in which the paper could be improved:

1. The authors imply in the results (p.13) and discussion (p. 17, second paragraph) that gaps in the ON SAC plexus are caused by RGC misplacement. However, there is insufficient evidence provided that misplaced RGCs align preferentially with the gaps. An alternative interpretation is that the gaps are related to SAC dendrite morphogenesis phenotypes (as shown in Fig. 2A and Fig. S3A-B). The second possibility is perhaps the more likely one, given the relationship of dendrites to holes shown in Fig. S3B, and given that other mutants with defects in SAC dendrite arrangement (e.g. Pcdhg and Megf10 mutants) also exhibit SAC plexus holes (without any RGC misplacement). If the authors wish to keep these passages as currently written, they should provide quantitative evidence that misplaced RGCs are selectively associated with SAC plexus holes.

2. p.15: I am not aware of any paper confirming that Ptf1a-Cre efficiently drives recombination in SACs. This confirmation is needed for the conclusion of Fig. 8A, bottom - "Removing Sema6a from all ACs (Ptf1aCre) highlights the robust expression of Sema6A in DSGC dendrites." The authors should assess Cre reporter expression in SACs to rule out the possibility that Sema6A is expressed by remaining SACs that did not express Cre.

3. p. 17: The authors' model for the RGC positioning phenotype is that there is a defect in radial migration which affects the cell's choice of final location in which to settle. But because only adult timepoints were analyzed, it is also possible that cells migrated normally to the GCL but then later flipped back into the INL. The latter scenario is plausible because Sema6A is localized to the IPL, where it could serve as a repulsive cue that prevents RGCs from moving into the INL during postnatal development. To support their model the authors should show that RGCs are already mislocalized during or shortly after RGC radial migration is complete – i.e. embryonic or early postnatal (e.g. P0) timepoints.

4. The model figure in Fig. 8E was a very nice summary of the complex genetics and resulting phenotypes. However, it needs further explanation so that readers – especially those not deeply immersed in this field – can really appreciate why these are the paper's key conclusions. I would strongly suggest that the authors devote a major part of the Discussion to go through in some detail

© 2024. Published by The Company of Biologists under the terms of the Creative Commons Attribution License (https://creativecommons.org/licenses/by/4.0/).

the evidence supporting the conclusions that are illustrated in this figure. It would make a big difference to the reader's ability to understand how all the disparate findings in the results may be synthesized into a coherent story.

As the discussion stands now, there are a lot of speculative passages about next steps or big picture models – e.g. discussion of ON SAC plexus gaps and whether these are linked to the RGC laminar defects (p. 16, paragraphs 1 & 2); and the discussion of the SAC crossover phenotype (paragraph beginning on the 2nd-to-last line of p. 18). In my view, clarity of the manuscript would be well served by removing these speculative passages and replacing them with a more concrete delineation of the significance of the present findings.

Minor Comments:

Figure 1 could benefit from a model or schematic figure illustrating circuit anatomy -- perhaps incorporating figure S1A and/or S1E into the main figures? Having some visuals would be helpful as a complement to the text within the introduction describing the DS circuits.

p. 4: the sentence starting with: "However, the cell types in which…" is awkward and unclear.

Fig. S6 was very interesting but there were several aspects of figure layout that were confusing. The authors should:
-Flip the color/expression scales so that 0 is at the bottom at 1 is at top on all panels. The current version led to confusion as to which color represents "low" vs. "high" expression.
-Label the clusters more extensively within all panels – e.g. with ages and possibly with text to mark ON and OFF clusters (the asterisks were confusing)
-In panels H-J, it impossible to see the averages, error bars, and colored lines. The lines should be made larger and more noticeable.
-H-J: the blue vs. green color distinction between the ON and OFF groups is hard to discern.
-Legibility of some smaller text could be improved with higher resolution graphics.

For reviewer convenience the authors could use line numbers in their next submission.

P.23: The description of the Sema6a-CreER mouse line should reference Fig. S5A, which has a drawing of the allele structure and features.

P.24, section labeled "SAC Culture": There are references to "Fig. SX" which need to be fixed with the appropriate figure reference.

P.26: The RPM value for centrifuging and pelleting cells is given. Typically, these methods are given in units of RCF. If RPM values are used, the authors should also state the brand/model of centrifuge and rotor that were used.

Author's response to the reviewer's comments

We appreciate the reviewers' interest in our work and their carefully considered comments and suggestions, which have served to strengthen this study. We have considered these points and have tried to address as many as possible, performing new experiments and quantifications where possible, and also including modifications to our presentation.

REVIEWER #1:

We thank the reviewer for their interest in this work, and for their recognition of the comprehensive approaches we have undertaken to better understand and define the roles played by Sema6A in retinal development.

"The study provides new insights into the cell type specific roles of Sema6a for neurite organization in the inner retina. The findings will be of great interest to readers from the neural

© 2024. Published by The Company of Biologists under the terms of the Creative Commons Attribution License (https://creativecommons.org/licenses/by/4.0/).

development and retinal neurobiology fields."

Many insightful and helpful suggestions were provided, and we have addressed this reviewer's concerns as outlined below (**new data** are indicated below with figure citations in "**()**"in **bold,** and with **line numbers** in the revised text that pertain to the issues raised; changes to our original submission in the text of this revision are highlighted in green):

MAJOR POINTS:

(i) *In relation to the crucial role of the SACs in the DS circuit, and the expression of Sema6a across the DS circuit (in the SACs and DS RGCs), does the lamination and dendritic organization of the DS RGCs (ooDS and/or oDS) get perturbed in the mutants where SACs show dendritic collapse and mislamination? This together with an assessment of the synaptic connectivity between SAC and DS would be necessary for an understanding of how the functional organization of the retina is impacted. Currently the title of the paper states a role for Sema6a in the 'functional specialization' of the retina, but the data included currently is regarding SAC dendritic organization. It can thus not speak to the functioning of the DS circuit and as such the title is not accurate in relation to the current data included. The authors can include synaptic assessments or functional data in terms of the DS circuit to make the connection that Sema6a regulates retinal function. Of note synapses across SAC dendrites are distributed such that the output GABAergic synapses (key for generating DS responses in DS RGCs) are positioned at the outer third of the dendritic arbor. Given that loss of Sema6a signaling in SACs leads to distal dendritic collapse it would be of interest to determine how the SAC synaptic distribution is altered when it is missing its dendritic quadrant or when the distal dendrites collapse as a result of a failure of self-avoidance. The least the authors can do is infer upon the probable synaptic and associated functional deficits across the different mutants with SAC dendritic perturbations – consideration of the ooDS and oDS in this regard would be helpful.*

As the reviewer points out, SACs play a crucial role in ordering and structuring the DS circuit. In *Megf10* null mutants, for example, a subset of ooDSGC dendrites track with displaced SAC dendrites in S1 of the IPL (Ray et al., 2018). In this present study we were unable to directly ascertain DSGC dendrite targeting in *Sema6a* null retinas due to the nearly complete fusion of the SAC arbors – we have no way of determining if ON DSGC arbors are displaced to OFF SAC arbors (or vice versa) because we cannot discern ON from OFF SAC arbors with the complete anatomical disruption of the SAC strata in this mutant background. We can, however, provide new data demonstrating targeting of a subset of DSGC dendrites in RGC-specific *VGlut2*^{Cre} and AC-specific *Ptfa1*^{Cre} cKO retinas, providing an examination certain cell-type specific roles of *Sema6a* in DSGC targeting. Unfortunately, we are not able to examine this in double cKO backgrounds since generating quadruple transgenic animals would far exceed the timeline for revision.

Regarding the use of "functional" in the title, our apologies. Our intention underlying "functional specialization" was to refer generally to the role of Sema6A in segregating well-characterized ON versus OFF circuits with discrete functions in light detection. We did not mean to imply that Sema6A is require for all ON versus OFF functional responses. We understand how this could be misleading, and we have changed the title accordingly.

We agree the issue of synapse distribution in *Sema6a* mutant SAC arbors is a fascinating question, and one we invested a lot of effort addressing some time ago in the *Sema6a*^{LacZ} background, before we generated and characterized the *Sema6a* null allele presented in this study. In this hypomorphic background, we initially detected a synapse redistribution phenotype whereby ROSA-LSL-Synaptophysin^{tdTomato}-positive presynaptic specializations were abundantly found within the inner 2/3 of the SAC dendritic arbor, in addition to the outer 1/3 of the arbor. However, after generating the *Sema6a* null allele and repeating this experiment in a SAC-specific *Sema6a* cKO background over the null allele, this phenotype did not repeat. We believe the phenotype originally observed in the cKO background over the *Sema6a*^{LacZ} allele represents a neomorphic phenotype. Synapse distribution was also undisturbed in the *Plxna2* null retinas, supporting the idea that Sema6A-PlexA2 signaling is dispensable in SACs for synapse positioning. We will add these negative data to the supplement.

© 2024. Published by The Company of Biologists under the terms of the Creative Commons Attribution License (https://creativecommons.org/licenses/by/4.0/).

Major Point (i) Action Items:

- **a)** We have provided new data demonstrating that Sema6A is not required in RGCs or ACs for lamination of some DSGCs types **(see: Fig. S12E-G)**
- b) We have modified the title to remove "functional."
- **c)** We have provided new data regarding synapse distribution in SAC-specific *Chat*$^{CreER/+}$*; Sema6a*$^{HA\text{-}F/LacZ}$ vs. *Chat*$^{CreER/+}$*; Sema6a*$^{HA\text{-}F/-}$, and also in *Plxna2*$^{-/-}$, demonstrating that Sema6A-PlexA2 signaling is not required in SACs for synapse distribution. (**see Fig. S3)**

(ii) *In relation to the Sema6a creER knock-in line, the authors state that 70% of RGCs express Sema6a in this line but it is not clear which RGCs types express Sema6a. This has to be more than the DS RGC population if the stated amount is 70%. The HA labeling as shown in Fig 8A shows the 2 DS bands in the inner retina prominently expressing Sema6a and Fig 6 is centered on the DS RGCs expressing Sema6a in the HA line so it is not obvious which other non-DS RGC types express Sema6a. Along the same thread, the authors state that Sema6a expression in SACs is gone by P14 in the creER line but the HA line shows continued expression corresponding to the 2 ChaT bands even till P28 as shown in Fig 8A. The authors should clarify these differences. Further in relation to the Sema6a expression in DS RGCs (Fig 6), it would be helpful to quantify the fraction of Sema6a positive cells for each DS RGC subtype and for the DS RGC population as a whole and relative to the total RGC population*

We agree–Sema6A is broadly expressed by many RGC types early in development. We observe that this expression refines over time such that only DSGCs strongly express Sema6A in mature animals, represented by the robust HA-Sema6A expression overlapping with the ChAT bands in the *Ptf1a* cKO panel as seen in Fig. 8A. Consistently, *Sema6a*^{CreER} reporter activity in the RGC population is dramatically reduced by P14, similar to what we observe in SACs (Fig. S5D). We think that HA-Sema6A expression overlapping with the ChAT bands in P28 *Ptf1a*^{Cre} cKO animals is derived from DSGCs since *Sema6a* is knocked out of amacrine cells (including SACs) in this background. Additionally, we have some evidence that *Sema6A*^{CreER} expression in mature SACs (>P20) is quite sparse – very few SACs are tdTomato+. **See Fig. S5E.**

In relation to Sema6A expression in subsets of DSGCs, we have quantified the fraction of GFP+ DSGCs that were positive for HA-Sema6A for the transgenes assessed in Fig. 6. Unfortunately, however, these genetic backgrounds were not stained for HA-Sema6A and RBMPS, and these strains are no longer available for additional analysis.

Major Point (ii) Action Item(s):

- **a)** We have quantified the % of GFP+ DSGCs (Hb9, Spig1, Drd4, Hoxd10) positive for HA-Sema6A from the P10 images related to Fig. 6. (**see Fig. 6D)**
- **b)** We have enhanced our discussion around Sema6A expression in RGCs over developmental time. **(see lines 431-435)**

(iii) *What is the identity of the displaced RGCs when Sema6a signaling is disrupted? In the discussion section, the authors infer that this could specifically be later born RGCs. Whether DS-RGCs are part of this group would be helpful to know and can be ascertained by use of DS RGC specific markers. Also is it the same type of RGCs displaced in the global Sema6a deletion mutants compared to the conditional mutants which show about ½ of the displaced RGC amount? It would be helpful to know the types of RGCs that get displaced in addition to the amount of displacement.*

We thank the reviewer for this excellent question – we looked at final positioning of subtypes of DSGCs in *VGlut2*^{Cre} (RGC) and *Ptf1a*^{Cre} (AC) cKO backgrounds, including Drd4-GFP and Hb9-GFP ooDSGCs, and Spig1-GFP oDSGCs, but there was no clear indication that a particular subtype was uniquely displaced. Rather, infrequent GFP+ cells from each transgenic background examined were displaced into the IPL / INL. We therefore do not believe the displaced cells correspond to a specific subtype, but rather correspond to a temporal identity: the latest-born RGCs. We have provided data demonstrating the random, infrequent, DSGC displacement.

Major Point (iii) Action Item(s):

- **a)** We have provided data showing that infrequent DSGCs are displaced. **(see Fig. S12H)**

© 2024. Published by The Company of Biologists under the terms of the Creative Commons Attribution License (https://creativecommons.org/licenses/by/4.0/).

b) We have described in the text that Sema6A in RGCs and ACs does not appear to regulate DSGC dendrite targeting in the IPL, related to data mentioned above in (a). **(Fig. S12E-G, line 417-425).**

(iv) *Dendritic collapse errors across mutants: can this be quantified relative to the entire dendritic arbor such that it can be expressed as a % (fraction) of the entire arbor and compared across all the different genotypes. This will help the reader gather whether the error is more pronounced in a specific genotype. The metric of radial symmetry does not encapsulate this entirely as it does not yield the % or extent of the entire dendrite that has collapsed and is thus not available for connecting with synaptic partners. Also, for the mutants where the SACs seem to lose a dendritic quadrant is the loss always the same region/quadrant of the dendritic arbor*

Major Point (iv) Action Items:
We have provided a % arbor collapse, relative to a presumptive 360° arbor, to address this point. Unfortunately, we did not track the orientation of retinas and SACs within these retinas in these studies. **(See % arbor collapse data in Figs. 2E-F; S2C,F; 4C)**

(v) *Fig 1: Please include quantifications from the Megf10cre (shown in 1G) in the 1D plot. Also, this metric of the occurrence of the parallel ChAT bands should be performed for the RGC specific (Vglut2) and RGC+AC (Vglut2+ Ptf1a) Sema6a mutants.*

Major Point (v) Action Items:
We have provided these quantifications. **(See Fig.1E, Fig. 8D)**

(vi) *In the Sema6a global KO there seems to be an error in the migration of SAC somas as seen in Fig 5C- the ON and OFF SAC somas seem to be misplaced in the IPL. The authors need to quantify this deficit in this mutant and in the SAC-specific, RGC- specific, and RGC+AC Sema6a mutant to determine whether Sema6a signaling in the AC or RGC fraction regulates this deficit.*

SAC positioning is unaffected in SAC-specific and AC-specific cKO retinas, so we do not think that Sema6A is cell-autonomously required for SAC migration/settling.
Anecdotally, it appears that some ON-SACs are displaced into the IPL in *Sema6a* null retinas, which may be related to *de novo* repulsive interactions with mistargeted retinal neurites or displaced RGCs. However, it is not possible for us with our current approaches to discern ON- from OFF-SACs and their dendrites within the IPL, owing to a lack of antibodies to label and identify ON- versus OFF-SACs. However, we have quantified ON-SACs in the GCL and OFF-SACs the INL in *Sema6a*$^{-/-}$ retinas to tease apart the identity of the displaced SACs.

Major Point (vi) Action Items:

a) We attempted to quantify, to the best or our ability, all SACs displaced to the IPL in *Sema6a* null, *VGlut2*^{Cre} cKO, double cKO, and *Ptf1a*^{Cre} cKO backgrounds.
Unfortunately, reliably detecting SACs within the dense ChAT/VAChT immunolabeling IPL was not feasible.
b) We have quantified ON- and OFF-SACs in *Sema6a* null, *Ptf1a*^{Cre} cKO, *VGlut2*^{Cre} cKO, and *Ptf1a*^{Cre}; *VGlut2*^{Cre} double cKO backgrounds to determine if SACs are appropriately positioned. We quantified the **number of ON- and OFF-SAC somas in the INL and GCL of *Sema6a* null mutants (see Fig. S8A-B, and lines 322-325)**.We found no evidence of OFF-SAC soma displacement in *VGlut2*^{Cre} or *Ptfa1*^{Cre} single, or *Ptf1a*^{Cre}; *VGlut2*^{Cre} double cKO backgrounds, whereas some ON-SACs were displaced in *Ptf1a*^{Cre}; *VGlut2*^{Cre} double cKO backgrounds (**see Fig. S11G-H**).

(vii) *In the global Sema6a mutant (Fig 5) quantification of the deficits in bipolar cell and amacrine cell lamination should also be included. The included images of the different markers should be complemented with the corresponding quantification.*

We have quantified neurites from experiments presented in Fig. 5 that we are able to resolve. We were not able to discern type 2 vs. type 6 cone bipolar cells (Syt2+), given the intermingling of Syt2+ neurites in *Sema6a* null retinas.

© 2024. Published by The Company of Biologists under the terms of the Creative Commons Attribution License (https://creativecommons.org/licenses/by/4.0/).

Major Point (vii) Action Items:

a) We have determined a metric to quantify PKCa and HCN4 bipolar cell immunolabeling. **(See Fig5. E,G)**
b) We have quantified the % of the IPL filled with VGlut3 amacrine cell neurites. **(See Fig. 5F)**

(viii) *Fig 2: Please include P42 quantifications for the OFF-SACs as well.*

Regrettably, we were unable to sufficiently label enough P42 OFFs SACs with the AAV- GFP virus to permit quantification, despite several attempts at this experiment. The issue is that virus penetration into the deep INL is less efficient than the more superficial GCL. When rare OFF-SACs are targeted by reporter viruses, they are usually surrounded by strongly labeled ON-SACs, and the strength of the signal from the ON- SACs precludes imaging and resolving the OFF-SAC dendrites.

(ix) *Fig 3C: why not include P10 quantifications in this graph as the images in the remaining figure panels are all from P10.*

Major Point (ix) Action Item:
Regrettably, the P10 time point data were collected at a higher magnification for the purposes of image acquisition, not for quantification. The number of images collected/ROIs sampled was not standardized to cover the entire retina, nor were they consistent with quantifications at earlier time points, precluding inclusion of a P10 quantification.

(x) *Given that Sema6a signaling in RGCs regulates the dendritic organization of the ON- but not OFF-SACs, a discussion of why the role of Sema6a is restricted to the ON SAC circuit in terms of possible Sema6a analogs for the OFF SAC circuit would be helpful.*

While we cannot rule out an instructive role for RGC-derived Sema6A in ON SAC plexus organization, we think it is helpful to consider the select disruption of the ON SAC plexus in *VGlut2*^{Cre} cKO backgrounds as a secondary consequence of neuron migration and neurite targeting defects. In this light, there is no function that requires an analog for the OFF-SAC circuit *per se*, because the OFF-SAC plexus is likely not exposed to the same environment that is disrupting the ON plexus (i.e. specific neurites that are mistargeted in *VGlut2*^{Cre} cKO backgrounds). Additionally, our extensive expression analyses reveal that Sema6A is enriched in the GCL and regions of the IPL that are adjacent to the GCL (see Fig. 3A), suggesting that normal functions for RGC- derived Sema6A in ON-SAC plexus organization are likely governed by proximity.

Please see lines 484-502.

(xi) *For the plexin independent signaling of Sema6a for organizing dendrites of retinal neurons please expand discussion on which possible pathways these could be.*

Sema6A has classically been shown to bind to PlexA2 and PlexA4 receptors, but class 6 semaphorins can also bind immune coreceptors, including TREM2 and DAP12. Roles for neuronal Sema6A interactions with Muller glia or endothelial cells (which also express Sema6A) in neurite lamination have not been examined and would be interesting to pursue in follow-on studies. Additionally, it remains possible that Sema6A– Sema6A binding in *trans* could facilitate neurite targeting for some populations of neurons.

Major Point (xi) Action Item:
We have added additional context in our revised text about possible interactions and pathways that could transduce these plexin-independent Sema6A signaling events. We have modified our Discussion to track the model in Fig. 8F more closely, so our discussion of plexin-independent roles for Sema6A is now de-emphasized. However, we reframed **lines 316-322** to focus on the hypothesis that mistargeting arises from disruption of the SAC scaffolds.

(xii) *The quantifications presented are from 2 animals as stated in the methods section and in general for mouse transgenic studies an N of at least 3 animals is generally preferred for rigor and reproducibility. The authors also did not include a power analyses to confirm that a N of 2*

© 2024. Published by The Company of Biologists under the terms of the Creative Commons Attribution License (https://creativecommons.org/licenses/by/4.0/).

animals would be sufficient to reliably capture the phenotypes across the mutants. I would suggest additional analyses for the quantifications where N of 2 animals have been used to increase the sample size to at least 3 animals and an inclusion of the cell numbers (n) and animal numbers (N) in the figure legends for each quantification graph so there is no ambiguity as to the sample size used for the quantifications.

We agree that an n of 3 or more animals would have been ideal, and this was our target goal, but unfortunately due to the complicated genetics used in many of our experiments this was not feasible. For example, we were challenged in generating breeders containing both the $Megf10^{Cre}$ and *Sema6a* null/floxed alleles for many of our crosses, due to their proximity and genetic linkage. Oddly, for some crosses, only ***females*** of a particular genotype would generate pups of the desired genotypes: e.g. only $\mathbf{Megf10}^{Cre/Cre}$; $VGlut2^{Cre/+}$, $Sema6a^{+/-}$ but *not* $\mathbf{Megf10}^{Cre/+}$; $VGlut2^{Cre/+}$, $Sema6a^{+/-}$ females would yield pups that were $Megf10^{Cre/+}$; $VGlut2^{Cre/+}$, $Sema6a^{F/-}$. We generated over a dozen litters from the only two female breeders we obtained (from of many litters) to increase our n from 2 to 3 animals for this double cKO experiment, but we were ultimately unsuccessful and felt it was unethical to continue breeding for this experiment. Given our breeding limitations, we thought it best to quantify only robust phenotypes, and we note that the number of neurons or retinal regions scored in these experiments where the number of mice is limited to 2 is quite large. These considerations have been added to the Materials and Methods sections **(lines 599-603)**

Major Point (xii) Action Item:
We have now described the retina and cell number n's in the legends explicitly (as is done in all legends for quantifications), and as stated above, we have added additional explanations in the Materials and Methods on this point.

Minor Points:

We have addressed all minor concerns as described below.

(i) Fig 1C: what timepoint is this data from. Fig 1D please include the timepoint used for the quantifications.
We have added this to the figure panel as requested.

(ii) Fig 3D: the legend states that this violin plot shows expression levels across multiple timepoints, but this is not clear in the included plot.

We have modified this legend for clarity to indicate that all timepoints are pooled in this violin plot presentation of the data. We have also added a description to the image.

(iii) Suppl Fig 8E: please include labels here regarding what each color channel represents.

Thank you, we must have lost these labels in our editing, and we have now added them back.
(iv) In plots depicting "Total RGCs" - does this represent the RGCs in the GCL only or are the displaced RGCs in the INL included as well? Please include clarification.

All RGCs, including those in the GCL along with those that are displaced, are included in these quantifications. We have clarified this point in the relevant legends and in on **line 809** of the Materials and Methods.

(v) Fig S9B: legend states white arrows but none are included in the figure panel.

Thank you, these were lost in our editing of panel positions, and we have added them these back in.
(vi) Fig S6H-J – why is there a P16 distribution under 'SAC type'. P16 is already included in the timeline axis and so it is unclear what the P16 red dots represent. Please clarify.
This was to qualify the pink dot identity, in line with the green and blue dot definitions, however for clarity we have removed this.

(vii) Fig S5D: It is not clear what P14 assessed at P14 represents specially since these data

© 2024. Published by The Company of Biologists under the terms of the Creative Commons Attribution License (https://creativecommons.org/licenses/by/4.0/).

points are at the axis. Please clarify or amend.
We have added the % of SACs that were tdTomato+ at P14 to the legend.

(viii) Fig 8E: there are always some RGCs that are displaced in the INL but in the schematic no displaced RGC is shown in the INL for WT. Please clarify or amend.

This image was intended to provide a magnified snapshot of the retina, and often the displaced RGCs were spaced far enough apart that they were not captured in a single cross section image (see Figs.5D and S8D). We have now added a displaced RGC to this model to demonstrate that some RGCs are normally displaced.

(ix) Fig 7: Some figure panels use the label cyto instead of cyt for presumably the same Sema6a cytoplasmic mutant – please amend to a single nomenclature so it is not confusing for the readers.

Thank you – we changed our nomenclature just prior to our initial submission to keep in line with Verhagen et al. 2023, and we missed this figure panel. It has now been corrected.

(x) Page 14: please specify timepoint of 'aging' retina.

We have removed this statement from our text for clarity.

(xi) Page 23 methods section: typo 'awas' instead of was; 'meg10' instead of megf10.

Thank you – we have corrected these typographic errors.

REVEIWER #2

We thank this reviewer for their assessment of our study and stating that it is a comprehensive characterization of how Sema6A influences retina development with the potential to provide insight into understanding retina development.

The study has the potential to enhance our understanding of how the inner plexiform layer is formed, adding a new layer to the knowledge of how neuropil formation is regulated Several important issues are raised, however, and we have tried to address them in order to strengthen our study and satisfy these concerns.

Major Comments

1- At times, the manuscript's writing style is difficult to understand. The manuscript's narrative would benefit from a more cohesive structure and focused approach. The connections between paragraphs and sections are somewhat loose. For example, page 4, paragraph 3, the authors abruptly transition from introducing their new mouse line, which provides "a robust avenue for analysis of Sema6a protein distribution", to deleting the Sema6a gene in different populations without clarifying its expression pattern, which is only addressed in Figure 3. Similarly, on page 8, paragraph 2, the authors introduce a hypothesis about other cell types regulating SAC dendritic stratification via Sema6a. However, this is not clearly connected to the subsequent section on the role of PlexA2. The authors should consider reorganizing the data presentation to improve flow and facilitate interpretation. Reducing the number of conclusions drawn from minor experiments would aid in clarifying the overall narrative. Moreover, the introduction would be more impactful with a more explicit statement of the knowledge gap and novelty. It might be improved by emphasizing essential points related to the main findings. For instance, the lack of reference to prior research on RGCs regulating IPL formation (Development (2004) 131 (6): 1331–1342) is a missed opportunity, as this is relevant to their primary discovery. Addressing these issues is important to enable a thorough assessment of the extent to which the data supports the conclusions and would enhance the impact of the manuscript.

Thank you for this feedback. We also agree that omission of (Kay et al., 2004) was a regrettable oversight. We will address your concerns as outlined below.

© 2024. Published by The Company of Biologists under the terms of the Creative Commons Attribution License (https://creativecommons.org/licenses/by/4.0/).

Major Point (1) Action Items:

a) We have restructured the text where feasible to improve flow of the manuscript, reducing the conclusions drawn from minor experiments where relevant. We have removed modified the text accordingly.
b) We have added text regarding (Kay et al., 2004) to the Introduction to better frame the study. **Please see lines 69-71.**

2- The authors draw conclusions regarding defects in dendritic outgrowth and self- avoidance based solely on fixed morphological data. For example, from the data presented in Figures 2A, 2E and S3B, the authors infer that SACs exhibit these defects. Could the morphological changes observed be caused by other types of defects, such as dendrite degeneration? Both dendritic outgrowth and self-avoidance are dynamic processes, ideally requiring observation of live cells over time to be accurately assessed. The authors should revise their interpretation to more closely align with what can be conclusively inferred from morphological data, or conduct additional experiments that directly assess growth and self-avoidance. Nonetheless, to substantiate their conclusions throughout the manuscript regarding dendrite self- avoidance errors, the authors should at a minimum quantify the dendrite crossovers.

We agree that live imaging would be a great way to quantify outgrowth and self- avoidance. Given our extensive characterization of the roles of Sema6A-PlexA2 signaling in these processes in Sun et al., 2013, which included neonatal live imaging, and our expectation that dendrite outgrowth and self-avoidance would be disrupted in the *Sema6a* null background based upon that previous study, we instead chose to focus our efforts on establishing the cell-type specific requirements for *Sema6a* in these and other processes during retinal development. Unfortunately, given the extreme density and clumping of SAC dendrites in the *bona fide Sema6a* null retinas presented here, it was not possible for us to accurately discern individual dendrites for self-crossing quantifications in all cases.

Major Point (2) Action Items:

a) We have modified the text where appropriate to reflect dendrite area vs. dendrite outgrowth. **Please see lines: 132, 134, 136, 137, 144, 150, 154, 242, 245, 473,** 475,
b) In our 2013 study we quantified crossovers in targeted Alexa-555 dye-filled ON- SACs, in which the entirety of the dendrite length is filled with dye, in addition to the boutons. We when attempted to quantify crossovers as part of this revised manuscript, we noticed that tdTomato routinely filled the boutons and distal-most dendrites in our genetically-labeled experiments, but insufficiently filled proximal and intermediate dendrites, so it was difficult to discern signal from noise to identify the dendrite skeleton. This introduced more subjectivity to the quantification than we were comfortable with. Regardless, we have limited our interpretation here to highlight exclusively the existence of self-crossing errors, which we believe is well-exemplified in the images presented that did display robust tdTomato throughout the body of the dendrites. We hope this limitation in interpretation, and the multiple genetic backgrounds demonstrating the same phenotype (see Figs. 2, 4, S2, S3, and S7), satisfactorily addresses this concern.

3- The authors state that inactivation of PlexA2 signalling causes large GAPs on the ON-SAC dendritic plexus (Figure 4E). However, the images presented are variable and no quantification is provided. Employing thresholding of the ChAT signal and measuring the size of areas lacking signal may offer a reliable approach to substantiate this claim.

Quantifying gaps in *PlexA2rasGAP* mutant ON SAC plexuses, we did for Fig. 7 quantifications, is feasible. However, thresholding the ChAT signal and measuring the size of regions lacking signal is complicated by the unevenness of whole mount retinas. It is quite rare to capture a single, uniform, SAC plexus throughout an entire image ROI without signal bleed-through or encountering regions within the image frame that are too deep or too superficial and contain the non-target SAC plexus. This morphological limitation makes thresholding quantifications of single plexuses problematic.

Major Point (3) Action Item:

Unfortunately, we were unable to quantify gaps in the *Plxna2*^{RasGAP} mutant experiment because

© 2024. Published by The Company of Biologists under the terms of the Creative Commons Attribution License (https://creativecommons.org/licenses/by/4.0/).

these images were collected to capture AAV-LSL-GFP labeled SACs. We did not image and collect data from each retinal quadrant at similarly positioned ROIs for gap quantification, as we did in our experiments presented in Fig. 7. We did, however, add quantification of gaps in pan-AC- **(see Fig. 7F)** and SAC-specific **(Fig. 7G)** cKO retinas to further explore this phenotype.

4- Given the conclusion that Sema6a is haploinsufficient, the rationale behind using heterozygous animals as controls in the experiments shown in Figure 5 is unclear. Similarly, in the experiments presented in Figures 7 and 8, the null allele is used alongside the conditional allele to study the function of Sema6a provided by different cellular populations. In conditions like VGlut2$^{Cre/+}$; Sema6a$^{HA\text{-}F/-}$, all the cells possess have half the amount of Seman6a compared to the control, while specific populations expressing active Cre recombinase are fully depleted of it. How do the authors distinguish between the effects caused by haploinsufficiency and those specific to the cell type-targeted depletion as well as potential additive effects?

The experiments in Fig. 5 were conducted prior to our discovery that *Sema6a* is haploinsufficient for some phenotypes. We observed, however, that the P35 *Sema6a*$^{+/-}$ ON SAC plexus was grossly well-organized and was comparable to the P35 wildtype ON SAC plexus, and so we felt confident using the *Sema6a*$^{HA\text{-}F/+}$ and *Sema6a*$^{Cyt/+}$ genetic backgrounds to control plexus gaps for our cKO experiments in Fig.7. In these experiments, we thought that the benefits of controlling for potential contributions of the Cre driver to retinal phenotypes outweighed the risks of subtle contributions of the heterozygous condition in RGCs. Additionally, since we observed displacement of only 6.8% of RGCs in adult *Sema6a*$^{+/-}$ retinas, we anticipated that contributions of haploinsufficiency to RGC displacement would not exceed ~7% in the cKO backgrounds. Accordingly, 2.2% of RGCs were displaced in the *VGlut2*$^{Cre/+}$; *Sema6a*$^{HA\text{-}F/+}$, and 1.5% in the *VGlut2*$^{Cre/+}$; *Sema6a*$^{Cyt/+}$, cKO retinas at P28, whereas only 1.6% of RGCs are displaced in P28 C57BL/6 retinas (Figs. 7C,H; S8I). Together, these data suggest that *Sema6a* haploinsufficiency within RGCs is not sufficient to significantly impact RGC displacement and obscure the effects of the various genetic perturbations presented in Fig. 7.

5- The authors report that “Only 12% of RGCs are displaced in VGlut2Cre cKO retinas, yet 25% of total RGCs are displaced in Sema6a-/- mutants”. It is unclear whether the observed differences are due to specific genetic conditions or the developmental stages at which the assessments were made. The RGC mispositioning in RGC cKO was evaluated at P28, while in Sema6a-/- mutants it was assessed at P35 and at adult stages. To determine whether whole retina deletion and RGC-specific deletion have different impacts on RGC mispositioning, the two genetic conditions need to be compared at the same developmental stage.

RGCs are the earliest born retinal neurons and the first to migrate through the neuroblastic layers to form the nascent ganglion cell layer in the basal retina. Since their specification is complete early postnatally, we think that differences seen in juvenile and adult mice do not reflect a developmental phenotype. Additionally, in our previous studies we have not observed differences when examining several phenotypes – including RGC displacement and SAC lamination: for example when assessing phenotypes in “juvenile” mice (ie., P28-P42). Consistently, only 1% of RGCs are displaced in wildtype (C57BL/6) retinas at P35 (Fig. 5I) and, as mentioned in Point (4) above, 1.6% of RGCs are displaced in wildtype (C57BL/6) retinas at P28 (Fig. S8G).
Thus, we consider these specific time points, P28 and P35, to be a close approximation of a similar developmental stage.

Major Point (5) Action Item:
We examined RGC displacement in neonate *Sema6a* mutant retinas to determine if RGCs are similarly displaced shortly after their specification has ended, likely reflecting an early developmental migration phenotype, versus a progressive migration phenotype in later stages of development. We performed new experiments to assess RGC localization in neonatal retinas, collecting tissue from E18.5-P0.5. We observed stacking of RGCs at the ONBL/IPL boundary, without mislocalization of RGCs in the ONBL, suggesting that either 1) a switch in migratory modes was not achieved, or 2) a novel inhibitory boundary prevents the final steps of migration and settling in the GCL of a subset of RGCs (**see Fig. 5M-N).**

© 2024. Published by The Company of Biologists under the terms of the Creative Commons Attribution License (https://creativecommons.org/licenses/by/4.0/).

6- The authors propose that Sema6a derived from RGCs regulates SAC plexus organization and SAC dendritic stratification based on results of conditional deletion of Sema6a in RGCs. Although this is an intriguing hypothesis, the experiments need to be expanded to substantiate their claims. The authors show that Sema6a cKO in RGCs also affects RGC positioning. This raises the question of whether the IPL disorganization is directly caused by deletion of Sema6a or a secondary consequence of the presence of RGCs at ectopic locations. Previous work referenced by the authors (Ichet [Icha] et al., 2016) show that ectopic RGCs affect subsequent neuronal lamination events. Being misplaced, RGCs could potentially misguide other neurons or even create physical barriers, contributing to the disorganization of the IPL. This hypothesis is in line with their observation that cKO in RGCs and ACs shows more severe IPL segregation defects as well as higher number of ectopic RGCs. The authors need to isolate the effects of Sema6a deletion in RGCs from the confounding factor of RGC misplacement. Have the authors evaluated if there is spatial correlation between ectopic RGCs and the observed crossovers at P10 (Figure S11B)? Could the authors delete Sema6a in the RGCs after they reached their correct position using a CreER that is specifically expressed in RGCs? Alternatively, could the authors perform rescue experiments by expressing Sema6a in the RGCs via intravitreal viral injection? If the phenotype is rescued even in the presence of ectopic RGCs, it would strongly support the notion that the loss of Sema6a is the primary cause of the defects in IPL organization.

We agree it is possible that IPL disorganization in *VGlut2*^{Cre} cKO retinas is a secondary consequence of ectopic neurons/neurites. We observed no obvious correlation between crossovers and RBPMS+ cell bodies in P10 or P28 in *VGlut2*^{Cre} cKO retinas. If crossovers were related to displaced RGCs, we would anticipate a more robust crossover phenotype in the *VGlut2*^{Cre} cKO retinas, something commensurate with ½ of the fusion phenotype, since *VGlut2*^{Cre} cKO retinas exhibit ½ of the displacement phenotype of *Sema6a* null retinas. We have noted that the frequency of SAC crossovers in *VGlut2*^{Cre} cKO retinas is the same as we observe for *Plxna2* null SAC crossovers (PlexA2 is only expressed in SACs), suggesting that a direct Sema6A- PlexA2 signaling pathway between Sema6A in RGCs and PlexA2 in SACs underlies this minor crossover phenotype. The reviewer suggests several interesting experiments, and indeed recently an *RBPMS*^{CreER} driver has been published (Guo et al., 2023), but the time it would take to generate genetic background-matched allele combinations for cKO studies, or to produce Cre-dependent Sema6A viruses, far exceeds the revision timeline for this study.

Major Point (6) Action Items:

a) We have quantified the frequency of coincident crossovers and DAPI+ cell bodies in *VGlut2*^{Cre} cKO retinas. (**see Fig. S12D**).
b) We added new data assessing the possibility that displaced DSGC dendrites could be influencing SAC crossovers in *VGlut2*^{Cre} cKO retinas, but DSGC dendrite lamination was preserved in RGC- and AC-specific cKO backgrounds, suggesting DSGC dendrites do not influence SAC dendritic crossovers. (**see Fig. S12E-F, lines 418-425**).

7- The author's observation of large gaps in the plexus following targeted deletion of Sema6a in RGCs is a key finding of the study. As mentioned in point 6, the exact cause of this phenotype is still not clear. A more detailed characterization of how these gaps form is needed. It would be particularly informative to investigate whether SAC arborization is affected in this condition. One possible cause for large gaps in the plexus might be defects in SAC symmetry. Additionally, live imaging would help determine the temporal relationship between the gap formation and the appearance of ectopic RGCs. This strategy could help discern between cause and consequence, clarifying the hierarchy of these events.

We agree that gap formation in this background is an interesting and important observation. However, it is not possible to examine SAC morphology in a Cre- dependent manner in a background also containing an RGC-specific Cre driver, so we were limited in our ability to study SAC outgrowth in combination with RGC-specific *Sema6a* cKO. Electric current-based targeted dye-filling, a technique that was not available to us at the time these cKO were performed, could enable this experiment. In our 2013 (Sun et al., 2013) study we established a link between SAC radial symmetry and plexus gaps, which we have strengthened here. We believe that RGC-derived Sema6A contributes to SAC dendrite outgrowth and symmetry, and we would like to explore the relationship between RGC and SAC dendrite arbor development during development in follow-on studies combining genetic and live imaging strategies, and using targeted dye-fill, a capability we

© 2024. Published by The Company of Biologists under the terms of the Creative Commons Attribution License (https://creativecommons.org/licenses/by/4.0/).

hope to establish in the Kolodkin laboratory.

8- It is unclear why the authors assert a significant role for Sema6a provided by RGCs on IPL stratification, given the relatively mild phenotype observed in the conditional knockout as shown in Figure 8A. While the authors suggest compensation by amacrine cells in this condition, this hypothesis is not substantiated with sufficient evidence.
Additional data supporting this compensatory mechanism would strengthen the authors' claims regarding the relevance of Sema6a from RGCs. Moreover, how do the authors reconcile the evidence for compensation in IPL stratification with the apparent lack compensation in plexus organization?

We agree the SAC IPL crossover phenotype in $VGlut2^{Cre}$ cKO retinas is mild compared to the SAC fusion phenotype of *Sema6a* null retinas. SACs exhibit crossovers when Sema6A is removed from RGCs, but not when Sema6A is removed from all SACs (using $Chat^{Cre}$ or $Megf10^{Cre}$, Fig.1) or in ACs using $Ptf1a^{Cre}$ (Fig.S11E), suggesting that SACs are not able to completely overcome the loss of Sema6A in RGCs for their lamination and that AC-derived Sema6A is normally dispensable for this process.

Regarding plexus organization, we do not think there is a lack of compensation in this process, *per se*. $VGlut2^{Cre}$ cKO retinas exhibit a well-defined ON SAC plexus, albeit with gaps, whereas the ON and OFF SAC plexuses are not discernable in $Ptf1a^{Cre}$; $VGlut2^{Cre}$ cKO retinas, similar to *Sema6a* null retinas. To fully ascertain compensation in this context we need to assess on plexus gaps in $Ptf1a^{Cre}$ cKO retinas, as indicated below.

Major Point (8) Action Item:

a) We provide new data quantifying ON-SAC plexus gaps in $Ptf1a^{Cre}$ cKO retinas to determine if AC-derived Sema6A normally contributes to ON SAC plexus organization. (**Please see Fig. 7F**).
b) We provide new data quantifying gaps in SAC-specific cKO retinas. (**See Fig. 7G**).

9- The authors should include detailed information about sample sizes and statistical analyses in the figure legends. This is crucial not only for experiments quantified but especially for those with qualitative evaluations, such as those depicted in Figures 5 and S8. In qualitative experiments where minor defects in IPL organization are observed, how did the authors exclude potential artefacts introduced by tissue sectioning? This concern is of relevance for the key experiments shown in Figure 8. Crossover needs to be quantified in all the conditions depicted in this figure to substantiate their conclusions.

We excluded sectioning artifacts throughout the study by examining and quantifying (where relevant) only in regions of interest in which morphology of the nuclear layers was intact. For instance, regions with damage to the photoreceptor layer (or other nuclear layer) were not included in any observations. We will address the reviewer's concerns as outlined below.

Major Point (9) Action Items:

a) We will add more information about sample sizes and statistical analyses to the figure legends and Materials and Methods as requested. (**see all figure legends for specific retinal N's and statistical analyses employed for quantifications; see lines 796-797, 799-804, 855-867, 810-814, and 820-822 for additional context related to quantifications**).
b) We provide new data quantifying crossovers using the parallel ChAT banding technique for each of the conditions in Fig. 8. (**see Fig. 8D).**

Minor Comments

1- The authors use scRNA-seq to validate the expression pattern of Sema6a in developing SACs. Although this dataset is an important resource for the community, it is unclear what it adds to the already detailed expression data presented in Figures 3 and S5. Moreover, the presentation of the scRNA-seq data does not effectively illustrate the expression pattern. The authors should streamline the description and the visual representation of this data.

Given our previous characterization of Sema6A protein expression *in vivo* and *in vitro* (Sun et al.,

© 2024. Published by The Company of Biologists under the terms of the Creative Commons Attribution License (https://creativecommons.org/licenses/by/4.0/).

2013), and also the apparent differential expression between OFF- and ON- SACs identified in that study, we felt it necessary to address the question of *Sema6a* expression from multiple angles. Since both the HA-Sema6A histological and *Sema6a*^{CreER} reporter characterizations relied on transgenics, we believe it is important to complement these results with an approach that examines endogenous expression in the absence of transgenic targeting at the *Sema6a* locus.

Minor Point (1) Action Item:
We have further streamlined the description of this data. **See lines 198-203.**

2- In Figure 1C, the germline deletion is termed Sox2Cre; Sema6aHA-F whereas in Figure 1E the null allele originating from a germline deletion is referred as Sema6a-/-. Given the complexity of the experiments, including schematics of the alleles and Cre recombination would greatly aid interpretation of the data, helping clarify the difference between the approaches used in S3B and S3C.

Data presented in Fig. 1C still contain the *Sox2*^{Cre} allele in the genetic background, whereas additional experiments moving forward (1E, 5, S3B, S3C) do not, and hence are referred to as with the nomenclature "-" to represent presence of a null allele.

3- The authors claim that the Sema6aHA-F allele produces a "fully functional Sema6a protein". It's important to note that the authors did not directly assess the functionality of the Sema6a protein at the molecular level. Instead, they should accurately describe what was observed in their control experiments, such as the absence of noticeable differences in the process of interest.

Minor Point (3) Action Item:
We have modified the text accordingly to describe the proper lamination of various retinal neurons achieved by HA-Sema6A **(lines 100-101)**. We have removed reference to the allele being "fully functional."

4- The authors concluded that "the Sema6aLacZ allele is hypomorphic", basing this on phenotypic differences between Sema6aLacZ and Sema6aHA-F. However, they do not perform direct comparisons in the current study. Comparisons and conclusions involving other studies should be relegated to discussion section. This is a recurring issue throughout the manuscript.

We based this observation on comparisons between our previously published *Sema6a*^{LacZ} phenotypes and the *Sema6a* null allele generated in this study.

Minor Point (4) Action Item:
We have moved this observation to the Discussion. **Lines 536-543.**

5- What distinguishes the experiments shown in Figures 2A and S3B? The authors note that in S3B: "The AAV reporter was unable to penetrate deeply enough to label sparse OFF-SACs, precluding quantification of OFF- SAC phenotypes." Does the approach used in Figure 2A not provide the necessary information to overcome this limitation? A clarification on how these two figures complement each other or provide different insights is needed.

Figure S3B examines more mature SACs at P42, whereas Fig. 2A characterizes SAC arbors at P14, a time point where SAC dendritic arbors are still expanding as mice and their retinas grow. Figure S3B demonstrates that the phenotypes observed in Fig. 2 are not corrected over time.

6- The authors claim that "Sema6A mRNA is expressed by postmitotic RGCs in the inner neuroblastic layer at E14.5". However, no RGC marker was used in the experiment to identify RGCs. Moreover, it is unclear how the authors define which cells are the SACs. The authors need to refine their conclusions to accurately reflect what was observed in their experiments. This issue of imprecision of the conclusions is a recurring concern throughout the manuscript and needs to be systematically addressed to enhance credibility of their findings.

This claim is based on published observations showing that RGCs are the earliest born retinal

© 2024. Published by The Company of Biologists under the terms of the Creative Commons Attribution License (https://creativecommons.org/licenses/by/4.0/).

neurons and establish the nascent GCL within the INBL, and that the majority of postmitotic neurons in the INBL at E15 are RGCs (Hinds and Hinds, 1978; Ray et al., 2018). Additionally, given that SACs are the earliest born amacrine cell type (Voinescu et al., 2009) and ON-SACs do not migrate into the INBL until ~E16 (Peng et al., 2020), we think that most, if not all, of the *Sema6a* ISH signal in the INBL corresponds to signal in RGCs. We cannot exclude *Sema6a* expression in the few non-SAC ACs likely to have migrated into the INBL by 14.5, however, so we have modified our statement regarding Fig. S10A accordingly.

Minor Point (6) Action Item:
We have modified this statement accordingly. **See lines 345-347.**

7- The authors discuss their findings in relation to a previous zebrafish study on RGC migration (Icha et al., 2016). They claim that RGC migration is preserved in Sema6A knockout and only fine-positioning is affected. They state: "When basal RGC migration is impaired, RGCs stall near the apical retina surface. Here, basal RGC translocation was entirely preserved." This statement raises several concerns. Without directly assessing RGC migration in their study, the basis for this conclusion remains unclear. Icha et al., 2016 demonstrated that interference with RGC migration causes RGCs to halt at various positions, including at positions near the RGC layer. The presence of RGCs at the INL in the Sema6A knockout could indicate a delay in their migration, as RGCs might not be able to cross the emerging IPL, as shown for horizontal cells in zebrafish (see https://doi.org/10.7554/eLife.76408). Additionally, extrapolating findings from zebrafish to mice is problematic, it is not clear whether RGCs in mice undergo the same migration phases. The authors should thoroughly review the manuscript to correct similar issues of imprecision in their statements.

While we did not assess migration by live cell imaging, we did assess terminal positioning of RGCs, which has historically been used as a readout of gross migration defects (e.g., BrdU assessments of migration). We never detected RBPMS$^+$ RGCs apical to the OFF-SAC layer within the INL, which represents a position that in embryonic development corresponds to the boundary between the ONBL and the INBL (see Ray et al., 2018, and Peng et al., 2020). We envision RGCs interacting with dendrites from SACs and RGCs in the nascent IPL at this boundary and integrating those cues prior to migrating through the IPL and settling in their final position within the GCL. We see a distinction here between basal migration through the ONBL and navigating the IPL/INBL, like the switch in migratory modes at E16 undertaken by newly specified ON-SACs in order to migrate into the INBL, as described by Peng et al., 2020. We agree it is very likely that RGCs in the INL of *Sema6a* null retinas are prohibited from crossing the IPL, likely due to loss of attractive signaling in the absence of *Sema6a* that promotes crossing the IPL, or the presence of a new inhibitory environment that prevents RGC crossing.

Minor Point (7) Action Item:
We have modified the text to include these possibilities and dampen our conclusion about basal migration. We provide a new experiment examining RGC positioning in neonatal retinas (E18.5-P0.5) and observed no RGCs within the ONBL (**see Fig. 5M-N**). Instead, RGCs appear to stack at the IPL/ONBL border. We have modified our Discussion, focusing on this finding and the possibility that Sema6A contributes to final steps in migration directly, or that a novel inhibitory boundary is established by mis- projecting neurites in *Sema6a*$^{-/-}$ retinas that prevents the final positioning of some RGCs. Please see lines 509-516.

8- The manuscript dedicates an entire section of the results to discussing the data presented in Figure S8. The authors should consider condensing the description and integrating it with other relevant sections for a more streamlined presentation.

Thank you for this suggestion.

Minor Point (8) Action Item:
We have removed contemplations of Plexin-dependent and -independent Sema6A signaling pathways from the discussion, and briefly summarize findings as related to the primary data. **Please see lines 302-303, 312-314, and 319-322**.

© 2024. Published by The Company of Biologists under the terms of the Creative Commons Attribution License (https://creativecommons.org/licenses/by/4.0/).

REVIEWER #3

We thank his reviewer for their comments on our study and their assessment that it is interesting, carefully done, and should be of substantial interest to the field.

"Altogether, this work is very carefully done with impressive genetic tools, and is likely to be of substantial interest to developmental neurobiologists as it advances our understanding of retinal circuit development as well as the function of the important guidance molecule Sema6a."
We appreciate the points raised by this reviewer and have done our best to address them in order to strengthen our study.

Comments for the Author:
There are some areas in which the paper could be improved:

1. The authors imply in the results (p.13) and discussion (p. 17, second paragraph) that gaps in the ON SAC plexus are caused by RGC misplacement. However, there is insufficient evidence provided that misplaced RGCs align preferentially with the gaps. An alternative interpretation is that the gaps are related to SAC dendrite morphogenesis phenotypes (as shown in Fig. 2A and Fig. S3A-B). The second possibility is perhaps the more likely one, given the relationship of dendrites to holes shown in Fig. S3B, and given that other mutants with defects in SAC dendrite arrangement (e.g. Pcdhg and Megf10 mutants) also exhibit SAC plexus holes (without any RGC misplacement). If the authors wish to keep these passages as currently written, they should provide quantitative evidence that misplaced RGCs are selectively associated with SAC plexus holes.

We apologize – we did not mean to suggest that displaced RGCs are exclusively responsible for ON plexus gaps. Our intention was to present the possibility that gaps could be influenced by displaced retinal neurons and/or their neurites. We agree it is entirely possible that gaps correspond to SAC dendritic arbor outgrowth deficits when *Sema6a* is selectively removed from RGCs (evidence of gaps/radial symmetry deficits in *VGlut2^{Cre}* RGC cKO retinas, but not in *Chat^{Cre}/Megf10^{Cre}* cKO retinas), and we would like to investigate that possibility in a future study. We were not able to study this directly here owing to the need to remove *Sema6a* Cre-conditionally from RGCs while simultaneously Cre-conditionally labeling SACs to examine their dendritic arbor morphology--we are able to exclusively label SAC arbors with two Cre drivers in the same background. This would be possible with electric current-based targeted dye- filling, and the Kolodkin lab is in the process of acquiring this technology in order to perform such experiments, but this will have to be a future direction. We have modified the text in the Discussion to include the possibility of SAC arbor outgrowth deficits contributing to ON plexus gap formation in RGC cKO retinas.

Major Point (1) Action Item:
We have modified the text to explicitly include the possibility that some ON plexus gaps in *VGlut2^{Cre} Sema6a* cKO retinas are caused by SAC arbor outgrowth defects. Our additional analyses of plexus gaps in pan-AC and SAC-specific cKO backgrounds have allowed us to tease apart gaps that are a result of SAC self-avoidance defects (cell- autonomous roles of Sema6a), and cell-non-autonomous roles for Sema6A **(see Fig.** 7F-G). See lines 383-392; also Discussion, lines 484-493.

2. p.15: I am not aware of any paper confirming that Ptf1a-Cre efficiently drives recombination in SACs. This confirmation is needed for the conclusion of Fig. 8A, bottom - "Removing Sema6a from all ACs (Ptf1aCre) highlights the robust expression of Sema6A in DSGC dendrites." The authors should assess Cre reporter expression in SACs to rule out the possibility that Sema6A is expressed by remaining SACs that did not express Cre.

Major Point (2) Action Item:
We have provided a new supplemental panel demonstrating that *Ptfa1^{Cre}* drives recombination of the *ROSA-LSL^{tdTomato}* reporter in ON- and OFF-SACs. **(See Fig. S11D)**

3. p. 17: The authors' model for the RGC positioning phenotype is that there is a defect in radial migration which affects the cell's choice of final location in which to settle. But because only adult timepoints were analyzed, it is also possible that cells migrated normally to the GCL

© 2024. Published by The Company of Biologists under the terms of the Creative Commons Attribution License (https://creativecommons.org/licenses/by/4.0/).

but then later flipped back into the INL. The latter scenario is plausible because Sema6A is localized to the IPL, where it could serve as a repulsive cue that prevents RGCs from moving into the INL during postnatal development. To support their model the authors should show that RGCs are already mislocalized during or shortly after RGC radial migration is complete – i.e. embryonic or early postnatal (e.g. P0) timepoints.

Our interpretation is that radial migration is intact in *Sema6a* null retinas, but the RGC migratory behavior that regulates navigating through the IPL to reach the GCL and ultimately facilitate mosaic spacing is compromised. The reviewer's suggestion to examine RGC mislocalization earlier in development would strengthen our claims that final RGC migratory behavior of some RGCs is compromised.

Major Point (3) Action Item:
We have examined RGC mislocalization in neonate *Sema6a* null retinas to discern if RGCs are mislocalized just after their specification/migration is complete, as suggested, and provide new data regarding RGC localization in neonate retinas. **(see Fig. 5M-N)**

4. The model figure in Fig. 8E was a very nice summary of the complex genetics and resulting phenotypes. However, it needs further explanation so that readers – especially those not deeply immersed in this field – can really appreciate why these are the paper's key conclusions. I would strongly suggest that the authors devote a major part of the Discussion to go through in some detail the evidence supporting the conclusions that are illustrated in this figure. It would make a big difference to the reader's ability to understand how all the disparate findings in the results may be synthesized into a coherent story.
As the discussion stands now, there are a lot of speculative passages about next steps or big picture models – e.g. discussion of ON SAC plexus gaps and whether these are linked to the RGC laminar defects (p. 16, paragraphs 1 & 2); and the discussion of the SAC crossover phenotype (paragraph beginning on the 2nd-to-last line of p. 18). In my view, clarity of the manuscript would be well served by removing these speculative passages and replacing them with a more concrete delineation of the significance of the present findings.

Major Point (4) Action Item:
Thank you for these comments. Please see the considerable changes to the Discussion, modified to track the model more closely, as suggested. We removed some passages and emphasized significance of these data in the following discussion sections correspond to the model as follows:

"Sema6A-PlexA2 Signaling in All SACs Regulates Dendritic Arbor Development" - Fig. 8F, top-left

"Sema6A is Required for RGC Migration and Settling in the GCL" - Fig. 8F, top right

"Sema6A in RGCs Delimits the ON Domain of the IPL" - Fig. 8F, bottom

Minor Comments:

1. Figure 1 could benefit from a model or schematic figure illustrating circuit anatomy -- perhaps incorporating figure S1A and/or S1E into the main figures? Having some visuals would be helpful as a complement to the text within the introduction describing the DS circuits.

Thank you for the suggestion – we have adjusted Fig. 1 to incorporate a model.

Minor Point (1) Action Item:
We have modified Fig. 1 and Fig. 2 to incorporate models describing anatomy of the phenotypes examined in those figures. **(see Fig. 1C, 1F, and Fig. 2A)**

2. p. 4: the sentence starting with: "However, the cell types in which…" is awkward and unclear.

Minor Point (2) Action Item:
We have restated this. **(see line 86-87).**

© 2024. Published by The Company of Biologists under the terms of the Creative Commons Attribution License (https://creativecommons.org/licenses/by/4.0/).

3. Fig. S6 was very interesting but there were several aspects of figure layout that were confusing. The authors should:
-Flip the color/expression scales so that 0 is at the bottom at 1 is at top on all panels. The current version led to confusion as to which color represents "low" vs. "high" expression.
-Label the clusters more extensively within all panels – e.g. with ages and possibly with text to mark ON and OFF clusters (the asterisks were confusing)
-In panels H-J, it impossible to see the averages, error bars, and colored lines. The lines should be made larger and more noticeable.
-H-J: the blue vs. green color distinction between the ON and OFF groups is hard to discern.
-Legibility of some smaller text could be improved with higher resolution graphics.

Minor Point (3) Action Item:
We have incorporated as many of the reviewer's suggestions as possible to improve this figure and hope this satisfies these concerns. **(see modified Fig. S6)**

4. For reviewer convenience the authors could use line numbers in their next submission.

Minor Point (4) Action Item:
We have added line numbers in this revision.

5. P.23: The description of the Sema6a-CreER mouse line should reference Fig. S5A, which has a drawing of the allele structure and features.

Minor Point (5) Action Item:
We have referenced Fig. S5A when describing the *Sema6a*^{CreER} mouse line. **(See line 198-199)**.

6. P.24, section labeled "SAC Culture": There are references to "Fig. SX" which need to be fixed with the appropriate figure reference.

Minor Point (6) Action Item:
We have corrected the reference to Fig. SX in the text. **(see Lines 672 and 679).**

7. P.26: The RPM value for centrifuging and pelleting cells is given. Typically, these methods are given in units of RCF. If RPM values are used, the authors should also state the brand/model of centrifuge and rotor that were used.

Minor Point (7) Action Item:
We have added the RCF value used to the Materials & Methods SAC culture description. **(see line 745).**

Resubmission

First decision letter

MS ID#: DEVELOP/2024/204293

MS TITLE: Retinal Ganglion Cell-derived Semaphorin 6A Segregates Starburst Amacrine Cell Dendritic Scaffolds to Organize the Inner Retina

AUTHORS: Alex Kolodkin, Rebecca E. James, Natalie R. Hamilton, Lola Nicole Huffman, Victoria N. Neckles, R. Jeroen Pasterkamp and Loyal A. Goff

You will be pleased to read that the reviewers are mostly happy with your revisions and their are just a few corrections and minor issues to consider ahead of publication. Please attend to all of the reviewers' comments in your revised manuscript and detail them in your point-by-point response. If you do not agree with any of their criticisms or suggestions explain clearly why this is so.

© 2024. Published by The Company of Biologists under the terms of the Creative Commons Attribution License (https://creativecommons.org/licenses/by/4.0/).

Reviewer 1

Advance summary and potential significance to field

The authors have done a good job revising the manuscript as per reviewer feedback. I have no further comments. I would recommend a careful read through of the manuscript to correct typographical errors that appear in this version.

Reviewer 2

Advance summary and potential significance to field

James et al. have performed a remarkably thorough genetic analysis of how Sema6A signaling influences retinal development. The authors generate several new Sema6a alleles, including the first true null allele as well as a conditional floxed allele; and also take advantage of numerous existing alleles that enable dissection of Sema6a forward and reverse signaling through its binding partners Plexin A2 and A4. With this array of genetic tools they methodically delineate multiple roles for Sema6a in the positioning of developing retinal neurons, arbors, and synapses. Key findings include:

- Sema6a has multiple cell-autonomous and nonautonomous roles in development of starburst amacrine cell (SAC) dendrites. Dendritic self-avoidance is a cell-autonomous function, but most of the severe SAC phenotypes previously reported for Sema6a mutants, including the failure to segregate OFF and ON SAC dendrites into separate sublayers, involve Sema6a produced by retinal ganglion cells (RGCs).

- Using a HA-tagged knock-in Sema6a allele, the authors resolve prior uncertainty about where and when Sema6a protein is in fact expressed. They confirm that ON SACs express Sema6a, as previously reported by the Kolodkin lab, but they also find, surprisingly, that OFF SACs also express it. And they show that OFF SACs also exhibit dendritic patterning phenotypes, showing that Sema6a functions in both SAC cell types. This is an important update of this lab's previous work on Sema6a in SACs.

- When Sema6a is deleted from RGCs, it becomes upregulated in a compensatory manner by amacrine cells. Thus, to phenocopy the null allele, one must delete Sema6a from both RGCs and amacrine cells. This finding highlights a surprising compensatory mechanism whereby retinal neurons that do not normally express Sema6a can somehow sense that they need to upregulate this repulsive extracellular signaling molecule.

- Sema6A has general roles in organizing the ON sublayer of the IPL neuropil. When Sema6a is absent, many types of retinal neurons that normally avoid ON IPL begin to innervate these IPL regions.

- Sema6A is required to confine retinal ganglion cells to the GCL. In Sema6a null mutants, about 30% of RGCs become displaced to the INL.

The manuscript is clearly written, which is important given that the reader is being asked to keep track of many different genetic manipulations and phenotypes. Overall this work provides an important contribution to our understanding of cell-cell communication in the assembly of the retinal neuropil.

Comments for the author

I have only one minor comment: Fig. 7G appears to be mislabeled. The graph shows data from Megf10-cre deletion but it is labeled Ptf1a-cre (i.e. label is identical to 7F).

Reviewer 3

© 2024. Published by The Company of Biologists under the terms of the Creative Commons Attribution License (https://creativecommons.org/licenses/by/4.0/).

Advance summary and potential significance to field

The authors made text changes to address issues of imprecision and to improve clarity of the manuscript. They also included additional quantifications and experiments to substantiate their conclusions. However, their main conclusion that Sema6a in RGCs plays a pivotal role in SAC dendrite scaffold segregation remains unsupported. In my original review, I pointed out the need for additional experiments to support this claim:

"It is unclear why the authors assert a significant role for Sema6a provided by RGCs on IPL stratification, given the relatively mild phenotype observed in the conditional knockout as shown in Figure 8A. While the authors suggest compensation by amacrine cells in this condition, this hypothesis is not substantiated with sufficient evidence. Additional data supporting this compensatory mechanism would strengthen the authors' claims regarding the relevance of Sema6a from RGCs."

It remains unclear why the authors favor the interpretation that Sema6a in RGCs plays a pivotal role in SAC dendrite scaffold segregation over a model in which Sema6a from various cell types, including RGCs and ACs, ensures proper retinal patterning. Did the authors consider the possibility that Sema6a in RGCs might compensate for the loss of Sema6a in ACs in the conditional experiments performed using Ptf1aCre?

In my original review, I also pointed out the use of heterozygous animals as controls, despite the described haploinsufficiency of Sema6a. The authors provided clear justifications for use of heterozygous animals as controls. The interpretation of the results shown in Figure S11C takes into account the effects of haploinsufficiency. However, in contrast, there is no mention of haploinsufficiency in relation to the data shown in Figure 7C. Could the authors clarify if this data has been corrected for the expected effects of haploinsufficiency to accurately reflect the phenotype resulting from Sema6a deletion in RGCs? The presentation of this data may need to be amended.

Comments for the author

Minor issues

Could the authors please clarify or address the minor issues listed below?

In Line 299, the authors mentioned, "We analyzed retinal phenotypes in Plxna2-/-; Plxna4-/- retinas, and SAC lamination defects were no more severe than in Plxna2-/- retinas (Fig. S8C)". However, it appears that S8C does not include data for Plxna2-/- retinas.

The identification of Cre lines in Figure 7G appears to be incorrect.

In Line 398, the authors concluded "Thus, both forward and reverse RGC-derived Sema6A signaling contribute to RGC settling," However, the reasoning behind this conclusion is unclear, as Figure 7C suggests that the effect is primarily due to reverse signaling.

In Line 110, the authors stated "To assess cell-type specific roles for Sema6A, we crossed the Sema6aHA-F allele to a germline Cre driver (Sox2Cre ; Hayashi et al., 2002)." However, using a germline Cre driver does not enable conditional inactivation in specific cell types.

First revision

<u>Author response to reviewers' comments</u>

We thank the reviewers for their mostly positive comments on this second revision of our manuscript—reviewers 1 and 2 (formally #3) are satisfied that we have addressed their

© 2024. Published by The Company of Biologists under the terms of the Creative Commons Attribution License (https://creativecommons.org/licenses/by/4.0/).

concerns on our first revision. We appreciate Reviewer #2's additional comments, and we address them below.

<u>REVIEWER #3</u> (***formerly #2***): The authors made text changes to address issues of imprecision and to improve clarity of the manuscript. They also included additional quantifications and experiments to substantiate their conclusions. However, their main conclusion that Sema6a in RGCs plays a pivotal role in SAC dendrite scaffold segregation remains unsupported. In my original review, I pointed out the need for additional experiments to support this claim:

"It is unclear why the authors assert a significant role for Sema6a provided by RGCs on IPL stratification, given the relatively mild phenotype observed in the conditional knockout as shown in Figure 8A. While the authors suggest compensation by amacrine cells in this condition, this hypothesis is not substantiated with sufficient evidence. Additional data supporting this compensatory mechanism would strengthen the authors' claims regarding the relevance of Sema6a from RGCs."

It remains unclear why the authors favor the interpretation that Sema6a in RGCs plays a pivotal role in SAC dendrite scaffold segregation over a model in which Sema6a from various cell types, including RGCs and ACs, ensures proper retinal patterning. Did the authors consider the possibility that Sema6a in RGCs might compensate for the loss of Sema6a in ACs in the conditional experiments performed using Ptf1aCre?

To clarify our interpretation, we have modified the discussion to lay out the following points for consideration. <u>Please see lines 545 - 560.</u>

- ***Crossovers were not detected in AC-cKO retinas, suggesting that ACs do not normally contribute to SAC lamination***
- ***RGC displacement was not detected in AC-cKO retinas, suggesting that ACs do not normally contribute to RGC settling***
- ***AC-cKO severely disrupts the ON-SAC plexus, demonstrating that AC-cKO is sufficient to disrupt retinal development and highlighting a specific role for AC-derived Sema6A in SAC dendritic arbor elaboration***
- ***Sema6A is expressed by more RGCs than ACs neonatally - it is enriched in the proposed SAC segregating cell type***
- ***Sema6A is asymmetrically enriched in DSGC dendrites in the early postnatal retina, positioning RGC-derived Sema6A to segregate the SAC scaffolds***
- ***There is compensatory upregulation of Sema6A in RGC-cKO, but not AC-cKO, retinas***
- ***Impacts of RGCs on AC neurite organization are established in zebrafish***

We consider these points important for addressing the issues raised above by this Reviewer, highlighting data we already present in our study providing support for the model we present.

In my original review, I also pointed out the use of heterozygous animals as controls, despite the described haploinsufficiency of Sema6a. The authors provided clear justifications for use of heterozygous animals as controls. The interpretation of the results shown in Figure S11C ***<u>11F?</u> [we believe that this is the panel the reviewer is referring to]*** takes into account the effects of haploinsufficiency. However, in contrast, there is no mention of haploinsufficiency in relation to the data shown in Figure 7C. Could the authors clarify if this data has been corrected for the expected effects of haploinsufficiency to accurately reflect the phenotype resulting from Sema6a deletion in RGCs? The presentation of this data may need to be amended.

We have addressed this in the text and added a new quantification demonstrating that RGC displacement in cVGlut2 cKO retinas is significantly greater than Sema6a+/- retinas. <u>Please see lines 384-386.</u>

Minor issues

© 2024. Published by The Company of Biologists under the terms of the Creative Commons Attribution License (https://creativecommons.org/licenses/by/4.0/).

Could the authors please clarify or address the minor issues listed below?

In Line 299, the authors mentioned, "We analyzed retinal phenotypes in Plxna2-/-; Plxna4-/- retinas, and SAC lamination defects were no more severe than in Plxna2-/- retinas (Fig. S8C)". However, it appears that S8C does not include data for Plxna2-/- retinas.

We added an additional reference to panel S8F. Please see line 302 and Sun et al., 2013.

The identification of Cre lines in Figure 7G appears to be incorrect. ***Thank you, we corrected this.***

In Line 398, the authors concluded "Thus, both forward and reverse RGC-derived Sema6A signaling contribute to RGC settling," However, the reasoning behind this conclusion is unclear, as Figure 7C suggests that the effect is primarily due to reverse signaling.
Thank you - the intention in this sentence was apparently lost in editing. The text was corrected. Please see lines 402-404.

In Line 110, the authors stated "To assess cell-type specific roles for Sema6A, we crossed the Sema6aHA-F allele to a germline Cre driver (Sox2Cre ; Hayashi et al., 2002)." However, using a germline Cre driver does not enable conditional inactivation in specific cell types.

We have added additional context to address this. Please see lines 110-111.

Second decision letter

MS ID#: DEVELOP/2024/204293

MS TITLE: Retinal Ganglion Cell-derived Semaphorin 6A Segregates Starburst Amacrine Cell Dendritic Scaffolds to Organize the Inner Retina

AUTHORS: Alex Kolodkin, Rebecca E. James, Natalie R. Hamilton, Lola Nicole Huffman, Victoria N. Neckles, R. Jeroen Pasterkamp and Loyal A. Goff
ARTICLE TYPE: Research Article

I am happy to tell you that your manuscript has been accepted for publication in Development, pending our standard publication integrity checks.

© 2024. Published by The Company of Biologists under the terms of the Creative Commons Attribution License (https://creativecommons.org/licenses/by/4.0/).
